# Molecular Phylogeny, Diversity and Zoogeography of Net-Winged Beetles (Coleoptera: Lycidae)

**DOI:** 10.3390/insects9040154

**Published:** 2018-11-01

**Authors:** Michal Masek, Michal Motyka, Dominik Kusy, Matej Bocek, Yun Li, Ladislav Bocak

**Affiliations:** 1 Laboratory of Molecular Systematics, Department of Zoology, Faculty of Science, Palacky University, 71146 Olomouc, Czech Republic; Michal.Masek@seznam.cz (M.M.); motyka01@gmail.com (M.M.); dominik.kusy2@gmail.com (D.K.); bocema00@gmail.com (M.B.); liyun25@mail2.sysu.edu.cn (Y.L.); 2 State Key Laboratory of Biocontrol, College of Ecology and Evolution, School of Life Sciences, Sun Yat-sen University, Guangzhou 510275, China

**Keywords:** Coleoptera, Elateroidea, Lycidae, molecular phylogeny, zoogeography, zoogeographic realms, zoogeographic boundaries, diversity

## Abstract

We synthesize the evidence from molecular phylogenetics, extant distribution, and plate tectonics to present an insight in ancestral areas, dispersal routes and the effectiveness of geographic barriers for net-winged beetle tribes (Coleoptera: Lycidae). Samples from all zoogeographical realms were assembled and phylogenetic relationships for ~550 species and 25 tribes were inferred using nuclear rRNA and mtDNA markers. The analyses revealed well-supported clades at the rank of tribes as they have been defined using morphology, but a low support for relationships among them. Most tribes started their diversification in Southeast and East Asia or are endemic to this region. Slipinskiini and Dexorini are Afrotropical endemics and Calopterini, Eurrhacini, Thonalmini, and Leptolycini remained isolated in South America and the Caribbean after their separation from northern continents. Lycini, Calochromini, and Erotini support relationships between the Nearctic and eastern Palearctic faunas; Calochromini colonized the Afrotropical realm from East Asia and Metriorrhynchini Afrotropical and Oriental realms from the drifting Indian subcontinent. Most tribes occur in the Oriental and Sino-Japanese realms, the highest alpha-taxonomic diversity was identified in Malesian tropical rainforests. The turn-over at zoogeographical boundaries is discussed when only short distance over-sea colonization events were inferred. The lycid phylogeny shows that poor dispersers can be used for reconstruction of dispersal and vicariance history over a long time-span, but the current data are insufficient for reconstruction of the early phase of their diversification.

## 1. Introduction

The dispersal propensity and ecological adaptability substantially affect the extant distribution of animals and therefore, the zoogeographic analyses of highly mobile animals with diverse life history are often obscured by long distance re-colonization events and range shifts in dynamically changing environments [1,2,3]. Studies based on model groups with different biological characteristics are needed to test the validity of zoogeographical hypotheses. Molecular systematics should provide a robust phylogeny for zoogeographic studies and the recent progress makes feasible the studies with hundreds to thousands of analyzed species [4,5,6,7].

Here, net-winged beetles (Coleoptera: Lycidae) are studied and we focus on the delimitation of tribes, and the evaluation of species richness and distribution. We propose that these beetles can serve as a model characterized by low dispersal propensity and ecological uniformity [8,9,10,11,12,13]. Altogether, over 4200 lycid species have been described and most diverse are Metriorrhynchini, Platerodini, Lycini, Calopterini, and Calochromini, each of them with at least several hundred species [14,15]. Net-winged beetles have been reported from forest and shrub habitats of all continents. Their larvae live in upper soil layers rich in organic debris, decaying roots in deeper soil, rotten tree trunks or dead branches in the canopy of rain and cloud forests [8]. Their unique complex mandibles are adapted for sucking up liquids containing rich microbial life [8,16,17]. Therefore, they are strictly limited to habitats where moisture is available in decaying organic substances, at least seasonally. A prolonged dry season considerably limits their abundance and only drought tolerant Calochromini and Lycini occur in higher numbers in such areas [15,18].

Lycidae is one of morphologically similar soft-bodied elateroid families, a polyphyletic assemblage earlier designated as cantharoids [19,20,21]. They share incomplete sclerotization [22,23,24] which limits their resistance to desiccation due to exposed inter-segmental membranes, an absent sub-elytral cavity, and a delicate cuticle [14,25]. Their soft integument provides an inadequate framework for flight muscles. Although most lycids are winged, they fly slowly, in short distances, usually avoid open windy and sunny places and remain inactive on leaves under forest canopy [14,26]. As a consequence, they are poor dispersers. Sklenarova et al. [11] showed that Metriorrhynchini only seldom crossed sea straits and Li et al. [27,28,29] identified high species turn-over between geographically close regions despite the absence of apparent dispersal barriers. Neotenic females further decrease the dispersal propensity of some lycids as they retain the larval morphology when mature [12,13,30,31,32,33,34]. Their dispersal propensity is extremely limited, and, despite their ancient origin, their ranges are very restricted [9,10,13,23].

**Figure 1:**
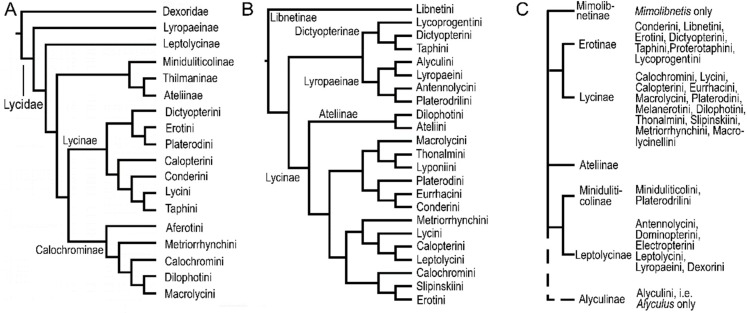
Overview of net-winged beetle classification. (**A**) Kazantsev (2005) [17]; (**B**) Bocak et al. (2008) [23]; (**C**) Kazantsev (2013) [35].

The classification of net-winged beetles remains contentious and mutually incompatible topologies have been discovered by earlier morphological and molecular analyses (Figure 1) [14,15,17]. Similarly, their distribution has not been critically analyzed. The aim of this study is to recover the phylogeny of net-winged beetles with currently available molecular data and to discuss some not yet indicated relationships inferred from the molecular phylogeny. Further, we focus on the distribution of tribes and try to define the areas with high phylogenetic and alpha-taxonomic diversity and estimate the effectiveness of dispersal barriers.

## 2. Material and Methods

### 2.1. Sampling

Altogether, 766 samples and ~550 morphospecies were analyzed (Table 1 and Table S1). About 1900 sequences were taken from previous studies, e.g., the previous analysis of Lycidae containing 69 species [23] and several taxonomically restricted studies [11,12,13,18,26,27,28,29]. Additional 800 fragments were newly produced to include earlier omitted lineages. The samples represent all major lineages except a few recently described species-poor tribes of an uncertain origin (Table S2).

### 2.2. Laboratory Methods

Genomic DNA was extracted from thoracic muscles using Wizard SV96 kit (Promega Inc., Madison, WI, USA) and the yield was measured using a spectrophotometer Nanodrop-1000. PCR amplifications were performed in a 50 μL reaction volume using 0.5 U Taq polymerase, 1 mM MgCl_2_, 50 mM of dNTPs, 0.2 mM of each primer (Table S3), and typically 30 ng of template. Cycle conditions were 2 min at 94 °C, 30–60 s at 94 °C, 30–60 s at 45–52 °C, 1–2 min at 72 °C (Steps 2–4 repeated 35–40 times), and 10 min at 72 °C. We sequenced the complete nuclear 18S rRNA, D2 region of 28S rRNA and three mitochondrial DNA fragments (*rrnL*, *cox1-tRNA-Leu-cox2*, and *nad5-tRNAs* mtDNA, all further referred as *rrnL*, *cox1* and *nad5* mtDNA). PCR products were purified using PCRμ96 Plates (Millipore Inc., Burlington, MA, USA) and sequenced by an ABI3130 sequencer using the Big Dye Terminator Cycle Sequencing Kit 1.1.

### 2.3. Sequence Handling and Phylogenetic Analyses

Sequences were edited using Sequencher 4.8 (Gene Codes Corp., Ann Arbor, MI, USA). All fragments were aligned using MAFFT 7 under default settings [36]. The alignments of the protein-coding genes *cox1*, *cox2*, *nad1*, and *nad5* were checked by amino acid reading frames to avoid shifts in codon positions and paralogs. All separately aligned fragments were concatenated in a supermatrix. The ingroup was aligned alternatively with the set of 89 outgroups and with Iberobaeniidae as a single outgroup. These two datasets were prepared to test the effect of the alignment on topology, as several outgroups have long insertions in the length variable loops of rRNA. Both supermatrices were analyzed under the maximum likelihood (ML) criterion using IQ-TREE 1.6.0 [37] with 5000 UFboot iterations and partitioned by genes, then with the same algorithm, but using the -bnni option to reduce the risk of overestimating branch supports with UFBoot due to severe model violations. Further, both datasets were analyzed using the RAxML 8.2.10, with separate parameters applied to each partition, and confidence determined with 1000 bootstrap pseudoreplicates utilizing the rapid bootstrap option under the GTRCAT substitution model. Additionally, the separate rRNA and mtDNA subsets were analyzed using IQ-TREE 1.6.0, partitioned by genes, with 5000 UFboot iterations and using the -bnni option. Optimal models of molecular evolution and the partition scheme were identified by ModelFinder [38] implemented in IQ-TREE. All models and partitions are listed in Table S4. The resulting phylogenetic trees were visualized using FigTree 1.4.2 [39].

### 2.4. Geographical Distribution and Ancestral State Reconstruction

Distribution data were assembled from the catalogue [15] and Zoological Record database until the end of 2014 (Thomson Reuters Inc., Albuquerque, NM, USA). Distribution maps for the family and tribes were produced using the free vector map of the world (http://www.naturalearthdata.com/downloads/10m-physical-vectors/). Geographical coordinates for each species were edited in a csv file and analyzed on a 2-degree vector grid. Final charts were visualized in the open source Geographic Information System QGIS Desktop 2.10.1 (https://www.qgis.org/en/site/forusers/download.html).

**Figure 2:**
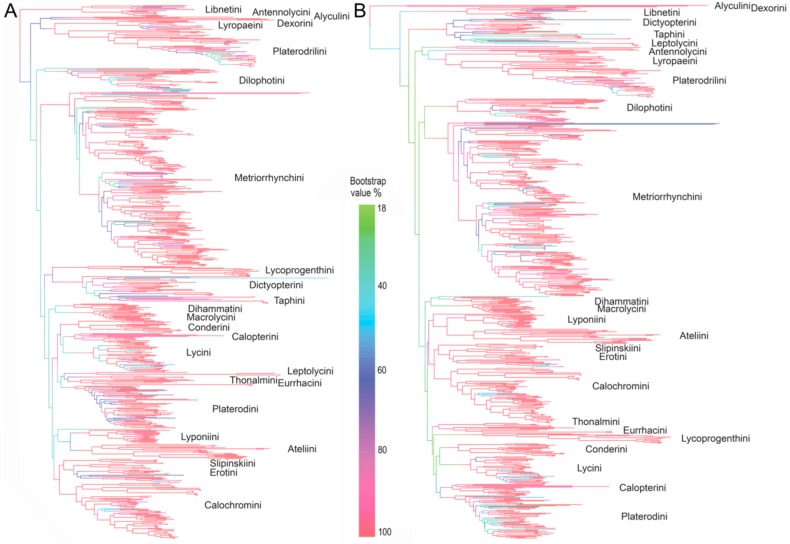
The maximum likelihood IQ-TREE topology. (**A**) Tree inferred from the complete dataset and 89-taxa outgroup; (**B**) tree inferred from the same dataset and a single outgroup (Iberobaeniidae). Colors designate a level of the bootstrap support; outgroups omitted.

The ambiguous relationships and a low support for basal splits exclude the reconstruction of an ancestral area for whole family and therefore, we defined 20 clades with bootstrap support (BS) ≥99% in the IQ TREE analysis (Figure 2) and robustly supported by morphology. Only Libnetini, Ateliini, and Dihammatini did not contain all terminals. In two later cases, a single terminal was placed at the base of their sister clade; these terminals were omitted from phylogeographic analyses. Further, we did not formally analyze tribes represented by a low number of taxa: Alyculini (1 sp.), Antennolycini (2 spp.), Dexorini (2 spp.), Leptolycini (4 spp.) and Taphini (7 spp.). Additionally, the first four tribes are known from a single realm and Oriental Taphini contain a single Oceanian species.

The ancestral state reconstructions were performed using discreet traits in BEAST 2.3.0 [40]. The separate datasets were produced for the tribe-level monophyla and outgroups were omitted. The best model of evolution for Bayesian analysis was selected as above. The analyses were set for 50 million generations, coalescent model, and constant population size as recommended in the manual [41]. We defined the following areas: Neotropical, Panamanian, Nearctic realms; Afrotropical realm: Continental; AFR: Madagascar; Sino-Japanese realm: Continental (China, Taiwan, Hainan), S-J: Japan; Oriental realm: Indo-Burma; OR: India; OR: Sundas (Java, Sumatra, Borneo), OR: Philippines, OR: Malay Peninsula, OR: Wallacea; Oceanian realm: New Guinea and adjacent islands; Australian realm, Palearctic: West Palearctic, PAL: East Palearctic. The maximum credibility tree was generated using TreeAnnotator 2.2.0 [42]. Ancestral areas and directions of the dispersal for each tribe were visualized using SPREAD 1.0.6 [43]. The analyses of dispersal history were not repeated for Ateliini, Calochromini, Dilophotini and Metriorrhynchini, whose phylogeography has already been recently analyzed [10,11,18,26].

## 3. Results

### 3.1. Sampling of the Diversity

The complete dataset contained ~550 net-winged beetle species of Lycidae and 89 outgroups (Table S1); the same dataset was alternatively analyzed with the Iberobaeniidae as a single outgroup (Figure 1, Figure 2 and Figures S1–S9). The concatenated dataset contained 18S rRNA (569 terminals), 28S rRNA (567), *cox1* (699), *rrnL* (695) and *nad5* mtDNA (663). The mtDNA genes were AT biased (A: 32.43%, C: 13.04%, G: 13.50%, T: 41.03%); the rRNA fragments had balanced representation of nucleotides (A: 24.79%, C: 23.87%, G: 29.36%, T: 21.98%).

The dataset contained 10 genes or their fragments, data for 6182 fragments were included in the analysis, 2108 fragments were missing, i.e., 25.4%. The samples represented 25 tribes; 7 tribes, combined representing 11 species, were unavailable (Table S2). The sequenced species represent ~13% of the known diversity. Most species were identified to the genus-level as alpha-taxonomy remains chaotic and species-level identification is impossible without comparison with primary types. Therefore, unidentified morphospecies were considered when samples were included in the analysis. All zoogeographical realms were sampled approximately proportionate to their diversity (Table 1 and Table S1) and the geographic origin is reported for all samples (Table S1, Figure S1). 

### 3.2. The Tree Topology

All analyses returned Lycidae as a monophylum (BS 78–100%, Figure 2, Figures S1, S4, S6, S8 and S9). The relationships among subfamilies and tribes were highly unstable and sensitive to the outgroup selection, algorithm and settings. The topologies inferred from the analyses with 89 outgroup taxa are shown in Figure 2A, Figures S1, S4, S6, S8 and S9. If *Iberobaenia* Bocak et al., was used as a single outgroup, the topology also significantly changed (Figure 2B, Figures S3, S5 and S7). Conversely, the monophyly of most tribes as morphologically defined [14,44,45] was recovered in almost all analyses and they obtained BS 78–100% in the IQ-TREE and RAxML analyses (Figure 1, Figure 2 and Figures S1–S7). The support remains generally high when -bnni option was applied and only three terminals were recovered in the conflict with the morphology-based classification in the IQ-TREE analysis shown in Figure S1. A few additional rogue taxa were identified in further analyses (Figures S3–S9). The mtDNA-based topology was highly congruent with the result of all marker analysis (Figure S8), but the partial analysis of rRNA markers did not recover some tribes monophyletic (Figure S9).

Libnetini (13 spp., one species misplaced) were found either among the deepest branches or as a sister to Dilophotini (Figure 2 and Figures S1–S7). Lyropaeini were recovered as a sister to Platerodrilini, with Antennolycini, and eventually also Leptolycini, Alyculini and Dexorini basally to them. Dictyopterini + Taphini were recovered as a monophylum (25 spp.), but Taphini (8 spp.) formed a terminal branch (Figures S1 and S2); Lycoprogentini (9 spp.) were their sister lineage or they were found in relationships with Conderini, eventually Macrolycini. Dilophotini (30 spp.) and Metriorrhynchini (161 spp.) either formed a paraphylum or a poorly supported clade (Figure 2 and Figure S1). Dihammatini represented an independent clade of 9 species and two misplaced species. Conderini (14 spp.), Thonalmini (3 spp.), Eurrhacini (8 spp.), Platerodini (48 spp.), Erotini (18 spp.), Calochromini (47 spp.), Calopterini (15 spp.), Lycini (34 sp.), Macrolycini (14 spp.), Dilophotini (30 spp.), Lyponiini (17 spp.) were well-supported and all terminals were placed in an agreement with the tribal classification, but relationships among them remains contentious. Ateliini (19 spp.) and Lyponiini (17 spp.) formed a clade with BS 100% in IQ-TREE analyses, but *Atelius* Waterhouse was recovered as a sister to Lyponiini. Further, we found repeatedly a weakly supported relationship among lineages from the Panamanian and Neotropical realms: Thonalmini, Leptolycini, Eurrhacini, Platerodini, Calopterini, and eventually Lycini. Similarly, the Oriental and Sino-Japanese Lyponiini + Ateliini, and Erotini + Slipinskiini were repeatedly recovered as putative relatives.

**Figure 3:**
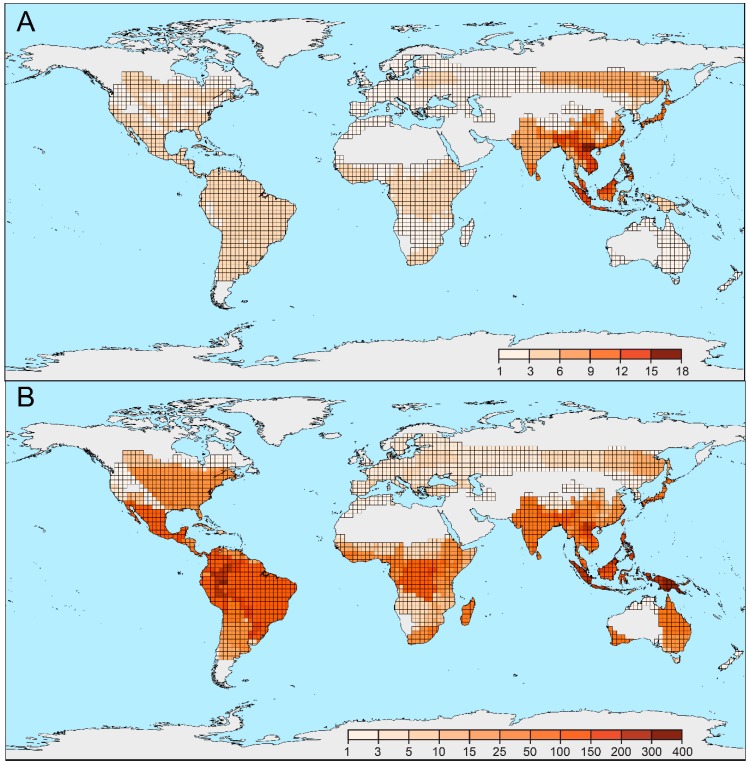
Distribution of Lycidae. (**A**) The phylogenetic diversity represented by the number of tribes displayed on the 2 degree grid. (**B**) Alpha-diversity displayed on the 2 degree grid.

The distribution data were compiled, and maps were produced, for the family and tribes (Figures S1 and S10–S29). The highest alpha-taxonomic diversity was identified in the rainforests of the Oriental realm (~1800 species; Figure 3A and Figure S1). Only ~500 species were recorded in the Afrotropical, Neotropical, and Oceanian realms. Additionally, the number of tribes was recorded in the geographic vector grid (Figure 3B). The highest number of tribes was identified in the eastern part of the Oriental realm (17 tribes), i.e., Indo-Burma, the Malay Peninsula, the islands on the Sunda Shelf, and the Philippines. Most of these tribes occur also in the Sino-Japanese realm (altogether 13 tribes), but their number decreases substantially toward high latitudes (Figure 3). Northern China and the Korean Peninsula have impoverished fauna and only the Japanese islands (12 tribes) display the phylogenetic diversity comparable to southern China. The following numbers of tribes were recorded in other realms: Palearctic (8 tribes, most of them in the coastal area of the Russian Far East), Panamanian (7 tribes in the analysis and 3 tribes recently described for a few species, Table S2), Neotropical (6 tribes), Nearctic (6 tribes), Afrotropical (6 tribes), and Oceanian (5 tribes and a single monotypic tribe with unclear relationships; Table 1 and Table S2). One or two tribes dominate in some realms: Calopterini in the Neotropical realm (282 spp., 53% of the realm’s diversity), Lycini and Metriorrhynchini in the Afrotropical realm (258 spp., 48% and 223 spp., 41%, respectively) and Metriorrhynchini in the Oceanian realm (471 spp., 73%).

### 3.3. Geographical Structure of Diversity

**Figure 4:**
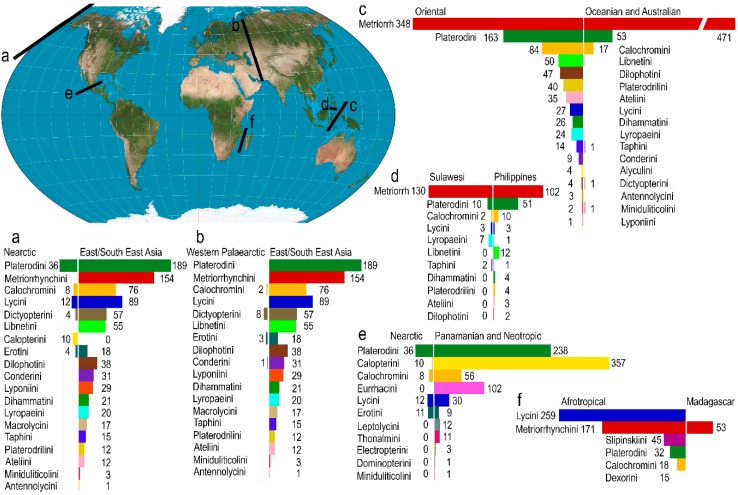
Position of zoogeographic boundaries with the high tribe-level turn-over.

The reconstruction of ancestral areas as displayed in Figures S30 and S31, the results of previous studies [10,11,18,26], and the distribution of clades endemic to a single realm (Table 1) showed that 13 of 25 tribes started their differentiation in the Oriental realm i.e., the south-eastern part of the Eurasian continent or their extant distribution is limited to this region. Lyponiini, Macrolycini, Dictyopterini and Erotini started their evolution in the Sino-Japanese realm (Table 1, Figures S30 and S31). Leptolycini and Thonalmini are endemic to the Panamanian realm; Eurrhacini and Calopterini almost completely to the Neotropical realm (with a few species colonizing the Panamanian and Nearctic realm, respectively), Dexorini and Slipinskiini to the Afrotropical realm. The reconstruction of the dispersal history of Lycini did not recover a single ancestral range due to the ambiguous deep relationships and either Nearctic/Panamanian or Afrotropical realm are putative ancestral regions. The earlier analyses identified ancestral regions for Metriorrhynchini in East Gondwana [11], the Calochromini and Dilophotini started their evolution in Indo-Burma, i.e., the northeastern part of the Oriental realm [18,26] and Ateliini in the Oriental realm [10]. Using distribution data, we defined major dispersal barriers for net-winged beetles and summarized the numbers of shared tribes and alpha taxonomic diversity (Figure 4).

## 4. Discussion

### 4.1. Higher Level Phylogeny and Classification of Net-Winged Beetles

The current phylogeny represents one of the most densely sampled family-level beetle analyses and has the balanced representation of geographical realms [5,46]. The monophyly of Lycidae usually obtained high support (Figures S1–S3). Despite the dense sampling and a relatively high number of markers, subfamily-level relationships differ among various analyses and the backbone obtained very low bootstrap support (Figure 2 and Figures S1–S7). Conversely, the monophyly of morphology-based tribes is usually robustly supported. Due to very low support and numerous conflicting topologies obtained when different outgroups, algorithms and settings were tested, the relationships, even when repeatedly recovered, must be discussed with caution.

Libnetini was commonly recovered as a deep split, sometimes as a sister or a paraphylum with Dilophotini (Figure 2) [23]. Further deep splits are represented by the clade of neotenic net-winged beetles. Two neotenics, *Dexoris* Waterhouse and *Leptolycus* Leng et Mutchler were analyzed for the first time, but their relationships with Oriental neotenics, i.e., Lyropaeinae *sensu* Bocak and Bocakova [14]), remains poorly supported (Figures S1–S3). The clade of *Leptolycus*, *Dexoris* and *Lyropaeus* Waterhouse (and related taxa) was recovered when Iberobaeniidae was used as an outgroup (BS 78%, Figure 2B), but the same analysis of the dataset with an 89-taxa outgroup recovered *Leptolycus* in relationships to Thonalmini and Eurrhacini (Figure 2B). Dexorini and Alyculini were regularly inferred as sister taxa, but with long branches and variable positions of the whole clade in alternative analyses. Lyropaeini (incl. Miniduliticolini, Platerodrilini, Alyculini, [12,13]), Dexorini (incl. Mimolibnetini; [35,47]) and Leptolycini are morphologically disparate taxa [35,44,45] and the morphological differences prompted the delimitation of several subfamilies (Figure 1C) [35]. Our analyses show that at least morphologically disparate Platerodrilini and Lyropaeini, placed recently in different subfamilies [35], are closely related and there is a possibility that a part of an observed morphological divergence is caused by ontogenetic reprograming known in other elateroid lineages [21,24,48]. Due to morphological similarity, possibly a result of miniaturization, Leptolycini have also contained Neotropical neotenics [35]. In contrast with the previous formal placement, the analyzed Caribbean *Leptolycus* represents an isolated lineage, fairly distant from Calopterini (Figures S1–S3) and an analyzed small-bodied Neotropical neotenic (an undescribed taxon provisionally designated as ‘Pseudoceratoprion’) belongs to Calopterini.

Metriorrhynchini was found as a deeply rooted isolated lineage and Dictyopterini as the next extensive clade, but Taphini was a terminal lineage in Dictyopterini (Figure 2). The current results did not provide robust support for relationships between Erotini and Dictyopterinae suggested earlier (Figure 1, [17,35,45], but not [23]). Similarly, the evidence for the close relationships of Lycoprogentini and Dictyopterini remains ambiguous (BS 79%) despite their morphological similarity [14]. Ateliini and Dilophotini (earlier placed in Ateliinae) were not recovered and the neotenic Ateliini, i.e., genera *Atelius* and *Scarelus* Waterhouse formed a clade with Lyponiini (BS 100%), but *Atelius* was a sister to *Lyponia* Waterhouse and not to *Scarelus,* are expected from the morphological evidence (Figure 2 and Figure S1). Dilophotini was recovered in a distant position, sometimes a sister to Libnetini, and the current results did not indicate any relationships to Ateliini.

The Lycini + Calopterini clade was recovered in agreement with earlier studies, although the support for their relationships remains very low (Figure 2 and Figure S1). Calopterini contains lineages with presumed neotenic females [30], but in contrast with the Oriental and Panamanian neotenics, the calopterine neotenic included in the analysis represents just a terminal subclade with a possible recent origin as has already been proposed by Miller [30] and Bocak et al. [23]. Therefore, the relationships of numerous South American neotenics should be studied in detail and their classification eventually revised. Similarly, the recently reported putative neotenic *Cautires apterus* Bocak et al. (2014) represents an independent recent shift to neoteny [49]. These findings suggest a scenario that neotenics evolve repeatedly in several elateroid lineages, including net-winged beetles as has been suggested by earlier analyses [21,22,23,24,34,48,50].

Dense sampling did not substantially improve the robustness of the net-winged beetle tree compared with the previous study [23] and only more genes can provide further information to build the natural classification based on the robustly defined natural groups. Concerning the instability of the tree, low support for most deep relationships (Figure 1), and ambiguous morphological signal [17,23,35,45], we avoid changes in the classification.

### 4.2. Diversity Centers, Ancestral Areas and Dispersal Routes

The backbone of the lycid phylogeny remains ambiguous and, therefore, the ancestral area of the whole family was not analyzed. Nevertheless, it is worth noting that the split between Lycidae and Iberobaeniidae was inferred at 125–176 mya with a delayed diversification of major net-winged beetle lineages 55–125 mya [50,51,52,53]. The endemism of Iberobaeniidae in the western Mediterranean and the distribution of numerous lycid tribes in East Asia (Figures S10–S29) point to the southern parts of Laurasia as a potential center of the early diversification.

Libnetini is restricted to the Oriental and Sino-Japanese realms with the highest species-diversity known from the eastern part of the Oriental realm. The low diversity of Libnetini has been identified in the newly accreted Indian subcontinent (Figure S11) [54]. Conversely, a relatively high diversity is known from the Sino-Japanese realm, but the diversity decreases rapidly towards the north and only a single species is recorded from Japan [55]. Their origin was inferred in the Malay Peninsula with multiple independent short distance dispersal events leading to the extant distribution (Figure S30A).

All neotenic lineages occur in humid tropics: Lyropaeini in the Oriental and southernmost part of the Sino-Japanese realms [12,56], the Dexorini in the Afrotropical realm [33,44] and Leptolycini in the Great Antilles and adjacent islands [30,35] (Figure S10). The Dexorini is a Gondwanan group and their occurrence in the Afrotropical realm can be explained only if their early origin (Figure 2A,B) and mid-Cretaceous positions of continents are considered [54,57,58]. All these lineages contain exclusively neotenics, and, due to the larviform females, a long distance over-sea dispersal has not been hypothesized in previous studies [10,12]. The origin of the Lyropaeini was inferred in the Sunda Shelf with dispersal to India and the Philippines (Figure S30B). The dispersal history of Platerodrilini was studied by Masek et al. [13] and similarly to *Lyropaeus*, the origin of the clade was inferred in the Sunda Shelf islands with subsequent dispersal to continental Asia and the Philippines. The colonization of the Philippines is old in both cases [10,13]. The climatic stability is supposedly the necessary condition for the long-term survival of these poor dispersers as these do not occur in the adjacent southern part of the Sino-Japanese realm [23,29,59].

The Dictyopterini is a species-poor lineage with the dominantly Palearctic and Sino-Japanese distribution; Taphini occur in the Oriental realm (Figures S14 and S15). Only a few Dictyopterini occur in the Nearctic realm and a single species of Taphini in the Wallacea and New Guinea. The highest diversity is present in the combined area of the eastern part of the Oriental realm and the southern part of the Sino-Japanese realm. A single Nearctic species of *Dictyoptera* Latreille in the analysis is a sister to European *Dictyoptera aurora* (Herbst). West and East Palearctic species of *Benibotarus* are closely related (Figures S1–S9) and indicate earlier connectivity between eastern and western parts of the Palearctic realm.

The Erotini is a species-poor, but widely distributed tribe (Figure S22). Their origin was inferred in Indo-Burma and they dispersed multiple times to the Sino-Japanese realm which served as a source area for the West-Palearctic fauna similarly to Dictyopterini. Only a single species was available from Northern America and its closest relative was found in Japan. The current analyses are limited, and the Nearctic realm needs to be better represented to recover the number of dispersal events across the Bering Strait and connectivity between Europe and Northern America.

Platerodini is among the most diverse tribes of Lycidae (Table 1) and the highest alpha-diversity was found in the Oriental and Neotropical realms [60]. Their origin was inferred in the Malay Peninsula (Figure S29E), but the deep topology in this clade obtained very low support and therefore any conclusion is premature. The possible relationships of Platerodini and other Neotropical and Panamanian lineages (Figure S1) point to an alternative origin in Western Gondwana. Afrotropical *Plateros* Bourgeois represent two separate terminal lineages (Figure S1).

Dihammatini and Conderini belong to tribes with the ancestral area in the Sunda Shelf (Figure S31B,C). Both dispersed to the Sino-Japanese realm and except *Xylobanellus erythropterus* (Baudi) they do not occur westward. Unlike these, Lyponiini and Macrolycini have a Sino-Japanese origin and they remained limited to this realm except a single species of *Lyponia* recorded from Northern Borneo (Figures S26 and S28) [32]. The Lycini are very diverse in the Afrotropical region (Figure S25), but the analysis placed their origin either in North America or the Afrotropical realm with low probabilities (~0.4 for both areas). The topology indicates that only two lineages, *Haplolycus* Bourgeois and *Lycus* s. str., diversified in Africa (Figure S1). The centers of origins are hypothesized in the Neotropical realm for Calopterini and Eurrhacini as these tribes occur in the north in a low number of species [61] (Figures S21 and S24).

The previously published analyses identified the origin of Calochrominae in Indo-Burma [18], Metriorrhynchini in India and Australia when these landmasses were connected and with the dispersal using drifting India as a raft when they colonized Madagascar, continental Africa and finally the Asian continent [11]. The origin of Dilophotini was inferred to Indo-Burma and subsequent dispersal events brought Dilophotini to the Sino-Japanese realm, the Greater Sundas and the Philippines [26].

Multiple tribes are limited to, or their diversification started in, the Greater Sundas, Indo-Burma or Southern China. These regions are geographically close, never isolated, and have a common tectonic history as the stable margin of Eurasia [54]. Therefore, as a working hypothesis, we propose to place the origin of net-winged beetle diversity to tropical Eurasia. Despite the amount of species represented in the dataset, further data and better supported phylogeny are needed for detailed reconstruction of ancestral areas.

### 4.3. Major Dispersal Barriers

The turn-over and representation of the major lineages are used for the definition of zoogeographical realms and vertebrates are a traditional model [46]. The recovered patterns need to be tested with other groups with different evolutionary history and dispersal ability. Hence, using poorly dispersing net-winged beetles, we discuss the effectiveness of dispersal barriers, some of them representing borders between major zoogeographic realms [46] and some specific for the studied group.

We suggest that the net-winged beetles do not cross large open sea distances. Most dispersal events were inferred across the sea straits which were, at least for some time, dry or narrow, i.e., their width was under 200 km. The poor ability of Lycidae to cross an open sea is documented by the colonization of islands. There are no net-winged beetles in New Zealand except an introduced species [15], a few species are known from Samoa and Fiji [15], one species of Cautirina on Mauritius, but no species on Reunion, a single species on Sao Tome despite only 150 km distance from the coast, no shared tribe between Florida and the Great Antilles, no net-winged beetles in the Bahamas, Jamaica, southern Lesser Antilles, the Cape Verde Archipelago, Socotra, etc. Only Fiji and Samoa species potentially dispersed over sea straits up to several hundred kilometers wide [62].

### 4.4. Mozambique Strait

The 400 km wide Mozambique Strait does not represent a serious barrier for most animals [63], but lycid faunas are substantially different in Madagascar and Africa (Figure 4). Six tribes are known from the East African coast, but no over-sea colonization is hypothesized. Only a single terminal clade of *Cautires* Waterhouse, whose origin was hypothesized in drifting India about 62 mya, is known from Madagascar [11]. Therefore, we conclude, that no net-winged beetles crossed the Mozambique Strait and none were present in Madagascar when the island separated from other Gondwanan fragments 80 mya [54] or the ancient fauna went extinct.

### 4.5. Bering Strait

The common pre-Pleistocene faunal exchanges between East Asia and North America via the Beringia Land Bridge have been documented for plants and animals in both directions from the early Paleocene until the early Pliocene [64,65,66]. Five tribes, Lycini, Calochromini, Dictyopterini, Erotini, and Platerodini contain both East Asian and North American lineages. Calochromini (*Lucaina* Dugès and *Macrolygistopterus* Pic) supposedly colonized North America from Asia [18]. The Lycini split in the Nearctic and Asian/African clades and robustly inferred sister group is crucial for an identification of their ancestral range (a putative sister-group Calopterini, BS 78% only). North American Dictyopterini were represented only by a single species which was inferred as a sister to the species from Europe (Figure S1). The sampling is sparse for a robust reconstruction in other lineages, but current results do not indicate a Quaternary faunal exchange across the Bering Strait.

### 4.6. Isthmus of Panama (Former Atrato Seaway)

The exchange between Nearctic and Neotropical realms has been identified in many animal groups and similarly, the dispersal from the Nearctic realm was identified in the Calochromini [18]. Conversely, the colonization starting from the south was inferred in the Calopterini and Eurrhacini (Figures S13 and S16). In both cases, the diversity is highly biased for the center of origin and new parts of the range were colonized by few species [61]. The analysis of Lycini did not robustly identify the ancestral region. The Nearctic and Panamanian realms samples of Lycini are limited and Neotropical representatives were unavailable.

### 4.7. Central Asia Dry Gap

The West Palearctic fauna of net-winged beetles is extremely poor when only 15 species and 5 tribes are known from Europe and the Caucasus. Conversely, the combined fauna of the Sino-Japanese and Eastern Palearctic regions represents 18 tribes and 837 species (Figure 4). Nine species from the western regions were analyzed and we found most relatives in the eastern part of Eurasia. The continental dry regions represent an impermeable barrier for lycids. The split of west Palearctic species from their closest relatives was dated to 23.6 mya for *Lygistopterus sanguineus* (L.) [18]. Such split is relatively recent and the absence of other lineages in Europe is supported by the low diversity of net-winged beetle fauna in the Baltic amber [67]. Only two genera *Lateralis* Kazantsev (Dictyopterini) and *Protolopheros* Kazantsev (Erotini), both with relatives in the Sino-Japanese realm, have been reported from the amber fossils and are not known in the extant European fauna [67,68]. The Western Palearctic fauna suffered probably from aridization and glaciation in the Pleistocene and low dispersal ability prevented re-colonization. The southern part of this barrier was supposedly crossed by *Micronychus* Motschoulsky when the Afrotropical realm was colonized from East Asia [18].

### 4.8. North Africa Dry Gap

The semi-desert areas are unsuitable for lycids and only *Lycostomus* Motschulsky (2 spp.), *Lygistopterus* Mulsant (3 spp.) and *Pyropterus* Mulsant (1 sp.) are known from the western part of the Saharo-Arabian realm and these species are related to the Palearctic fauna. The Afrotropical fauna (6 tribes, 369 spp.; Figure 3) is more diverse, especially at the species level. It contains the endemic Dexorini and Slipinskiini with the presumably ancient origin and a low number of species (15 and 46 spp.; Figure 4). The Metriorrhynchini (223 spp.) and Lycini (258 spp.) dominate in the number of species. The Metriorrhynchini (only Cautirina) colonized the Afrotropical region from drifting India ~65 mya [11]. The Calochromini (14 spp.) are hypothesized to be Asian colonists, possibly using a forest belt supposedly present in southern Asia [18,69]. Africa was isolated for a long time and no out-of-Africa dispersal event has been identified in net-winged beetles.

### 4.9. Wallace and Huxley Lines

#### 4.9.1. Borneo/Sulawesi Sector

The Wallace line lies within the Oriental realm as defined by Holt et al. [46], but we found the complete turn-over between adjacent regions at the species level and a high turn-over at the tribe level (Figure 4). The Metriorrhynchini is present on both sides of the Wallace line, but most of them belong to different subtribes with Sulawesi dominated by Australian Metriorrhynchina and the Sunda Shelf with Oriental Cautirina and Metanoeina [11]. Only a limited number of genera crossed this line in both directions (*Metriorrhynchus* and *Xylobanus*, [70,71]). Similar to Cautirina, the Calochromini (4 spp.) and Lycini (3 sp.) from Sulawesi have a high number of their closest relatives in the Oriental region (Figure 4, [18]). A low difference was found between faunas of the Moluccas and Sulawesi, although they belong to different realms proposed by Holt et al. [46].

#### 4.9.2. Sulawesi/Mindanao Sector

The stepping-stone islands connecting Northern Sulawesi and Mindanao were hypothesized as a dispersal route for *Metriorrhynchus* (~10 mya, [70]) and several shared genera in the Philippines and Sulawesi confirm the effectiveness of this connection in Metriorrhynchina (*Leptotrichalus* Kleine, *Metriorrhynchus*, *Sulabanus* Bocak and Dvorak, *Cautiromimus* Pic). Oriental *Xylobanus* Waterhouse putatively dispersed across the Makassar Strait as most species occur in Borneo and the Philippine fauna is poor [71]. We consider the connection from the Philippines to Sulawesi similarly ineffective as many Oriental tribes are missing in the Wallacea (Figure 4) [72].

#### 4.9.3. Huxley Line (the Philippines/Borneo Sector)

This line lies within the Oriental realm [46] and two potential dispersal routes can be hypothesized within this area: The connection via Palawan across the narrow but deep Balabac Strait and via the Sulu islands. The Sulu Islands were hypothesized as a dispersal route for two neotenic lineages in the Oligocene, when Palawan was much further in the north and probably submerged [10,13]. Palawan has a mixed fauna with numerous taxa having their origin in the Oriental region and Australian or Wallacean taxa being a minority. Palawan reached its position about 10 mya [73] and the Philippines were the only adjacent region from which Australian and Wallacean net-winged beetles can migrate to Palawan. The Philippine fauna is a derivation of the much more diverse fauna of New Guinea, Halmahera and Sulawesi and the Palawan fauna represents only a subset of the genera present in the Philippines. Two Oceanian genera are present in the Philippines, but missing in Borneo (*Lobatang* Bocak, *Sulabanus*, [11]); some Oceanian and Australian genera are present in the Oriental realm, but are rare and known in a few species (*Diatrichalus* Kleine, *Leptotrichalus*, *Microtrichalus* Pic, *Trichalus* Waterhouse, *Metriorrhynchus*).

### 4.10. Sea of Japan

The Japanese islands have a continental origin and although the Sea of Japan opened ~15 mya [74,75], the Japanese islands were repeatedly re-connected with the Asian continent during low sea stands [76]. Despite dry-land connection, the separation of Japanese species from their closest continental relatives was mostly hypothesized by vicariance and not by dispersal [27,28]. There is only a single species known from both regions, but the study of its identity is needed as recent studies showed a high distance-dependent differentiation within Chinese and Japanese net-winged beetles [27,28].

## 5. Conclusions

Despite the dense, worldwide sampling of net-winged beetles, the recovered deep relationships remain contentious and further information is needed for a robust classification. The Lycidae is one of major elateroid lineages and its split from the closest relative was dated in previous analyses between the mid-Jurassic (176 mya; [50]) and lower Cretaceous (125 mya; [51,52,53,77]). All studies suggest delayed start of net-winged beetle radiation either in the mid-Cretaceous (125 mya, [50]) or around the Cretaceous/Paleogene boundary (55–69 mya, [51,52,53]). The short internal branches along the backbone of our molecular topologies indicate a possible early rapid radiation of net-winged beetles.

The recovered phylogeny supports multiple origins of neoteny in Lycidae [23]. Although larviform females are known only for four genera groups (*Leptolycus*, *Lyropaeus*, *Macrolibnetis*, and *Platerodrilus*; [12,13,28,30,32,78,79]), the putative neoteny is widely accepted for further lineages: Dexorini, Ateliini, some Calopterini, *Cautires apterus* [17,33,35,44,45,59]. Some of these lineages represent deeply rooted clades, some are terminal branches within clades with fully metamorphosed females (Figure 2 and Figure S1). The recovered ranges of neotenics support the hypothesized low dispersal capacity and long-term survival only in ecologically stable habitats [23].

The early diversification is provisionally hypothesized in the southern Laurasia. The origin of 17 major lineages was inferred in this area. Two lineages, the Thonalmini and Leptolycini, are endemics of the Panamanian realm and our topologies indicate possible relationships of Eurrhacini, Platerodini, Lycini and Calopterini with these tribes. The distribution of Gondwanan lineages, i.e., Metriorrhynchini, Dexorini and Slipinskiini, needs to hypothesize an earlier origin of net-winged beetle diversification at the time when Laurasia and Gondwana were biologically interconnected [54], i.e., at least in the Early Cretaceous.

The extant distribution and phylogeny do not provide any proof for a long-distance dispersal. We identified zoogeographical boundaries which separate different net-winged beetle faunas and if they were crossed, no reversal colonization was inferred, and colonizing lineages usually remain uncommon and less diversified than autochthonous fauna (Figure 4 and Figures S10–S31). The high turn-over was identified in some areas. Unlike vertebrate fauna [46], the net-winged beetle fauna of the Oceanian origin is distributed across the Wallacea, and only the Makassar Strait represents a prominent dispersal barrier. Additionally, the net-winged beetle distribution and phylogeny suggest that the Sino-Japanese realm as proposed by Holt et al. [46] is a transitional zone [80,81]. The current results show the potential power of net-winged beetles to identify centers of origin and dispersal routes. Due to weak support for deep relationships, the centers of origin can be investigated only for tribe-level clades. More data and robust phylogeny are still needed for the robust identification of the center of origin of the whole family.

## Figures and Tables

**Table 1:** Overview of the net-winged beetle classification [14], alpha-taxonomic diversity, number of sequenced samples, distribution and inferred ancestral areas. The recently described tribe rank taxa unavailable for analysis are listed in Table S2. Note: n = the neotenic lineage with proved or supposed larviform females at least in some species. Area codes: 1 Palearctic realm (PAL), western part; 2 Palearctic/Sino-Japanese realm (SIN-JAP) China, Korea, Taiwan; 3 SIN-JAP—Japan; 4 Oriental realm (OR), the Philippines; 5 OR, Indo-Burma; 6 OR, Malay Peninsula; 7 OR, Sunda Islands; 8 Oceanian (OC) realm, Sulawesi, Moluccas, 9 OC, New Guinea; 10 Australian realm; 11 OR, India, 12 Afrotropical (AFR) realm, the continental part; 13 AFR, Madagascar; 14 Nearctic realm; 15 Panamanian realm, Caribbean; 16 Neotropical realm, the continental part.

Subfamily	Tribe	No of Species	Distribution		Hypothesized Ancestral Area
		Described/Sequenced			
LIBNETINAE	Libnetini	112	17	2–7, 11	Oriental (Figure S30A)
LEPTOLYCINAE	Leptolycini ^n^	12	4	15	Panamanian (endemic)
DEXORINAE	Dexorini ^n^	15	2	12	Afrotropical (endemic)
LYROPAEINAE	Lyropaeini ^n^	43	9	4–8, 11	Oriental (Figure S30B)
	Alyculini ^n^	3	1	6–7	Oriental (endemic)
	Antennolycini ^n^	3	2	6	Oriental (endemic)
	Platerodrilini ^n^	49	32	2, 4–7	Oriental (endemic)
DICTYOPTERINAE	Dictyopterini	73	25	1–7, 14	Sino-Japanese (Figure S30C)
	Lycoprogenthini	7	9	2, 4–7	Oriental (endemic)
	Taphini	31	9	2, 4–10	Oriental (endemic except one sp.)
LYCINAE	Ateliini ^n^	45	19	2, 4–7	Oriental (endemic [10])
	Metriorrhynchini ^n^	1403	161	2–13	Australian [11]
	Dilophotini	81	30	2–7	Oriental [26]
	Calochromini	288	47	1–12, 14–16	Oriental [18]
	Calopterini ^n^	367	15	14–16	Neotropical (endemic, a few species in the Nearctic realm)
	Conderini	42	14	1–7	Oriental (Figure S31C)
	Dihammatini	44	9	2–7	Oriental (Figure S31B)
	Erotini	54	18	1–3, 14	Sino-Japanese (Figure S31A)
	Slipinskiini	46	4	12	Afrotropical (endemic)
	Eurrhacini	102	8	15–16	Neotropical (endemic)
	Lycini	413	34	1–8, 12, 14–16	unresolved: Nearctic or Afrotropical
	Lyponiini	45	17	2–3, 5, 7, 11	Sino-Japanese
	Macrolycini	69	14	2–3, 5, 11	Sino-Japanese
	Platerodini	861	48	1–12, 14–15	Oriental (Figure S30E)
	Thonalmini	11	3	15	Panamanian (endemic)
LYCIDAE		4230	551	1–16	

## Supplementary Materials

The following are available online at http://www.mdpi.com/2075-4450/9/4/154/s1, Table S1: The list of sequenced samples, Table S2: Tribe-level taxa not included in the molecular analysis, Table S3: Primers used for PCR amplification, Table S4: Datasets and models, Figure S1: The IQ-tree topology inferred from the MAFFT alignment of all markers and 89 taxa as an outgroup, Figure S2: As Figure S1, but all taxa represented, full resolution tree of net-winged beetles, Figure S3: The IQ-tree topology inferred from the MAFFT alignment of all markers and *Iberobaenia* as a single outgroup, Figure S4: The IQ-tree topology inferred from the MAFFT alignment of all markers and 89 taxa as an outgroup with the -bnni option applied, Figure S5: The IQ-tree topology inferred from the MAFFT alignment of all markers and *Iberobaenia* as a single outgroup with the -bnni option applied, Figure S6: The RAXML topology inferred from the MAFFT alignment of all markers and 89 taxa as an outgroup, Figure S7: The RAxML topology inferred from the MAFFT alignment of all markers and *Iberobaenia* as a single outgroup, Figure S8: The IQ-tree topology inferred from the MAFFT alignment of rRNA markers and 89 taxa as an outgroup with the -bnni option applied, Figure S9. The IQ-tree topology inferred from the MAFFT alignment of mtDNA markers and 89 taxa as an outgroup with the -bnni option applied, Figure S10 Distribution and species diversity of the tribes Leptolycini, Dexorini, and Lyropaeini, Figure S11: Ditto Libnetini, Figure S12: Ditto Lyropaeini, Figure S13: Ditto Platerodrilini, Figure S14: Ditto Dictyopterini, Figure S15: Ditto Taphini. Figure S16: Ditto Metriorrhynchini, Figure S17: Ditto Slipinskiini, Figure S18: Ditto Dihammatini, Figure S19: Ditto Platerodini, Figure S20: Ditto Conderini, Figure S21: Ditto Eurrhacini, Figure S22: Ditto Erotini, Figure S23: Ditto Calochromini, Figure S24: Ditto Calopterini, Figure S25: Ditto Lycini, Figure S26: Ditto Macrolycini, Figure S27: Ditto Dilophotini, Figure S28: Ditto Lyponiini, Figure S29: Ditto Ateliini, Figure S30: Identification of ancestral areas A—Libnetini, B—Lyropaeini, C—Dictyopterini, E—Platerodini, Figure S31: Ditto A—Erotini, B—Dihammatini, C– Conderini, D—Lyponiini, E—Macrolycini.
