# Supplementary material for: Molecular Phylogeny, Diversity and Zoogeography of Net-Winged Beetles (Coleoptera: Lycidae)
Source: Insects. 2018 Nov 1;9(4):154. doi: 10.3390/insects9040154 (PMC6315567; doi:10.3390/insects9040154)
Supplement: Supplementary file 1 [file insects-09-00154-s001.pdf]

Molecular phylogeny, diversity and zoogeography of net-winged beetles (Coleoptera: Lycidae)

Michal Masek, Michal Motyka, Dominik Kusy, Matej Bocek, Yun Li, Ladislav Bocak

Supporting information

Table S1. The list of sequenced samples.
Table S2. Tribe-level taxa not included in the molecular analysis.
Table S3. Primers used for PCR amplification.
Table S4. Datasets and models.
Figure S1. The IQ-tree topology inferred from the MAFFT alignment of all markers and 89 taxa as an outgroup. Species level terminal collapsed, miniatures show general appearance of typical representatives.
Figure S2. As Figure S1, but all taxa represented, full resolution tree of net-winged beetles.
Figure S3. The IQ-tree topology inferred from the MAFFT alignment of all markers and Iberobaenia as a single outgroup.
Figure S4. The IQ-tree topology inferred from the MAFFT alignment of all markers and 89 taxa as an outgroup with the -bnni option applied. All taxa represented, full resolution tree of net-winged beetles.
Figure S5. The IQ-tree topology inferred from the MAFFT alignment of all markers and *Iberobaenia* as a single outgroup with the -bnni option applied.
Figure S6. The RAXML topology inferred from the MAFFT alignment of all markers and 89 taxa as an outgroup. All taxa represented, full resolution tree of net-winged beetles.
Figure S7. The RAxML topology inferred from the MAFFT alignment of all markers and *Iberobaenia* as a single outgroup.
Figure S8. The IQ-tree topology inferred from the MAFFT alignment of rRNA markers and 89 taxa as an outgroup with the -bnni option applied.
Figure S9. The IQ-tree topology inferred from the MAFFT alignment of mtDNA markers and 89 taxa as an outgroup with the -bnni option applied.
Figure S10 Distribution and species diversity of the tribes Leptolycini, Dexorini, and Lyropaeini.
Figure S11. Distribution and species diversity of the subfamily Libnetinae.
Figure S12. Distribution and species diversity of the tribe Lyropaeini.
Figure S13. Distribution and species diversity of the tribe Platerodrilini.
Figure S14. Distribution and species diversity of the tribe Dictyopterini.
Figure S15. Distribution and species diversity of the tribe Taphini.
Figure S16. Distribution and species diversity of the tribe Metriorrhynchini.
Figure S17. Distribution and species diversity of the tribe Slipinskiini.
Figure S18. Distribution and species diversity of the tribe Dihammatini.
Figure S19. Distribution and species diversity of the tribe Platerodini.
Figure S20. Distribution and species diversity of the tribe Conderini.
Figure S21. Distribution and species diversity of the tribe Eurrhacini..

Figure S22. Distribution and species diversity of the tribe Erotini.
Figure S23. Distribution and species diversity of the tribe Calochromini.
Figure S24. Distribution and species diversity of the tribe Calopterini.
Figure S25. Distribution and species diversity of the tribe Lycini.
Figure S26. Distribution and species diversity of the tribe Macrolycini.
Figure S27. Distribution and species diversity of the tribe Dilophotini.
Figure S28. Distribution and species diversity of the tribe Lyponiini
Figure S29. Distribution and species diversity of the tribe Ateliini.
Figure S30. Identification of ancestral areas A – Libnetini, B – Lyropaeini, C – Dictyopterini, E – Platerodini.
Figure S31. Identification of ancestral areas A – Erotini, B – Dihammatini, C– Conderini, D – Lyponiini, E – Macrolycini.

**Lycidae (Ingroup)**								
Alyculini	*Alyculus kurbatovi*	UPOL 000543	Java	DQ181072	DQ181146	DQ180998	DQ181220	DQ181374
Antennolycini	*Antenolycus constrictus*	UPOL 000L22*	Malaysia	DQ181051	DQ181125	DQ180977	DQ181199	DQ181353
Antennolycini	*Microlyropaeus dembickyi*	UPOL 000542	Sumatra	DQ181071	DQ181145	DQ180997	DQ181219	DQ181373
Ateliini	*Atelius* sp.	UPOL VK0733	China	–	–	KT752146	KT751822	KT751975
Ateliini	*Scarelus anthracinus*	UPOL VM0006	Malaysia	HM451125	HM451083	HM451002	HM451042	HM451211
Ateliini	*Scarelus baranciki*	UPOL VM0019	Borneo	HM451137	HM451093	HM451012	HM451051	–
Ateliini	*Scarelus brastagiensis*	UPOL VM0012	Sumatra	HM451131	HM451088	HM451006	HM451047	HM451217
Ateliini	*Scarelus cibodasensis*	UPOL VM0007	Java	HM451126	HM451084	HM451003	HM451043	HM451212
Ateliini	*Scarelus crudus*	UPOL VM0049	Philippines	HM451165	HM451118	HM451035	HM451076	HM451248
Ateliini	*Scarelus emasensis*	UPOL VM0022	Borneo	HM451140	–	HM451015	HM451054	HM451223
Ateliini	*Scarelus flavicolis*	UPOL VM0031	Sumatra	HM451149	HM451104	HM451021	HM451061	HM451232
Ateliini	*Scarelus loksadoensis*	UPOL VM0014	Borneo	HM451133	–	HM451008	HM451049	–
Ateliini	*Scarelus longicornis*	UPOL VM0026	Sumatra	HM451144	HM451099	HM451018	HM451057	HM451227
Ateliini	*Scarelus nigricornis*	UPOL VM0021	Borneo	HM451139	HM451095	HM451014	HM451053	HM451222
Ateliini	*Scarelus pahangensis*	UPOL VM0004	Malaysia	HM451123	HM451081	HM451000	HM451040	HM451209
Ateliini	*Scarelus pseudoumbosus*	UPOL VM0002	Malaysia	HM451121	HM451079	HM450999	HM451038	HM451207
Ateliini	*Scarelus ruficolis*	UPOL VM0033	Sumatra	HM451151	HM451106	HM451023	HM451063	HM451234
Ateliini	*Scarelus rufus*	UPOL VM0034	Sumatra	HM451152	HM451107	HM451024	HM451064	HM451235
Ateliini	*Scarelus salvani*	UPOL VM0050	Philippines	HM451166	HM451119	HM451036	HM451077	HM451249
Ateliini	*Scarelus sanguineus*	UPOL VM0023	Sumatra	HM451141	HM451096	HM451016	HM451055	HM451224
Ateliini	*Scarelus saranganensis*	UPOL VM0011	Java	HM451130	HM451087	HM451005	HM451046	HM451216
Ateliini	*Scarelus similis*	UPOL VM0005	Borneo	HM451124	HM451082	HM451001	HM451041	HM451210
Ateliini	*Scarelus* sp.	UPOL 000582	Borneo	DQ181085	DQ181159	DQ181011	DQ181233	DQ181387
Ateliini	*Scarelus* sp.	UPOL 000583	Borneo	DQ181086	DQ181160	DQ181012	DQ181234	DQ181388
Ateliini	*Scarelus* sp.	UPOL 000L15	Borneo	DQ181046	DQ181120	DQ180972	DQ181194	DQ181348
Calochromini	*Micronychus* sp.	UPOL MT0019	RSA	KU496010	KU496051	KU495971	KU496109	KU496202
Calochromini	*Micronychus* sp.	UPOL MT0020	Zambia	–	–	KU495973	KU496101	KU496205
Calochromini	*Micronychus* sp.	UPOL MT0021	Zambia	–	–	KU495974	KU496102	KU496170
Calochromini	*Micronychus* sp.	UPOL MT0022	RSA	–	–	KU495977	KU496115	KU496206
Calochromini	*Micronychus* sp.	UPOL MT0023	Kenya	–	–	KU495972	KU496116	KU496171
Calochromini	*Micronychus* sp.	UPOL MT0027	Kenya	–	–	KU495975	KU496110	KU496204
Calochromini	*Micronychus* sp.	UPOL MT0028	RSA	–	–	–	KU496144	–
Calochromini	*Micronychus* sp.	UPOL MT0029	RSA	–	–	–	KU496140	KU496207
Calochromini	*Micronychus* sp.	UPOL MT0033	Kenya	–	–	KU495976	KU496111	KU496203
Calochromini	*Micronychus* sp.	UPOL MT0060	RSA	KU496011	KU496052	–	KU496142	KU496201
Calochromini	*Calochromus* sp.	UPOL A00477	Malaysia	KT752159	KT752318	KT751987	KT751669	KT751829
Calochromini	*Calochromus* sp.	UPOL A00617	Laos	KT752282	KT752446	KT752115	KT751789	KT751948
Calochromini	*Calochromus* sp.	UPOL 000033	Borneo	DQ181060	DQ181134	DQ180986	DQ181208	DQ181362
Calochromini	*Calochromus* sp.	UPOL 000124	Borneo	DQ181061	DQ181135	DQ180987	DQ181209	DQ181363
Calochromini	*Calochromus* sp.	UPOL 000347	Borneo	DQ181068	DQ181142	DQ180994	DQ181216	DQ181370
Calochromini	*Calochromus* sp.	UPOL 000L16	China	DQ181047	DQ181121	DQ180973	DQ181195	DQ181349
Calochromini	*Calochromus* sp.	UPOL MT0001	China	KU496008	KU496036	KU495936	KU496068	KU496184
Calochromini	*Calochromus* sp.	UPOL MT0002	Borneo	KU496017	KU496038	KU495939	KU496070	KU496152
Calochromini	*Calochromus* sp.	UPOL MT0003	Laos	–	–	KU495998	KU496137	KU496196
Calochromini	*Calochromus* sp.	UPOL MT0004	Malaysia	KU496032	KU496060	KU495992	KU496122	KU496187
Calochromini	*Calochromus* sp.	UPOL MT0005	Sumatra	–	–	KU495942	KU496073	KU496155
Calochromini	*Calochromus* sp.	UPOL MT0006	China	–	–	KU495957	KU496079	KU496174
Calochromini	*Calochromus* sp.	UPOL MT0007	Borneo	–	–	KU495940	KU496071	KU496153
Calochromini	*Calochromus* sp.	UPOL MT0008	Laos	–	–	–	KU496118	KU496163
Calochromini	*Calochromus* sp.	UPOL MT0009	India	–	–	KU495960	–	KU496175
Calochromini	*Calochromus* sp.	UPOL MT0010	Malaysia	–	–	KU495994	KU496123	KU496188
Calochromini	*Calochromus* sp.	UPOL MT0011	Malaysia	–	–	KU495938	KU496100	KU496186
Calochromini	*Calochromus* sp.	UPOL MT0012	Borneo	KU496024	KU496045	KU495952	KU496082	KU496148
Calochromini	*Calochromus* sp.	UPOL MT0013	India	–	–	KU495937	KU496069	KU496185
Calochromini	*Calochromus* sp.	UPOL MT0014	Malaysia	KU496009	KU496037	KU495941	KU496072	KU496154
Calochromini	*Calochromus* sp.	UPOL MT0015	India	–	–	–	KU496086	KU496164
Calochromini	*Calochromus* sp.	UPOL MT0016	India	–	–	–	KU496087	KU496166
Calochromini	*Calochromus* sp.	UPOL MT0017	India	KU496014	KU496049	KU495968	KU496112	KU496172
Calochromini	*Calochromus* sp.	UPOL MT0025	Papua New Guinea	KU496035	KU496067	–	KU496129	KU496189
Calochromini	*Calochromus* sp.	UPOL MT0026	India	KU496012	KU496046	KU495963	–	–
Calochromini	*Calochromus* sp.	UPOL MT0031	India	–	–	–	KU496088	KU496165
Calochromini	*Calochromus* sp.	UPOL MT0032	Sumatra	KU496027	KU496063	KU496001	KU496130	KU496190
Calochromini	*Calochromus* sp.	UPOL MT0034	Laos	–	–	KU495953	KU496083	KU496149
Calochromini	*Calochromus* sp.	UPOL MT0035	Sumatra	–	–	KU495985	KU496105	KU496211
Calochromini	*Calochromus* sp.	UPOL MT0036	Laos	–	–	KU495999	KU496138	KU496195
Calochromini	*Calochromus* sp.	UPOL MT0037	Malaysia	–	–	KU495948	KU496078	KU496161
Calochromini	*Calochromus* sp.	UPOL MT0038	China	–	–	KU495980	–	–
Calochromini	*Calochromus* sp.	UPOL MT0039	Cambodia	–	–	KU495995	KU496125	
Calochromini	*Calochromus* sp.	UPOL MT0040	Malaysia	–	–	KU495954	KU496084	KU496150
Calochromini	*Calochromus* sp.	UPOL MT0041	Borneo	–	–	KU496002	KU496134	KU496192
Calochromini	*Calochromus* sp.	UPOL MT0043	Malaysia	–	–	KU495955	KU496085	KU496151
Calochromini	*Calochromus* sp.	UPOL MT0044	Borneo	–	–	KU496003	KU496135	KU496193
Calochromini	*Calochromus* sp.	UPOL MT0045	Borneo	–	–	KU496004	KU496136	KU496194
Calochromini	*Calochromus* sp.	UPOL MT0046	Laos	–	–	KU496005	KU496131	–
Calochromini	*Calochromus* sp.	UPOL MT0047	Laos	KU496029	KU496065	KU496000	KU496139	KU496197
Calochromini	*Calochromus* sp.	UPOL MT0048	China	–	–	KU495986	KU496104	KU496210
Calochromini	*Calochromus* sp.	UPOL MT0049	Malaysia	KU496018	KU496039	KU495945	KU496075	KU496158
Calochromini	*Calochromus* sp.	UPOL MT0050	Laos	–	–	KU495949	KU496106	KU496146
Calochromini	*Calochromus* sp.	UPOL MT0051	Laos	KU496020	KU496040	KU495946	KU496077	KU496160
Calochromini	*Calochromus* sp.	UPOL MT0052	Laos	–	–	KU495991	KU496090	KU496169
Calochromini	*Calochromus* sp.	UPOL MT0053	China	–	–	KU495958	KU496080	–
Calochromini	*Calochromus* sp.	UPOL MT0054	Laos	KU496028	KU496064	KU496006	KU496132	–
Calochromini	*Calochromus* sp.	UPOL MT0055	Malaysia	–	–	KU495950	KU496107	KU496147
Calochromini	*Calochromus* sp.	UPOL MT0056	China	–	–	KU495964	KU496094	–
Calochromini	*Calochromus* sp.	UPOL MT0057	Malaysia	KU496019	KU496041	KU495947	KU496076	–
Calochromini	*Calochromus* sp.	UPOL MT0058	Cambodia	–	–	KU496007	KU496133	KU496191
Calochromini	*Calochromus* sp.	UPOL MT0059	Malaysia	KU496021	KU496044	KU495943	KU496141	KU496156
Calochromini	*Calochromus* sp.	UPOL MT0061	China	–	–	KU495988	KU496143	KU496200
Calochromini	*Calochromus* sp.	UPOL MT0062	India	KU496013	KU496048	KU495961	KU496092	KU496176
Calochromini	*Calochromus* sp.	UPOL MT0063	India	–	–	KU495962	KU496093	KU496177
Calochromini	*Calochromus* sp.	UPOL MT0064	India	KU496023	KU496042	–	KU496089	KU496167
Calochromini	*Calochromus* sp.	UPOL MT0065	India	–	–	KU495966	KU496091	KU496178
Calochromini	*Calochromus* sp.	UPOL MT0066	India	–	–	KU495967	KU496119	KU496209
Calochromini	*Calochromus* sp.	UPOL MT0067	India	KU496016	KU496050	KU495969	KU496113	KU496208
Calochromini	*Calochromus* sp.	UPOL MT0068	Malaysia	KU496022	KU496043	KU495944	KU496074	KU496157
Calochromini	*Calochromus* sp.	UPOL MT0069	Malaysia	KU496033	KU496061	KU495993	KU496124	–
Calochromini	*Calochromus* sp.	UPOL MT0070	Laos	KU496030	KU496058	KU495951	KU496108	KU496145
Calochromini	*Calochromus* sp.	UPOL MT0071	India	–	–	KU495956	KU496103	KU496168
Calochromini	*Calochromus* sp.	UPOL MT0072	India	–	–	KU495970	KU496114	KU496173
Calochromini	*Calochromus* sp.	UPOL MT0074	California	KU496026	KU496066	KU495978	KU496121	KU496183

Calochromini	*Calochromus* sp.	UPOL MT0075	China	–	–	KU495989	KU496128	KU496199
Calochromini	*Calochromus* sp.	UPOL MT0076	China	KU496034	KU496062	KU495996	KU496126	–
Calochromini	*Calochromus* sp.	UPOL MT0077	China	KU496015	KU496047	KU495959	KU496081	KU496214
Calochromini	*Calochromus* sp.	UPOL MT0078	China	–	–	KU495965	–	KU496213
Calochromini	*Calochromus* sp.	UPOL MT0079	Laos	–	–	KU495997	KU496127	–
Calochromini	*Calochromus* sp.	UPOL MT0080	China	–	–	KU495990	–	KU496198
Calochromini	*Lygistopterus* sp.	UPOL MT0030	Greece	KU496025	KU496053	KU495979	KU496120	KU496182
Calochromini	*Macrolygistopterus* sp.	UPOL MT0073	Ecuador	KU496031	KU496059	KU495987	KU496099	KU496215
Calopterini	*Calopteron* sp.	UPOL A00621	Panama	KT752284	KT752449	KT752118	KT751792	KT751951
Calopterini	*Calopteron* sp.	UPOL A00628	Ecuador	–	–	KT752125	KT751798	KT751957
Calopterini	*Calopteron* sp.	UPOL A00629	Ecuador	–	–	KT752126	KT751799	KT751958
Calopterini	*Calopteron* sp.	UPOL A00630	Nicaragua	KT752289	KT752454	KT752127	KT751800	KT751959
Calopterini	*Calopteron* sp.	UPOL A00631	Ecuador	–	–	KT752128	KT751801	KT751960
Calopterini	*Calopteron* sp.	UPOL A00633	Ecuador	KT752291	KT752456	KT752129	KT751803	KT751962
Calopterini	*Calopteron* sp.	UPOL A00637	Argentina	–	–	KT752133	KT751807	–
Calopterini	*Calopteron* sp.	UPOL A00640	Nicaragua	KT752295	KT752460	KT752136	KT751809	KT751967
Calopterini	*Calopteron* sp.	UPOL A00645	Nicaragua	KT752296	KT752462	KT752139	KT751812	KT751970
Calopterini	*Calopteron* sp.	UPOL A00647	Bolivia	KT752298	KT752463	KT752141	KT751814	–
Calopterini	*Calopteron* sp.	UPOL A00650	Bolivia	KT752301	KT752466	KT752144	KT751817	KT751973
Calopterini	*Calopteron* sp.	UPOL A00651	Ecuador	–	–	KT752145	KT751818	KT751974
Calopterini	*Calopteron* sp.	UPOL 000L25	Ecuador	DQ181053	DQ181127	DQ180979	DQ181201	DQ181355
Calopterini	*Idiopteron biplagiatum*	UPOL 000M44	Ecuador	DQ181057	DQ181131	DQ180983	DQ181205	DQ181359
Calopterini	*Metapteron* sp.	UPOL IR2002	Peru	AF451946	DQ198757	–	DQ198588	–
Conderini	*Conderis rufohumeralis*	UPOL 000581	Japan	DQ181084	DQ181158	DQ181010	DQ181232	DQ181386
Conderini	*Conderis signicollis*	UPOL 000194	Malaysia	DQ181062	DQ181136	DQ180988	DQ181210	DQ181364
Conderini	*Conderis* sp.	UPOL A00601	Laos	KT752267	KT752431	KT752099	KT751774	KT751933
Conderini	*Conderis* sp.	UPOL A00602	China	KT752268	KT752432	KT752100	KT751775	KT751934
Conderini	*Conderis* sp.	UPOL A00603	China	KT752269	KT752433	KT752101	KT751776	KT751935
Conderini	*Conderis* sp.	UPOL A00605	Vietnam	KT752271	KT752435	KT752103	KT751778	KT751937
Conderini	*Conderis* sp.	UPOL A00606	India	KT752272	KT752436	KT752104	KT751779	KT751938
Conderini	*Conderis* sp.	UPOL A00607	China	KT752273	KT752437	KT752105	KT751780	KT751939
Conderini	*Conderis* sp.	UPOL A00609	Japan	KT752275	KT752439	KT752107	KT751782	KT751941
Conderini	*Conderis* sp.	UPOL A00610	Cambodia	KT752276	KT752440	KT752108	KT751783	KT751942
Conderini	*Conderis* sp.	UPOL A00612	India	KT752277	KT752441	KT752110	KT751784	KT751943
Conderini	*Conderis* sp.	UPOL A00613	Borneo	KT752278	KT752442	KT752111	KT751785	KT751944
Conderini	*Conderis* sp.	UPOL A00614	Malaysia	KT752279	KT752443	KT752112	KT751786	KT751945
Conderini	*Xylobanellus* sp.	UPOL A00604	China	KT752270	KT752434	KT752102	KT751777	KT751936
Conderini	*Xylobanellus* sp.	UPOL A00608	Japan	KT752274	KT752438	KT752106	KT751781	KT751940
Conderini	*Xylobanellus* sp.	UPOL A00611	China	–	–	KT752109	–	–
Conderini	*Xylobanellus* sp.	UPOL A00615	Laos	KT752280	KT752444	KT752113	KT751787	KT751946
Conderini	*Xylobanellus* sp.	UPOL A00616	Laos	KT752281	KT752445	KT752114	KT751788	KT751947
Dexorinae	*Dexoris chome*	UPOL A00654	Tanzania	KT752302	KT752467	KT752148	KT751819	–
Dexorinae	*Dexoris* sp.	UPOL VP0045	Cameroon	KT752309	KT752468	KT752147	KT751823	–
Dictyopterini	*Benibotarus nigripennis*	UPOL 000572	Japan	DQ181075	DQ181149	DQ181001	DQ181223	DQ181377
Dictyopterini	*Benibotarus spinicoxis*	UPOL 000573	Japan	DQ181076	DQ181150	DQ181002	DQ181224	DQ181378
Dictyopterini	*Benibotarus spinicoxis*	UPOL A00569	China	KT752235	KT752400	KT752067	KT751745	KT751902
Dictyopterini	*Benibotarus taygetanus*	UPOL A00568	Hungary	KT752234	KT752399	KT752066	KT751744	KT751901
Dictyopterini	*Benibotarus taygetanus*	UPOL 001285	Czech Republic	KF625694	KF626280	KF625983	KF625392	–
Dictyopterini	*Dictyoptera aurora*	UPOL 001276	Czech Republic	KF625687	KF626273	KF625977	KF625386	–
Dictyopterini	*Dictyoptera elegans*	UPOL 000570	Japan	DQ181073	DQ181147	DQ180999	DQ181221	DQ181375
Dictyopterini	*Dictyoptera* sp.	UPOL 001275	USA	KF625686	KF626272	KF625976	KF625385	–
Dictyopterini	*Dictyoptera speciosa*	UPOL 000571	Japan	DQ181074	DQ181148	DQ181000	DQ181222	DQ181376
Dictyopterini	*Dictyoptera* sp.	UPOL A00540	Sumatra	KT752209	KT752371	KT752038	–	–
Dictyopterini	Dictyopterini gen.sp.	UPOL ZL2013	Japan	–	–	EF143223	EF143238	EF143252
Dictyopterini	Dictyopterini indet.	UPOL A00519	China	KT752191	KT752352	KT752022	KT751703	–
Dictyopterini	Dictyopterini indet.	UPOL A00520	Malaysia	KT752192	KT752353	KT752023	KT751704	KT751862
Dictyopterini	Dictyopterini indet.	UPOL A00534	Malaysia	–	KT752365	KT752033	KT751716	KT751871
Dictyopterini	Dictyopterini indet.	UPOL A00597	Malaysia	KT752263	KT752427	KT752095	KT751771	KT751929
Dictyopterini	Dictyopterini indet.	UPOL 001283	Japan	KF625692	KF626278	KF625982	KF625391	–
Dictyopterini	Dictyopterini indet.	UPOL 001278	Malaysia	KF625689	KF626275	KF625979	KF625388	–
Dictyopterini	Dictyopterini indet.	UPOL 001282	Japan	KF625691	KF626277	KF625981	KF625390	–
Dictyopterini	*Helcophorus* sp.	UPOL A00570	Thailand	KT752236	KT752401	KT752068	–	KT751903
Dictyopterini	*Helcophorus* sp.	UPOL A00618	China	KT752283	KT752447	KT752116	KT751790	KT751949
Dictyopterini	*Helcophorus* sp.	UPOL 001369	China	KF625699	KF626285	KF625988	KF625397	–
Dictyopterini	*Pyropterus nigroruber*	UPOL 000574	Japan	DQ181077	DQ181151	DQ181003	DQ181225	DQ181379
Dictyopterini	*Pyropterus nigroruber*	UPOL 001277	Czech Republic	KF625688	KF626274	KF625978	KF625387	–
Dihammatini	*Dihammatus* sp.	UPOL A00546	China	KT752215	KT752377	KT752044	KT751724	KT751881
Dihammatini	*Dihammatus* sp.	UPOL A00594	Malaysia	KT752260	KT752424	KT752092	–	KT751926
Dihammatini	*Dihammatus* sp.	UPOL A00596	Sumatra	KT752262	KT752426	KT752094	KT751770	KT751928
Dihammatini	*Dihammatus* sp.	UPOL A00598	Malaysia	KT752264	KT752428	KT752096	–	KT751930
Dihammatini	*Dihammatus* sp.	UPOL A00599	China	KT752265	KT752429	KT752097	KT751772	KT751931
Dihammatini	*Dihammatus* sp.	UPOL A00600	Laos	KT752266	KT752430	KT752098	KT751773	KT751932
Dihammatini	*Dihammatus* sp.	UPOL A00625	Malaysia	KT752287	KT752452	KT752122	KT751796	KT751954
Dihammatini	*Dihammatus* sp.	UPOL 000L12	Borneo	DQ181043	DQ181117	DQ180969	DQ181191	DQ181345
Dihammatini	*Dihammatus* sp.	UPOL 001001	Sumatra	DQ181103	DQ181177	DQ181029	DQ181251	DQ181405
Dihammatini	*Dihammatus* sp.	UPOL 001009	Sumatra	DQ181106	DQ181180	DQ181032	DQ181254	DQ181408
Dihammatini	*Dihammatus* sp.	UPOL 001017	Sumatra	DQ181108	DQ181182	DQ181034	DQ181256	DQ181410
Dilophotini	*Dilophotes* sp.	UPOL 000244	Borneo	DQ181066	DQ181140	DQ180992	DQ181214	DQ181368
Dilophotini	*Dilophotes* sp.	UPOL TH0046	Laos	–	–	KJ404937	KJ405138	–
Dilophotini	*Dilophotes* sp.	UPOL TH0068	Malaysia	–	–	KJ404957	KJ405156	KJ405307
Dilophotini	*Dilophotes* sp.	UPOL TH0078	Laos	–	–	KJ404967	KJ405166	KJ405316
Dilophotini	*Dilophotes* sp.	UPOL TH0041	Sumatra	–	–	KJ404933	KJ405133	–
Dilophotini	*Dilophotes* sp.	UPOL TH0079	Laos	–	–	KJ404968	KJ405167	KJ405317
Dilophotini	*Dilophotes* sp.	UPOL TH0080	Laos	–	–	KJ404969	KJ405168	KJ405318
Dilophotini	*Dilophotes* sp.	UPOL TH0043	Sumatra	–	–	KJ404934	KJ405135	–
Dilophotini	*Dilophotes* sp.	UPOL TH0007	Sumatra	–	–	KJ404908	KJ405101	KJ405284
Dilophotini	*Dilophotes* sp.	UPOL A00060	Laos	KC538072	KC537863	KC538740	KC538359	KC538552
Dilophotini	*Dilophotes* sp.	UPOL TH0190	China	–	–	KJ405078	KJ405270	KJ405398
Dilophotini	*Dilophotes* sp.	UPOL TH0006	Sumatra	–	–	KJ404907	KJ405100	KJ405283
Dilophotini	*Dilophotes* sp.	UPOL TH0023	Sumatra	–	–	KJ404923	KJ405115	KJ405294
Dilophotini	*Dilophotes* sp.	UPOL TH0020	Sumatra	–	–	KJ404920	KJ405112	KJ405292
Dilophotini	*Dilophotes* sp.	UPOL TH0081	Laos	–	–	KJ404970	KJ405169	KJ405319
Dilophotini	*Dilophotes* sp.	UPOL TH0095	Laos	–	–	KJ404984	KJ405179	KJ405333
Dilophotini	*Dilophotes* sp.	UPOL TH0093	Borneo	–	–	KJ404982	KJ405177	KJ405331
Dilophotini	*Dilophotes* sp.	UPOL TH0101	Borneo	–	–	KJ404990	KJ405185	KJ405339
Dilophotini	*Dilophotes* sp.	UPOL TH0160	Malaysia	–	–	KJ405048	KJ405242	KJ405368
Dilophotini	*Dilophotes* sp.	UPOL TH0131	China	–	–	KJ405020	KJ405214	–
Dilophotini	*Dilophotes* sp.	UPOL TH0152	Malaysia	–	–	KJ405040	KJ405234	KJ405365
Dilophotini	*Dilophotes* sp.	UPOL TH0059	Sumatra	–	–	KJ404950	KJ405151	–
Dilophotini	*Dilophotes* sp.	UPOL TH0039	Java	–	–	KJ404931	KJ405131	–
Dilophotini	*Dilophotes* sp.	UPOL TH0069	Malaysia	–	–	KJ404958	KJ405157	–

Dilophotini	*Dilophotes* sp.	UPOL A00003	Sumatra	–	–	KJ404888	KJ405081	KJ405272
Dilophotini	*Dilophotes* sp.	UPOL TH0054	Java	–	–	KJ404945	KJ405146	–
Dilophotini	*Dilophotes* sp.	UPOL TH0036	Borneo	–	–	KJ404928	KJ405128	–
Dilophotini	*Dilophotes* sp.	UPOL TH0138	Malaysia	–	–	KJ405027	KJ405221	KJ405361
Dilophotini	*Dilophotes* sp.	UPOL TH0127	Mindanao	–	–	KJ405016	KJ405210	KJ405357
Dilophotini	*Dilophotes* sp.	UPOL A00013	Sumatra	–	–	KJ404898	KJ405091	–
Dilophotini	*Dilophotes* sp.	UPOL TH0076	Sumatra	–	–	KJ404965	KJ405164	KJ405314
Dilophotini	*Dilophotes* sp.	UPOL TH0125	Mindanao	–	–	KJ405014	KJ405208	KJ405356
Erotini	*Eropterus nothus*	UPOL 000579	Japan	DQ181082	DQ181156	DQ181008	DQ181230	DQ181384
Erotini	*Eropterus* sp.	UPOL A00548	China	KT752217	KT752379	KT752046	KT751725	KT751883
Erotini	*Eropterus* sp.	UPOL A00549	Laos	–	KT752380	KT752047	KT751726	KT751884
Erotini	*Eropterus* sp.	UPOL A00550	China	KT752218	KT752381	KT752048	KT751727	KT751885
Erotini	*Eropterus* sp.	UPOL A00551	Laos	KT752219	KT752382	KT752049	KT751728	KT751886
Erotini	*Eropterus* sp.	UPOL A00552	Laos	KT752220	KT752383	KT752050	KT751729	KT751887
Erotini	*Eropterus* sp.	UPOL A00553	Slovakia	–	KT752384	KT752051	KT751730	KT751888
Erotini	*Eropterus* sp.	UPOL A00554	Japan	–	KT752385	KT752052	KT751731	KT751889
Erotini	*Eropterus* sp.	UPOL A00558	Japan	KT752224	KT752389	KT752056	KT751735	KT751892
Erotini	*Eropterus* sp.	UPOL 000580	USA	DQ181083	DQ181157	DQ181009	DQ181231	DQ181385
Erotini	*Konoplatycis otome*	UPOL 000575	Japan	DQ181078	DQ181152	DQ181004	DQ181226	DQ181380
Erotini	*Lopheros* sp.	UPOL A00556	Japan	KT752222	KT752387	KT752054	KT751733	KT751891
Erotini	*Lopheros* sp.	UPOL A00567	Laos	KT752233	KT752398	KT752065	KT751743	KT751900
Erotini	*Lopheros* sp.	UPOL 000577	Japan	DQ181080	DQ181154	DQ181006	DQ181228	DQ181382
Erotini	*Lopheros* sp.	UPOL 000578	Japan	DQ181081	DQ181155	DQ181007	DQ181229	DQ181383
Erotini	*Lopheros* sp.	UPOL 001284	Japan	KF625693	KF626279	–	–	–
Erotini	*Platycis comardi*	UPOL A00559	Czech Republic	KT752225	KT752390	KT752057	KT751736	–
Erotini	*Platycis cosnardi*	UPOL 001286	Czech Republic	KF625695	KF626281	KF625984	KF625393	–
Erotini	*Platycis minutus*	UPOL 000348	Czech Republic	DQ181069	DQ181143	DQ181995	DQ181217	DQ181371
Erotini	*Platycis nasutus*	UPOL A00557	Korea	KT752223	KT752388	KT752055	KT751734	–
Erotini	*Platycis nasutus*	UPOL 000576	Japan	DQ181079	DQ181153	DQ181005	DQ181227	DQ181381
Erotini	*Platycis* sp.	UPOL A00547	China	KT752216	KT752378	KT752045	–	KT751882
Erotini	*Platycis* sp.	UPOL A00560	China	KT752226	KT752391	KT752058	–	KT751893
Erotini	*Platycis* sp.	UPOL ZL2008	Japan	–	–	EF143218	EF143233	EF143247
Erotini	*Platycis* sp.	UPOL 001366	USA	KF625697	KF626283	KF625986	KF625395	–
Erotini	*Platycis* sp.	UPOL 001365	China	KF625696	KF626282	KF625985	–	–
Eurrhacini	*Eurrhacini* sp.	UPOL A00622	Nicaragua	KT752285	KT752450	KT752119	KT751793	KT751952
Eurrhacini	*Eurrhacini* sp.	UPOL A00623	Ecuador	KT752286	KT752451	KT752120	KT751794	KT751953
Eurrhacini	*Eurrhacini* sp.	UPOL A00624	Ecuador	–	–	KT752121	KT751795	–
Eurrhacini	*Eurrhacini* sp.	UPOL A00627	Ecuador	–	–	KT752124	KT751797	KT751956
Eurrhacini	*Eurrhacini* sp.	UPOL A00636	Ecuador	KT752294	KT752459	KT752132	KT751806	KT751965
Eurrhacini	*Eurrhacini* sp.	UPOL A00638	Ecuador	–	–	KT752134	–	–
Eurrhacini	*Eurrhacini* sp.	UPOL A00641	Ecuador	–	–	KT752137	KT751810	KT751968
Eurrhacini	*Eurrhacini* sp.	UPOL A00648	Costa Rica	KT752299	KT752464	KT752142	KT751815	–
Eurrhacini	*Eurrhacini* sp.	UPOL A00649	Bolivia	KT752300	KT752465	KT752143	KT751816	KT751972
Eurrhacini	*Eurrhacus* sp.	UPOL 000M43	Ecuador	DQ181056	DQ181130	DQ180982	DQ181204	DQ181358
Leptolycini	Leptolycini sp.	UPOL 000592	Costa Rica	DQ181092	DQ181166	DQ181018	DQ181240	DQ181394
Leptolycini	*Leptolycus* sp.	UPOL VP0022	Puerto Rico	KT752303	KT752469	–	–	KT751976
Leptolycini	*Leptolycus* sp.	UPOL VP0023	Puerto Rico	KT752304	KT752470	–	–	–
Leptolycini	*Leptolycus* sp.	UPOL VP0024	Puerto Rico	KT752305	KT752471	KT752149	–	KT751977
Leptolycini	*Leptolycus* sp.	UPOL VP0025	Puerto Rico	KT752306	KT752472	–	KT751820	–
Leptolycini	*Leptolycus* sp.	UPOL VP0026	Puerto Rico	KT752307	KT752473	KT752150	–	KT751978
Leptolycini	*Leptolycus* sp.	UPOL VP0027	Puerto Rico	KT752308	KT752474	KT752151	KT751821	–
Libnetinae	*Libnetis granicollis*	UPOL 001012	Japan	DQ181107	DQ181181	DQ181033	DQ181255	DQ181409
Libnetinae	*Libnetis* sp.	UPOL A00577	Malaysia	KT752243	–	KT752075	KT751752	KT751909
Libnetinae	*Libnetis* sp.	UPOL A00578	China	KT752244	KT752408	KT752076	KT751753	KT751910
Libnetinae	*Libnetis* sp.	UPOL A00581	China	KT752247	KT752411	KT752079	KT751756	KT751913
Libnetinae	*Libnetis* sp.	UPOL A00582	Laos	KT752248	KT752412	KT752080	KT751757	KT751914
Libnetinae	*Libnetis* sp.	UPOL A00583	China	KT752249	KT752413	KT752081	KT751758	KT751915
Libnetinae	*Libnetis* sp.	UPOL A00584	Laos	KT752250	KT752414	KT752082	KT751759	KT751916
Libnetinae	*Libnetis* sp.	UPOL A00585	Malaysia	KT752251	KT752415	KT752083	KT751760	KT751917
Libnetinae	*Libnetis* sp.	UPOL A00586	Malaysia	KT752252	KT752416	KT752084	KT751761	KT751918
Libnetinae	*Libnetis* sp.	UPOL A00587	Malaysia	KT752253	KT752417	KT752085	KT751762	KT751919
Libnetinae	*Libnetis* sp.	UPOL A00588	Malaysia	KT752254	KT752418	KT752086	KT751763	KT751920
Libnetinae	*Libnetis* sp.	UPOL A00589	Sumatra	KT752255	KT752419	KT752087	KT751764	KT751921
Libnetinae	*Libnetis* sp.	UPOL A00590	Sumatra	KT752256	KT752420	KT752088	KT751765	KT751922
Libnetinae	*Libnetis* sp.	UPOL A00591	Laos	KT752257	KT752421	KT752089	KT751766	KT751923
Libnetinae	*Libnetis* sp.	UPOL 000L02	Borneo	DQ181038	DQ181112	DQ180964	DQ181186	DQ181340
Libnetinae	*Libnetis* sp.	UPOL 001002	Sumatra	DQ181104	DQ181178	DQ181030	DQ181252	DQ181406
Libnetinae	*Libnetis* sp.	UPOL 001008	Malaysia	DQ181105	DQ181179	DQ181031	DQ181253	DQ181407
Lycini	Lycini indet.	UPOL LF0428	Mexico	–	–	–	KF806789	KF806825
Lycini	Lycini indet.	UPOL LF0429	Nicaragua	–	–	KF806861	KF806790	KF806826
Lycini	Lycini indet.	UPOL LF0430	Ecuador	–	–	KF806862	KF806791	KF806827
Lycini	*Lycostomus* sp.	UPOL 000L27	China	DQ181055	DQ181129	DQ180981	DQ181203	DQ181357
Lycini	*Lycostomus* sp.	UPOL A00328	China	–	–	KF806834	KF806756	KF806798
Lycini	*Lycostomus* sp.	UPOL LF0303	Laos	–	–	KF806849	KF806773	–
Lycini	*Lycostomus* sp.	UPOL LF0304	Laos	–	–	KF806850	KF806774	–
Lycini	*Lycostomus* sp.	UPOL LF0308	Laos	–	–	KF806851	KF806775	–
Lycini	*Lycostomus* sp.	UPOL LF0319	India	–	–	–	KF806776	–
Lycini	*Lycostomus* sp.	UPOL LF0320	India	–	–	KF806852	KF806777	–
Lycini	*Lycostomus* sp.	UPOL LF0323	India	–	–	KF806853	KF806778	–
Lycini	*Lycostomus* sp.	UPOL LF0331	Indonesia	–	–	KF806854	KF806779	KF806815
Lycini	*Lycostomus* sp.	UPOL LF0340	Taiwan	–	–	KF806855	KF806780	KF806816
Lycini	*Lycostomus* sp.	UPOL LF0350	Japan	–	–	–	KF806781	KF806817
Lycini	*Lycostomus* sp.	UPOL LF0354	Japan	–	–	KF806856	KF806782	KF806818
Lycini	*Lycostomus* sp.	UPOL LF0359	Taiwan	–	–	–	KF806783	KF806819
Lycini	*Lycostomus* sp.	UPOL LF0365	Malaysia	–	–	KF806857	KF806784	KF806820
Lycini	*Lycostomus* sp.	UPOL LF0370	India	–	–	KF806858	KF806785	KF806821
Lycini	*Lycostomus* sp.	UPOL LF0381	India	–	–	–	KF806786	KF806822
Lycini	*Lycostomus* sp.	UPOL LF0388	Namibia	–	–	KF806859	KF806787	KF806823
Lycini	*Neoycus arizonensis*	UPOL LF0451	USA	–	–	KF806864	KF806793	KF806829
Lycini	*Neoycus arizonensis*	UPOL LF0452	USA	–	–	KF806865	KF806794	KF806830
Lycini	*Neoycus arizonensis*	UPOL LF0454	USA	–	–	KF806866	KF806795	KF806831
Lycini	*Lycostomus loripes*	UPOL LF0464	USA	–	–	KF806867	KF806796	KF806832
Lycini	*Lycostomus loripes*	UPOL LF0466	USA	–	–	KF806868	KF806797	KF806833
Lycini	*Lycus* sp.	UPOL A00469	Zambia	KT752152	KT752310	KT751979	KT751662	KT751824
Lycini	*Lycus* sp.	UPOL 000L03	RSA	DQ181039	DQ181113	DQ180965	DQ181187	DQ181341
Lycini	*Lycus* sp.	UPOL LF0021	Ethiopia	–	–	KF806835	KF806757	KF806799
Lycini	*Lycus* sp.	UPOL LF0031	Cameroon	–	–	KF806836	KF806758	KF806800
Lycini	*Lycus* sp.	UPOL LF0035	Cameroon	–	–	KF806837	KF806759	KF806801
Lycini	*Lycus* sp.	UPOL LF0043	Ethiopia	–	–	KF806838	KF806760	KF806802
Lycini	*Lycus* sp.	UPOL LF0077	RSA	–	–	KF806839	KF806761	KF806803
Lycini	*Lycus* sp.	UPOL LF0093	Tanzania	–	–	–	KF806762	KF806804

Lycini	*Lycus* sp.	UPOL LF0110	Zambia	–	–	KF806840	KF806763	KF806805
Lycini	*Lycus* sp.	UPOL LF0116	RSA	–	–	KF806841	KF806764	KF806806
Lycini	*Lycus* sp.	UPOL LF0143	Zambia	–	–	KF806842	KF806765	KF806807
Lycini	*Lycus* sp.	UPOL LF0167	Ethiopia	–	–	KF806843	KF806766	KF806808
Lycini	*Lycus* sp.	UPOL LF0168	Ethiopia	–	–	KF806844	KF806767	KF806809
Lycini	*Lycus* sp.	UPOL LF0230	Zambia	–	–	–	KF806768	KF806810
Lycini	*Lycus* sp.	UPOL LF0262	Cameroon	–	–	KF806845	KF806769	KF806811
Lycini	*Lycus* sp.	UPOL LF0264	Cameroon	–	–	KF806846	KF806770	KF806812
Lycini	*Lycus* sp.	UPOL LF0266	Cameroon	–	–	KF806847	KF806771	KF806813
Lycini	*Lycus* sp.	UPOL LF0270	Cameroon	–	–	KF806848	KF806772	KF806814
Lycini	*Lycus* sp.	UPOL LF0394	Namibia	–	–	KF806860	KF806788	KF806824
Lycini	*Lycus* sp.	UPOL LF0444	Ethiopia	–	–	KF806863	KF806792	KF806828
Lycoprogenthini	*Lycoprogentes* sp.	UPOL A00523	Java	KT752193	KT752354	KT752024	KT751705	–
Lycoprogenthini	*Lycoprogentes* sp.	UPOL A00524	Malaysia	KT752194	KT752355	–	KT751706	KT751863
Lycoprogenthini	*Lycoprogentes* sp.	UPOL A00530	Malaysia	KT752200	KT752361	KT752029	KT751712	KT751867
Lycoprogenthini	*Lycoprogentes* sp.	UPOL A00532	Java	KT752202	KT752363	KT752031	KT751714	KT751869
Lycoprogenthini	*Lycoprogentes* sp.	UPOL A00533	Sumatra	KT752203	KT752364	KT752032	KT751715	KT751870
Lycoprogenthini	*Lycoprogentes* sp.	UPOL A00541	Sumatra	KT752210	KT752372	KT752039	KT751720	KT751876
Lycoprogenthini	*Lycoprogentes* sp.	UPOL A00542	Sumatra	KT752211	KT752373	KT752040	KT751721	KT751877
Lycoprogenthini	*Lycoprogentes* sp.	UPOL A00545	Sumatra	KT752214	KT752376	KT752043	–	KT751880
Lycoprogenthini	*Lycoprogentes* sp.	UPOL A00572	India	KT752238	KT752403	KT752070	KT751747	KT751904
Lycoprogenthini	*Lycoprogentes* sp.	UPOL A00626	India	KT752288	KT752453	KT752123	–	KT751955
Lycoprogenthini	*Lycoprogentes* sp.	UPOL 000358	Java	DQ181070	DQ181144	DQ180996	DQ181218	DQ181372
Lycoprogenthini	*Lycoprogentes* sp.	UPOL 000801	Sumatra	DQ181095	DQ181169	DQ181021	DQ181243	DQ181397
Lycoprogenthini	*Lycoprogentes* sp.	UPOL 000802	Java	DQ181096	DQ181170	DQ181022	DQ181244	DQ181398
Lycoprogenthini	*Lycoprogentes* sp.	UPOL 000805	Sumatra	DQ181097	DQ181171	DQ181023	DQ181245	DQ181399
Lyponiini	*Lyponia debilis*	UPOL YL0296	China	–	–	ab123456	KJ650468	ab123456
Lyponiini	*Lyponia delicatula*	UPOL 000815	Japan	DQ181099	DQ181173	DQ181025	DQ181247	DQ181401
Lyponiini	*Lyponia kuatunensis*	UPOL YL0319	China	–	–	ab123456	KJ650482	ab123456
Lyponiini	*Lyponia muyuensis*	UPOL YL0314	China	–	–	ab123456	KJ650493	ab123456
Lyponiini	*Lyponia nigrohumeralis*	UPOL 000L17	China	DQ181048	DQ181122	DQ180974	DQ181196	DQ181350
Lyponiini	*Lyponia quadricollis*	UPOL 000817	Korea	DQ181101	DQ181175	DQ181027	DQ181249	DQ181403
Lyponiini	*Lyponia* sp.	UPOL A00571	Laos	KT752237	KT752402	KT752069	KT751746	–
Lyponiini	*Lyponia* sp.	UPOL ZL2014	Japan	–	–	FJ390408	FJ390410	FJ390412
Lyponiini	*Lyponia* sp.	UPOL ZL2016	Japan	–	–	EF143225	EF143240	EF143253
Lyponiini	*Lyponia* sp.	UPOL 000816	China	DQ181100	DQ181174	DQ181026	DQ181248	DQ181402
Lyponiini	*Lyponia* sp.	UPOL A00467	China	KU184276	KU184284	ab123456	KJ650453	ab123456
Lyponiini	*Lyponia tianquanensis*	UPOL A00468	China	KU184277	KU184285	ab123456	KJ650450	ab123456
Lyponiini	*Ponyalis alternata*	UPOL YL0217	China	–	–	ab123456	KJ650408	ab123456
Lyponiini	*Ponyalis fukiensis*	UPOL YL0282	China	–	–	ab123456	KJ650420	ab123456
Lyponiini	*Ponyalis gracilis*	UPOL YL0409	China	–	–	ab123456	KJ650437	ab123456
Lyponiini	*Ponyalis ishigakiana*	UPOL YL0259	Japan	–	–	ab123456	KJ650438	ab123456
Lyponiini	*Ponyalis laticornis*	UPOL A00460	China	KU184270	KU184278	ab123456	KJ650401	ab123456
Lyponiini	*Ponyalis oshimana*	UPOL YL0263	Japan	–	–	ab123456	KJ650415	ab123456
Lyropaeini	*Lyropaeus dominator*	UPOL VP0003	Malaysia	KC736890	KC736899	KC736882	KC736909	KC736918
Lyropaeini	*Lyropaeus optabilis*	UPOL 000585	Malaysia	DQ181088	DQ181162	DQ181014	DQ181236	DQ181390
Lyropaeini	*Lyropaeus optabilis*	UPOL VP0004	Malaysia	KC736891	KC736900	KC736883	KC736910	KC736919
Lyropaeini	*Lyropaeus philippinensis*	UPOL VP0018	Philippines	KC736895	KC736904	–	KC736913	KC736922
Lyropaeini	*Lyropaeus philippinensis*	UPOL VP0019	Philippines	KC736896	KC736905	–	KC736914	KC736923
Lyropaeini	*Lyropaeus ritsemae*	UPOL VP0001	Sumatra	KC736888	KC736898	KC736880	KC736907	KC736916
Lyropaeini	*Lyropaeus ritsemae*	UPOL VP0006	Sumatra	KC736892	KC736901	KC736884	KC736911	KC736920
Lyropaeini	*Lyropaeus rubrostriatus*	UPOL 000L11	Borneo	DQ181042	DQ181116	DQ180968	DQ181190	DQ181344
Lyropaeini	*Lyropaeus* sp.	UPOL A00574	Malaysia	KT752240	KT752405	KT752072	KT751749	KT751906
Lyropaeini	*Lyropaeus* sp.	UPOL VP0016	India	KC736893	KC736902	KC736885	KC736912	KC736921
Lyropaeini	*Lyropaeus* sp.	UPOL VP0017	India	KC736894	KC736903	KC736886	–	–
Lyropaeini	*Lyropaeus* sp.	UPOL VP2312	India	KC736897	KC736906	KC736887	KC736915	KC736924
Lyropaeini	*Lyropaeus waterhouesi*	UPOL VP0002	Sumatra	KC736889	–	KC736881	KC736908	KC736917
Lyropaeini	*Lyropaeus waterhousei*	UPOL 000584	Sumatra	DQ181087	DQ181161	DQ181013	DQ181235	DQ181389
Macrolycini	*Macrolycus atronotatus*	UPOL A00465	China	KU184274	KU184282	ab123456	ab123456	ab123456
Macrolycini	*Macrolycus bicolor*	UPOL YL0403	China	–	–	ab123456	ab123456	ab123456
Macrolycini	*Macrolycus bowringi*	UPOL A00461	China	KU184271	KU184279	ab123456	ab123456	ab123456
Macrolycini	*Macrolycus dotatus*	UPOL YL0185	China	–	–	ab123456	ab123456	ab123456
Macrolycini	*Macrolycus flabellatus*	UPOL YL0032	Japan	–	–	ab123456	ab123456	ab123456
Macrolycini	*Macrolycus galinae*	UPOL A00464	China	KU184273	KU184281	ab123456	ab123456	ab123456
Macrolycini	*Macrolycus ligulatus*	UPOL A00462	China	–	–	ab123456	ab123456	ab123456
Macrolycini	*Macrolycus mucronatus*	UPOL YL0206	China	–	–	ab123456	ab123456	ab123456
Macrolycini	*Macrolycus ochraceus*	UPOL YL0192	China	–	–	ab123456	KJ650495	ab123456
Macrolycini	*Macrolycus oreophilus*	UPOL A00466	China	KU184275	KU184283	ab123456	ab123456	ab123456
Macrolycini	*Macrolycus sichuanensis*	UPOL A00463	China	KU184272	KU184280	ab123456	ab123456	ab123456
Macrolycini	*Macrolycus* sp.	UPOL ZL2005	Japan	–	–	EF143217	EF143232	EF143246
Macrolycini	*Macrolycus* sp.	UPOL 000828	Thailand	DQ181102	DQ181176	DQ181028	DQ181250	DQ181404
Macrolycini	*Macrolycus* sp.	UPOL 000L18	China	DQ181049	DQ181123	DQ180975	DQ181197	DQ181351
Metriorrhynchini	*Broxylus kalamensis*	UPOL MD0107	Sulawesi	KC538221	KC538010	KC538793	KC538414	KC538607
Metriorrhynchini	*Broxylus malinensis*	UPOL MD0101	Sulawesi	KC538219	KC538008	HQ456958	HQ456981	HQ457003
Metriorrhynchini	*Broxylus pendolensis*	UPOL MD0106	Sulawesi	KC538220	KC538009	KC538792	KC538413	KC538606
Metriorrhynchini	*Broxylus pfeifferi*	UPOL MD0099	Sulawesi	KC538218	KC538007	HQ456957	HQ456980	HQ457002
Metriorrhynchini	*Cautires apterus*	UPOL A00652	Tanzania	–	–	KF588381	–	KF588386
Metriorrhynchini	*Cautires apterus*	UPOL A00653	Tanzania	–	–	KF588382	–	KF588387
Metriorrhynchini	*Cautires apterus*	UPOL A00655	Tanzania	–	–	–	KF588384	KF588388
Metriorrhynchini	*Cautires apterus*	UPOL A00656	Tanzania	–	–	KF588383	KF588385	KF588389
Metriorrhynchini	*Cautires cf. montanus*	UPOL 000L06	Borneo	DQ181041	DQ181115	DQ180967	DQ181189	DQ181343
Metriorrhynchini	*Cautires* sp.	UPOL ZL2009	Sumatra	–	–	EF143219	EF143234	EF143248
Metriorrhynchini	*Cautires* sp.	UPOL 000L14	RSA	DQ181045	DQ181119	DQ180971	DQ181193	DQ181347
Metriorrhynchini	*Cautires* sp.	UPOL 000030	Borneo	KC538128	KC537918	KC538632	KC538245	KC538437
Metriorrhynchini	*Cautires* sp.	UPOL 000037	Borneo	KC538129	KC537919	KC538633	KC538246	KC538438
Metriorrhynchini	*Cautires* sp.	UPOL 000040	Borneo	KC538130	KC537920	KC538634	KC538247	KC538439
Metriorrhynchini	*Cautires* sp.	UPOL 000043	Borneo	KC538131	KC537921	KC538635	KC538248	KC538440
Metriorrhynchini	*Cautires* sp.	UPOL 000044	Borneo	KC538132	KC537922	KC538636	KC538249	KC538441
Metriorrhynchini	*Cautires* sp.	UPOL 000047	Sumatra	KC538133	KC537923	KC538637	KC538250	KC538442
Metriorrhynchini	*Cautires* sp.	UPOL 000048	Sumatra	KC538134	KC537924	KC538638	KC538251	KC538443
Metriorrhynchini	*Cautires* sp.	UPOL 000050	Sumatra	KC538135	KC537925	KC538639	KC538252	KC538444
Metriorrhynchini	*Cautires* sp.	UPOL 000052	Sumatra	KC538136	KC537926	KC538640	KC538253	KC538445
Metriorrhynchini	*Cautires* sp.	UPOL 000056	Sumatra	KC538137	KC537927	KC538641	KC538254	KC538446
Metriorrhynchini	*Cautires* sp.	UPOL 000060	Sumatra	KC538138	KC537928	KC538642	KC538255	KC538447
Metriorrhynchini	*Cautires* sp.	UPOL 000064	Laos	KC538139	KC537929	KC538643	KC538256	KC538448
Metriorrhynchini	*Cautires* sp.	UPOL 000066	Laos	KC538140	KC537930	KC538644	KC538257	KC538449
Metriorrhynchini	*Cautires* sp.	UPOL 000068	Borneo	KC538141	KC537931	KC538645	KC538258	KC538450
Metriorrhynchini	*Cautires* sp.	UPOL 000069	Borneo	KC538142	KC537932	KC538646	KC538259	KC538451
Metriorrhynchini	*Cautires* sp.	UPOL 000070	Malaysia	KC538143	KC537933	KC538647	KC538260	KC538452
Metriorrhynchini	*Cautires* sp.	UPOL 000074	Borneo	KC538145	KC537935	KC538649	KC538262	KC538454
Metriorrhynchini	*Cautires* sp.	UPOL 000075	Laos	KC538146	KC537936	–	KC538263	KC538455

Metriorrhynchini	*Cautires* sp.	UPOL 000079	Borneo	KC538147	KC537937	KC538650	KC538264	KC538456
Metriorrhynchini	*Cautires* sp.	UPOL 000080	Borneo	KC538148	KC537938	KC538651	KC538265	KC538457
Metriorrhynchini	*Cautires* sp.	UPOL 000081	Borneo	KC538149	KC537939	KC538652	KC538266	KC538458
Metriorrhynchini	*Cautires* sp.	UPOL 000084	Borneo	KC538150	KC537940	KC538653	KC538267	KC538459
Metriorrhynchini	*Cautires* sp.	UPOL 000088	Malaysia	KC538151	KC537941	KC538654	KC538268	KC538460
Metriorrhynchini	*Cautires* sp.	UPOL 000090	Borneo	KC538152	KC537942	KC538655	KC538269	KC538461
Metriorrhynchini	*Cautires* sp.	UPOL 000104	Borneo	KC538153	KC537943	KC538656	KC538270	KC538462
Metriorrhynchini	*Cautires* sp.	UPOL 000109	Borneo	–	KC537945	KC538658	KC538272	KC538464
Metriorrhynchini	*Cautires* sp.	UPOL 000122	Borneo	KC538157	KC537948	KC538661	KC538275	KC538467
Metriorrhynchini	*Cautires* sp.	UPOL 000123	Java	KC538158	KC537949	–	KC538276	–
Metriorrhynchini	*Cautires* sp.	UPOL 000147	India	KC538161	KC537952	KC538664	KC538278	KC538470
Metriorrhynchini	*Cautires* sp.	UPOL 000164	Laos	KC538165	KC537956	KC538667	KC538282	KC538473
Metriorrhynchini	*Cautires* sp.	UPOL 000174	Malaysia	KC538166	KC537957	–	KC538283	KC538474
Metriorrhynchini	*Cautires* sp.	UPOL 000177	Malaysia	KC538167	–	KC538668	KC538284	KC538475
Metriorrhynchini	*Cautires* sp.	UPOL 000178	Malaysia	KC538168	KC537958	KC538669	KC538285	KC538476
Metriorrhynchini	*Cautires* sp.	UPOL 000188	Laos	KC538170	KC537960	KC538671	KC538287	KC538478
Metriorrhynchini	*Cautires* sp.	UPOL 000189	Laos	KC538171	KC537961	KC538672	KC538288	KC538479
Metriorrhynchini	*Cautires* sp.	UPOL 000195	RSA	KC538172	KC537962	KC538673	KC538289	KC538480
Metriorrhynchini	*Cautires* sp.	UPOL 000205	Sumatra	KC538174	KC537964	KC538675	KC538291	KC538482
Metriorrhynchini	*Cautires* sp.	UPOL 000206	Sumatra	KC538175	KC537965	KC538676	KC538292	KC538483
Metriorrhynchini	*Cautires* sp.	UPOL 000217	Japan	KC538176	KC537966	KC538678	KC538293	KC538484
Metriorrhynchini	*Cautires* sp.	UPOL 000219	Japan	KC538177	KC537967	KC538679	KC538294	KC538485
Metriorrhynchini	*Cautires* sp.	UPOL 000220	Japan	KC538178	KC537968	KC538680	KC538295	KC538486
Metriorrhynchini	*Cautires* sp.	UPOL 000246	Sumatra	KC538181	KC537970	KC538683	KC538298	KC538489
Metriorrhynchini	*Cautires* sp.	UPOL 000290	Laos	KC538184	KC537974	KC538687	KC538302	KC538493
Metriorrhynchini	*Cautires* sp.	UPOL 000294	Sumatra	KC538185	KC537975	KC538688	KC538303	KC538494
Metriorrhynchini	*Cautires* sp.	UPOL 000295	Sumatra	KC538186	KC537976	KC538689	KC538304	KC538495
Metriorrhynchini	*Cautires* sp.	UPOL 000297	Sumatra	KC538187	KC537977	KC538690	KC538305	KC538496
Metriorrhynchini	*Cautires* sp.	UPOL 000314	Sumatra	KC538188	KC537978	KC538691	KC538306	KC538497
Metriorrhynchini	*Cautires* sp.	UPOL 000335	Borneo	–	–	KC538693	KC538308	KC538499
Metriorrhynchini	*Cautires* sp.	UPOL 000339	Borneo	KC538190	KC537980	KC538694	KC538309	KC538500
Metriorrhynchini	*Cautires* sp.	UPOL 000342	Borneo	KC538191	KC537981	KC538695	KC538310	KC538501
Metriorrhynchini	*Cautires* sp.	UPOL 000346	Borneo	KC538192	KC537982	KC538696	KC538311	KC538502
Metriorrhynchini	*Cautires* sp.	UPOL 000355	Java	–	KC537983	KC538697	KC538312	KC538503
Metriorrhynchini	*Cautires* sp.	UPOL 000395	Palawan	–	–	KC538706	KC538319	KC538510
Metriorrhynchini	*Cautires* sp.	UPOL 000403	Palawan	–	KC537990	KC538709	KC538323	KC538514
Metriorrhynchini	*Cautires* sp.	UPOL 000411	Palawan	KC538201	–	KC538710	KC538324	KC538515
Metriorrhynchini	*Cautires* sp.	UPOL 000425	Palawan	–	–	KC538713	KC538327	KC538518
Metriorrhynchini	*Cautires* sp.	UPOL A00017	Taiwan	–	KC537824	–	–	–
Metriorrhynchini	*Cautires* sp.	UPOL A00019	Taiwan	KC538034	KC537826	KC538715	KC538329	KC538520
Metriorrhynchini	*Cautires* sp.	UPOL A00020	Taiwan	KC538035	KC537827	–	–	–
Metriorrhynchini	*Cautires* sp.	UPOL A00021	Taiwan	KC538036	KC537828	HQ456947	HQ456965	–
Metriorrhynchini	*Cautires* sp.	UPOL A00022	Madagascar	KC538037	KC537829	KC538716	HQ456966	HQ456989
Metriorrhynchini	*Cautires* sp.	UPOL A00023	Madagascar	KC538038	KC537830	KC538717	KC538330	KC538521
Metriorrhynchini	*Cautires* sp.	UPOL A00024	Madagascar	KC538039	KC537831	KC538718	KC538331	KC538522
Metriorrhynchini	*Cautires* sp.	UPOL A00025	Madagascar	KC538040	KC537832	KC538719	KC538332	KC538523
Metriorrhynchini	*Cautires* sp.	UPOL A00026	Madagascar	KC538041	KC537833	–	KC538333	KC538524
Metriorrhynchini	*Cautires* sp.	UPOL A00027	Madagascar	KC538042	KC537834	–	KC538334	KC538525
Metriorrhynchini	*Cautires* sp.	UPOL A00028	Madagascar	KC538043	KC537835	–	KC538335	–
Metriorrhynchini	*Cautires* sp.	UPOL A00029	Madagascar	KC538044	KC537836	–	KC538336	KC538526
Metriorrhynchini	*Cautires* sp.	UPOL A00030	Madagascar	KC538045	KC537837	KC538720	KC538337	KC538527
Metriorrhynchini	*Cautires* sp.	UPOL A00048	Malaysia	KC538063	KC537855	HQ456948	HQ456967	HQ456990
Metriorrhynchini	*Cautires* sp.	UPOL A00050	Malaysia	KC538065	KC537857	–	–	KC538546
Metriorrhynchini	*Cautires* sp.	UPOL A00057	Philippines	KC538069	KC537860	–	KC538356	KC538549
Metriorrhynchini	*Cautires* sp.	UPOL A00058	Philippines	KC538070	KC537861	–	KC538357	KC538550
Metriorrhynchini	*Cautires* sp.	UPOL A00062	Philippines	KC538074	KC537865	KC538742	KC538361	KC538554
Metriorrhynchini	*Cautires* sp.	UPOL A00078	Cameroon	KC538089	KC537880	KC538754	KC538375	KC538567
Metriorrhynchini	*Cautires* sp.	UPOL A00079	Cameroon	KC538090	KC537881	KC538755	KC538376	KC538568
Metriorrhynchini	*Cautires* sp.	UPOL A00080	Cameroon	KC538091	KC537882	HQ456950	HQ456969	HQ456992
Metriorrhynchini	*Cautires* sp.	UPOL A00081	Cameroon	KC538093	KC537884	KC538756	KC538377	KC538569
Metriorrhynchini	*Cautires* sp.	UPOL A00082	Cameroon	KC538092	KC537883	KC538757	KC538378	–
Metriorrhynchini	*Cautires* sp.	UPOL A00083	Cameroon	KC538094	KC537885	KC538758	KC538379	KC538570
Metriorrhynchini	*Cautires* sp.	UPOL A00084	Cameroon	KC538095	KC537886	KC538759	KC538380	KC538571
Metriorrhynchini	*Cautires* sp.	UPOL A00085	Cameroon	KC538096	KC537887	KC538760	KC538381	KC538572
Metriorrhynchini	*Cautires* sp.	UPOL A00086	Cameroon	KC538097	KC537888	KC538761	KC538382	KC538573
Metriorrhynchini	*Cautires* sp.	UPOL A00087	Cameroon	KC538098	KC537889	KC538762	KC538383	KC538574
Metriorrhynchini	*Cautires* sp.	UPOL A00088	Cameroon	KC538099	KC537890	KC538763	KC538384	KC538575
Metriorrhynchini	*Cautires* sp.	UPOL A00089	Cameroon	KC538100	KC537891	KC538764	KC538385	KC538576
Metriorrhynchini	*Cautires* sp.	UPOL A00090	Cameroon	KC538101	KC537892	KC538765	KC538386	KC538577
Metriorrhynchini	*Cautires* sp.	UPOL A00092	Cameroon	KC538103	KC537894	KC538766	KC538388	KC538579
Metriorrhynchini	*Cautires* sp.	UPOL A00093	Cameroon	KC538104	KC537895	KC538767	KC538389	KC538580
Metriorrhynchini	*Cautires* sp.	UPOL A00099	Cameroon	KC538110	KC537901	KC538773	KC538393	KC538586
Metriorrhynchini	*Cautires* sp.	UPOL A00100	Cameroon	KC538111	–	KC538774	KC538394	KC538587
Metriorrhynchini	*Cautires* sp.	UPOL A00101	Cameroon	KC538112	KC537902	–	KC538395	–
Metriorrhynchini	*Cautires* sp.	UPOL A00102	Cameroon	KC538113	KC537903	–	KC538396	KC538588
Metriorrhynchini	*Cautires* sp.	UPOL A00103	Cameroon	KC538114	KC537904	–	KC538397	–
Metriorrhynchini	*Cautires* sp.	UPOL A00105	Cameroon	KC538116	KC537906	KC538776	–	KC538590
Metriorrhynchini	*Cautires* sp.	UPOL A00106	Cameroon	KC538117	KC537907	KC538777	KC538398	KC538591
Metriorrhynchini	*Cautires* sp.	UPOL A00107	Cameroon	KC538118	KC537908	–	KC538399	–
Metriorrhynchini	*Cautires* sp.	UPOL A00109	Cameroon	KC538119	KC537909	KC538778	KC538400	KC538592
Metriorrhynchini	*Cautires* sp.	UPOL A00110	Cameroon	KC538120	KC537910	KC538779	–	KC538593
Metriorrhynchini	*Cautires* sp.	UPOL A00111	Cameroon	KC538121	KC537911	KC538780	KC538401	KC538594
Metriorrhynchini	*Cautires* sp.	UPOL A00112	Cameroon	KC538122	KC537912	KC538781	KC538402	KC538595
Metriorrhynchini	*Cautiromimus* sp.	UPOL 000388	Palawan	–	–	–	KC538318	KC538509
Metriorrhynchini	*Ditua* sp.	UPOL A00033	Australia	KC538048	KC537840	KC538723	–	KC538530
Metriorrhynchini	*Leptotrichalus* sp.	UPOL ZL2002	Java	–	–	EF143215	EF143230	EF143244
Metriorrhynchini	*Leptotrichalus* sp.	UPOL 000208	Borneo	DQ181064	DQ181138	DQ180990	DQ181212	DQ181366
Metriorrhynchini	*Leptotrichalus* sp.	UPOL 000396	Palawan	–	–	KC538707	KC538320	KC538511
Metriorrhynchini	*Leptotrichalus* sp.	UPOL 000419	Palawan	KC538203	KC537992	KC538712	KC538326	KC538517
Metriorrhynchini	*Leptotrichalus* sp.	UPOL A00052	Philippines	KC538066	KC537858	HQ456949	HQ456968	HQ456991
Metriorrhynchini	*Leptotrichalus* sp.	UPOL A00059	Philippines	KC538071	KC537862	KC538739	KC538358	KC538551
Metriorrhynchini	*Leptotrichalus* sp.	UPOL A00061	Philippines	KC538073	KC537864	KC538741	KC538360	KC538553
Metriorrhynchini	*Matsudanoeus yuasai*	UPOL VK0248	Japan	–	–	KF652136	–	KF652130
Metriorrhynchini	*Matsudanoeus yuasai*	UPOL VK0249	Japan	–	–	KF652137	KF652120	KF652131
Metriorrhynchini	*Matsudanoeus yuasai*	UPOL VK0395	Japan	–	–	–	–	KF652132
Metriorrhynchini	*Metanoeus* sp.	UPOL 000026	Borneo	KC538127	KC537917	KC538631	KC538244	KC538436
Metriorrhynchini	*Metanoeus* sp.	UPOL 000105	Borneo	KC538154	KC537944	KC538657	KC538271	KC538463
Metriorrhynchini	*Metanoeus* sp.	UPOL 000121	Sumatra	KC538156	KC537947	KC538660	KC538274	KC538466
Metriorrhynchini	*Metanoeus* sp.	UPOL 000125	Sumatra	KC538159	KC537950	KC538662	KC538277	KC538468
Metriorrhynchini	*Metanoeus* sp.	UPOL 000248	Sumatra	KC538182	KC537971	KC538684	KC538299	KC538490
Metriorrhynchini	*Metanoeus* sp.	UPOL 000434	Palawan	–	–	KC538714	KC538328	KC538519

Metriorrhynchini	*Metanoeus* sp.	UPOL A00063	Philippines	KC538075	KC537866	–	KC538362	KC538555
Metriorrhynchini	*Metanoeus* sp.	UPOL A00064	Philippines	KC538076	KC537867	–	KC538363	KC538556
Metriorrhynchini	*Metanoeus* sp.	UPOL A00065	Philippines	KC538077	KC537868	–	KC538364	KC538557
Metriorrhynchini	*Metriorrhynchus palawensis*	UPOL 000366	Palawan	–	–	KC538698	DQ144665	DQ144691
Metriorrhynchini	*Metriorrhynchus lineatus*	UPOL 000L05	Borneo	DQ181040	DQ181114	DQ180966	DQ181188	DQ181342
Metriorrhynchini	*Metriorrhynchus lineatus*	UPOL 000009	Sumatra	KC538123	KC537913	KC538628	DQ904297	DQ904259
Metriorrhynchini	*Metriorrhynchus lobatus*	UPOL 000017	Sulawesi	KC538126	KC537916	KC538630	DQ144662	DQ144688
Metriorrhynchini	*Metriorrhynchus* sp.	UPOL 000010	Sulawesi	KC538124	KC537914	–	DQ144659	DQ144685
Metriorrhynchini	*Metriorrhynchus* sp.	UPOL 000011	Sulawesi	KC538125	KC537915	KC538629	DQ144660	DQ144686
Metriorrhynchini	*Metriorrhynchus* sp.	UPOL 000374	Australia	KC538195	KC537986	KC538701	KC538314	KC538505
Metriorrhynchini	*Metriorrhynchus* sp.	UPOL A00034	Australia	KC538049	KC537841	KC538724	KC538340	KC538531
Metriorrhynchini	*Metriorrhynchus* sp.	UPOL A00038	Australia	KC538053	KC537845	KC538728	KC538344	KC538535
Metriorrhynchini	*Metriorrhynchus* sp.	UPOL A00039	Australia	KC538054	KC537846	KC538729	KC538345	KC538536
Metriorrhynchini	*Metriorrhynchus* sp.	UPOL A00043	Australia	KC538058	KC537850	KC538732	KC538349	KC538540
Metriorrhynchini	*Metriorrhynchus* sp.	UPOL A00046	Australia	KC538061	KC537853	KC538735	KC538352	KC538543
Metriorrhynchini	*Metriorrhynchus* sp.	UPOL A00049	Malaysia	KC538064	KC537856	KC538736	KC538354	KC538545
Metriorrhynchini	*Metriorrhynchus* sp.	UPOL A00116	Papua New Guinea	ab123456	ab123456	ab123456	–	ab123456
Metriorrhynchini	*Metriorrhynchus* sp.	UPOL A00120	Papua New Guinea	ab123456	ab123456	ab123456	–	ab123456
Metriorrhynchini	*Metriorrhynchus* sp.	UPOL A00128	Papua New Guinea	ab123456	ab123456	ab123456	ab123456	ab123456
Metriorrhynchini	*Metriorrhynchus* sp.	UPOL A00132	Papua New Guinea	ab123456	ab123456	ab123456	ab123456	ab123456
Metriorrhynchini	*Metriorrhynchus* sp.	UPOL A00148	Papua New Guinea	ab123456	ab123456	ab123456	ab123456	ab123456
Metriorrhynchini	*Metriorrhynchus* sp.	UPOL A00160	Papua New Guinea	ab123456	ab123456	–	ab123456	ab123456
Metriorrhynchini	*Metriorrhynchus* sp.	UPOL A00164	Papua New Guinea	ab123456	ab123456	ab123456	ab123456	ab123456
Metriorrhynchini	*Metriorrhynchus* sp.	UPOL A00185	Papua New Guinea	ab123456	ab123456	ab123456	ab123456	–
Metriorrhynchini	*Microtrichalus* sp.	UPOL 000L23	Borneo	DQ181052	DQ181126	DQ180978	DQ181200	DQ181354
Metriorrhynchini	*Microtrichalus* sp.	UPOL 000199	Sulawesi	KC538173	KC537963	KC538674	KC538290	KC538481
Metriorrhynchini	*Microtrichalus* sp.	UPOL 000373	Australia	KC538194	KC537985	KC538700	KC538313	KC538504
Metriorrhynchini	*Microtrichalus* sp.	UPOL 000375	Australia	KC538196	KC537987	KC538702	KC538315	KC538506
Metriorrhynchini	*Microtrichalus* sp.	UPOL 000376	Australia	KC538197	KC537988	KC538703	KC538316	KC538507
Metriorrhynchini	*Microtrichalus* sp.	UPOL 000412	Palawan	KC538202	KC537991	KC538711	KC538325	KC538516
Metriorrhynchini	*Microtrichalus* sp.	UPOL A00068	Philippines	KC538080	KC537871	KC538745	KC538367	KC538560
Metriorrhynchini	*Microtrichalus* sp.	UPOL A00069	Philippines	KC538081	KC537872	KC538746	KC538368	KC538561
Metriorrhynchini	*Microtrichalus* sp.	UPOL A00073	Philippines	KC538084	KC537875	KC538749	KC538371	–
Metriorrhynchini	*Microtrichalus* sp.	UPOL MD0097	Sulawesi	KC538216	KC538005	KC538791	HQ456978	HQ457000
Metriorrhynchini	*Microtrichalus* sp.	UPOL MD0098	Sulawesi	KC538217	KC538006	HQ456956	HQ456979	HQ457001
Metriorrhynchini	*Porrostoma haemorrhoidalis*	UPOL 000378	Australia	KC538198	KC537989	KC538704	DQ144679	DQ144703
Metriorrhynchini	*Porrostoma rhipidum*	UPOL 000372	Australia	KC538193	KC537984	KC538699	DQ144678	DQ144702
Metriorrhynchini	*Porrostoma* sp.	UPOL A00035	Australia	KC538050	KC537842	KC538725	KC538341	KC538532
Metriorrhynchini	*Porrostoma* sp.	UPOL A00036	Australia	KC538051	KC537843	KC538726	KC538342	KC538533
Metriorrhynchini	*Porrostoma* sp.	UPOL A00037	Australia	KC538052	KC537844	KC538727	KC538343	KC538534
Metriorrhynchini	*Porrostoma* sp.	UPOL A00040	Australia	KC538055	KC537847	KC538730	KC538346	KC538537
Metriorrhynchini	*Porrostoma* sp.	UPOL A00041	Australia	KC538056	KC537848	KC538731	KC538347	KC538538
Metriorrhynchini	*Porrostoma* sp.	UPOL A00042	Australia	KC538057	KC537849	–	KC538348	KC538539
Metriorrhynchini	*Porrostoma* sp.	UPOL A00044	Australia	KC538059	KC537851	KC538733	KC538350	KC538541
Metriorrhynchini	*Porrostoma* sp.	UPOL A00045	Australia	KC538060	KC537852	KC538734	KC538351	KC538542
Metriorrhynchini	*Sulabanus cordatus*	UPOL MD0069	Sulawesi	KC538213	KC538002	KC538788	KC538410	KC538603
Metriorrhynchini	*Sulabanus cordatus*	UPOL MD0081	Sulawesi	KC538215	KC538004	KC538790	KC538412	KC538605
Metriorrhynchini	*Sulabanus gracilis*	UPOL MD0064	Sulawesi	KC538210	KC537999	KC538786	KC538407	KC538600
Metriorrhynchini	*Sulabanus gracilis*	UPOL MD0067	Sulawesi	KC538212	KC538001	KC538787	KC538409	KC538602
Metriorrhynchini	*Sulabanus gracilis*	UPOL MD0071	Sulawesi	KC538214	KC538003	KC538789	KC538411	KC538604
Metriorrhynchini	*Sulabanus katarinae*	UPOL MD0033	Sulawesi	KC538206	KC537995	KC538783	KC538404	KC538597
Metriorrhynchini	*Sulabanus lalui*	UPOL MD0030	Sulawesi	KC538205	KC537994	–	KC538403	KC538596
Metriorrhynchini	*Sulabanus lineatus*	UPOL MD0034	Sulawesi	KC538207	KC537996	KC538784	KC538405	KC538598
Metriorrhynchini	*Sulabanus mamasensis*	UPOL MD0044	Sulawesi	KC538209	KC537998	KC538785	KC538406	KC538599
Metriorrhynchini	*Sulabanus similis*	UPOL MD0065	Sulawesi	KC538211	KC538000	–	KC538408	KC538601
Metriorrhynchini	*Sulabanus* sp.	UPOL ZL2010	Sulawesi	–	–	EF143220	EF143235	EF143249
Metriorrhynchini	*Sulabanus* sp.	UPOL A00066	Philippines	KC538078	KC537869	KC538743	KC538365	KC538558
Metriorrhynchini	*Sulabanus* sp.	UPOL A00067	Philippines	KC538079	KC537870	KC538744	KC538366	KC538559
Metriorrhynchini	*Sulabanus* sp.	UPOL A00070	Philippines	KC538082	KC537873	KC538747	KC538369	KC538562
Metriorrhynchini	*Sulabanus* sp.	UPOL A00071	Philippines	KC538083	KC537874	KC538748	KC538370	KC538563
Metriorrhynchini	*Sulabanus* sp.	UPOL A00075	Philippines	KC538086	KC537877	KC538751	KC538372	KC538564
Metriorrhynchini	*Sulabanus* sp.	UPOL A00077	Philippines	KC538088	KC537879	KC538753	KC538374	KC538566
Metriorrhynchini	*Synchonnus* sp.	UPOL A00031	Australia	KC538046	KC537838	KC538721	KC538338	KC538528
Metriorrhynchini	*Trichalus* sp.	UPOL A00032	Australia	KC538047	KC537839	KC538722	KC538339	KC538529
Metriorrhynchini	*Wakarumbia aurea*	UPOL MD0136	Sulawesi	KC538235	KC538024	KC538804	KC538427	KC538619
Metriorrhynchini	*Wakarumbia aurea*	UPOL MD0137	Sulawesi	KC538236	KC538025	KC538805	KC538428	KC538620
Metriorrhynchini	*Wakarumbia fasciata*	UPOL MD0121	Sulawesi	KC538226	KC538015	KC538796	KC538418	KC538610
Metriorrhynchini	*Wakarumbia fascicularis*	UPOL MD0140	Sulawesi	KC538237	KC538026	KC538806	KC538429	KC538621
Metriorrhynchini	*Wakarumbia fascicularis*	UPOL MD0145	Sulawesi	KC538239	KC538028	KC538808	KC538431	KC538623
Metriorrhynchini	*Wakarumbia grisea*	UPOL MD0127	Sulawesi	KC538228	KC538017	–	KC538420	KC538612
Metriorrhynchini	*Wakarumbia grisea*	UPOL MD0156	Sulawesi	KC538241	KC538030	KC538810	KC538433	KC538625
Metriorrhynchini	*Wakarumbia kalamensis*	UPOL MD0133	Sulawesi	KC538232	KC538021	KC538801	KC538424	KC538616
Metriorrhynchini	*Wakarumbia kalamensis*	UPOL MD0169	Sulawesi	KC538243	KC538032	KC538812	KC538435	KC538627
Metriorrhynchini	*Wakarumbia kundratai*	UPOL MD0130	Sulawesi	KC538230	KC538019	KC538799	KC538422	KC538614
Metriorrhynchini	*Wakarumbia linearis*	UPOL MD0134	Sulawesi	KC538233	KC538022	KC538802	KC538425	KC538617
Metriorrhynchini	*Wakarumbia mamasensis*	UPOL MD0155	Sulawesi	KC538240	KC538029	KC538809	KC538432	KC538624
Metriorrhynchini	*Wakarumbia mamasensis*	UPOL MD0157	Sulawesi	KC538242	KC538031	KC538811	KC538434	KC538626
Metriorrhynchini	*Wakarumbia monacha*	UPOL MD0111	Sulawesi	KC538223	KC538012	KC538795	KC538416	KC538609
Metriorrhynchini	*Wakarumbia montana*	UPOL MD0119	Sulawesi	KC538225	KC538014	HQ456961	HQ456984	HQ457006
Metriorrhynchini	*Wakarumbia nepeensis*	UPOL MD0129	Sulawesi	KC538229	KC538018	KC538798	KC538421	KC538613
Metriorrhynchini	*Wakarumbia nepeensis*	UPOL MD0135	Sulawesi	KC538234	KC538023	KC538803	KC538426	KC538618
Metriorrhynchini	*Wakarumbia pendolensis*	UPOL MD0109	Sulawesi	KC538222	KC538011	KC538794	KC538415	KC538608
Metriorrhynchini	*Wakarumbia pendolensis*	UPOL MD0143	Sulawesi	KC538238	KC538027	KC538807	KC538430	KC538622
Metriorrhynchini	*Wakarumbia petri*	UPOL MD0118	Sulawesi	KC538224	KC538013	HQ456960	HQ456983	HQ457005
Metriorrhynchini	*Wakarumbia* sp.	UPOL MD0126	Sulawesi	KC538227	KC538016	KC538797	KC538419	KC538611
Metriorrhynchini	*Wakarumbia* sp.	UPOL MD0132	Sulawesi	KC538231	KC538020	KC538800	KC538423	KC538615
Metriorrhynchini	*Xylometanoeus basivittatus*	UPOL 000222	Japan	–	–	–	–	KF652128
Metriorrhynchini	*Xylometanoeus basivittatus*	UPOL 000223	Japan	–	–	–	–	KF652129
Metriorrhynchini	*Xylometanoeus basivittatus*	UPOL VK0075	Japan	–	–	KF652135	KF652115	KF652123
Metriorrhynchini	*Xylometanoeus basivittatus*	UPOL 000221	Japan	KC538179	–	KC538681	KC538296	KC538487
Metriorrhynchini	*Xylobanus kundratai*	UPOL MD0029	Sulawesi	KC538204	KC537993	–	HQ456972	HQ456994
Metriorrhynchini	*Xylobanus kundratai*	UPOL MD0036	Sulawesi	KC538208	KC537997	–	HQ456973	HQ456995
Metriorrhynchini	*Xylobanus* sp.	UPOL 000071	Borneo	KC538144	KC537934	KC538648	KC538261	KC538453
Metriorrhynchini	*Xylobanus* sp.	UPOL 000120	Laos	KC538155	KC537946	KC538659	KC538273	KC538465
Metriorrhynchini	*Xylobanus* sp.	UPOL 000132	Sumatra	KC538160	KC537951	KC538663	HQ456987	HQ457009
Metriorrhynchini	*Xylobanus* sp.	UPOL 000152	Laos	KC538162	KC537953	KC538665	KC538279	KC538471
Metriorrhynchini	*Xylobanus* sp.	UPOL 000153	Laos	KC538163	KC537954	KC538666	KC538280	KC538472
Metriorrhynchini	*Xylobanus* sp.	UPOL 000154	Laos	KC538164	KC537955	–	KC538281	–
Metriorrhynchini	*Xylobanus* sp.	UPOL 000184	Borneo	KC538169	KC537959	KC538670	KC538286	KC538477
Metriorrhynchini	*Xylobanus* sp.	UPOL 000224	Japan	KC538180	KC537969	KC538682	KC538297	KC538488
Metriorrhynchini	*Xylobanus* sp.	UPOL 000262	Borneo	–	KC537972	KC538685	KC538300	KC538491

Metriorrhynchini	*Xylobanus* sp.	UPOL 000274	Borneo	KC538183	KC537973	KC538686	KC538301	KC538492
Metriorrhynchini	*Xylobanus* sp.	UPOL 000315	Sumatra	KC538189	KC537979	KC538692	KC538307	KC538498
Metriorrhynchini	*Xylobanus* sp.	UPOL 000379	Palawan	KC538199	–	KC538705	KC538317	KC538508
Metriorrhynchini	*Xylobanus* sp.	UPOL 000402	Palawan	KC538200	–	KC538708	KC538322	KC538513
Metriorrhynchini	*Xylobanus* sp.	UPOL A00018	Taiwan	KC538033	KC537825	HQ456946	HQ456964	HQ456988
Metriorrhynchini	*Xylobanus* sp.	UPOL A00053	Palawan	KC538067	KC537859	KC538737	KC538355	KC538547
Metriorrhynchini	*Xylobanus* sp.	UPOL A00054	Palawan	KC538068	–	KC538738	–	KC538548
Metriorrhynchini	*Xylobanus* sp.	UPOL A00074	Philippines	KC538085	KC537876	KC538750	–	–
Metriorrhynchini	*Xylobanus* sp.	UPOL A00076	Philippines	KC538087	KC537878	KC538752	KC538373	KC538565
Metriorrhynchini	*Xylobanus* sp.	UPOL A00091	Cameroon	KC538102	KC537893	–	KC538387	KC538578
Metriorrhynchini	*Xylobanus* sp.	UPOL A00094	Cameroon	KC538105	KC537896	KC538768	KC538390	KC538581
Metriorrhynchini	*Xylobanus* sp.	UPOL A00095	Cameroon	KC538106	KC537897	KC538769	–	KC538582
Metriorrhynchini	*Xylobanus* sp.	UPOL A00096	Cameroon	KC538107	KC537898	KC538770	–	KC538583
Metriorrhynchini	*Xylobanus* sp.	UPOL A00097	Cameroon	KC538108	KC537899	KC538771	KC538391	KC538584
Metriorrhynchini	*Xylobanus* sp.	UPOL A00098	Cameroon	KC538109	KC537900	KC538772	KC538392	KC538585
Metriorrhynchini	*Xylobanus* sp.	UPOL A00104	Cameroon	KC538115	KC537905	KC538775	–	KC538589
Metriorrhynchini	*Xylometanoeus japonicus*	UPOL VK0038	Japan	–	–	–	KF652116	KF652124
Metriorrhynchini	*Xylometanoeus japonicus*	UPOL VK0039	Japan	–	–	–	KF652117	KF652125
Metriorrhynchini	*Xylometanoeus japonicus*	UPOL VK0090	Japan	–	–	–	KF652118	KF652126
Metriorrhynchini	*Xylometanoeus japonicus*	UPOL VK0093	Japan	–	–	–	KF652119	KF652127
Platerodini	*Plateros* sp.	UPOL A00470	Malaysia	KT752153	KT752311	KT751980	KT751663	KT751825
Platerodini	*Plateros* sp.	UPOL A00471	Malaysia	KT752154	KT752312	KT751981	KT751664	KT751826
Platerodini	*Plateros* sp.	UPOL A00472	Malaysia	KT752155	KT752313	KT751982	KT751665	–
Platerodini	*Plateros* sp.	UPOL A00473	Malaysia	–	KT752314	KT751983	KT751666	KT751827
Platerodini	*Plateros* sp.	UPOL A00474	Malaysia	KT752156	KT752315	KT751984	KT751667	–
Platerodini	*Plateros* sp.	UPOL A00475	Malaysia	KT752157	KT752316	KT751985	–	KT751828
Platerodini	*Plateros* sp.	UPOL A00476	Malaysia	KT752158	KT752317	KT751986	KT751668	–
Platerodini	*Plateros* sp.	UPOL A00478	Vietnam	KT752160	KT752319	KT751988	KT751670	KT751830
Platerodini	*Plateros* sp.	UPOL A00479	Vietnam	KT752161	KT752320	KT751989	KT751671	KT751831
Platerodini	*Plateros* sp.	UPOL A00480	Laos	KT752162	KT752321	KT751990	KT751672	KT751832
Platerodini	*Plateros* sp.	UPOL A00481	Laos	KT752163	KT752322	KT751991	–	KT751833
Platerodini	*Plateros* sp.	UPOL A00482	Laos	–	KT752323	KT751992	KT751673	KT751834
Platerodini	*Plateros* sp.	UPOL A00483	Laos	KT752164	–	KT751993	KT751674	KT751835
Platerodini	*Plateros* sp.	UPOL A00484	Thailand	KT752165	KT752324	KT751994	KT751675	KT751836
Platerodini	*Plateros* sp.	UPOL A00485	India	KT752166	KT752325	KT751995	KT751676	KT751837
Platerodini	*Plateros* sp.	UPOL A00486	India	–	KT752326	KT751996	KT751677	KT751838
Platerodini	*Plateros* sp.	UPOL A00487	India	KT752167	KT752327	KT751997	KT751678	KT751839
Platerodini	*Plateros* sp.	UPOL A00488	Taiwan	–	KT752328	KT751998	KT751679	KT751840
Platerodini	*Plateros* sp.	UPOL A00489	Taiwan	KT752168	KT752329	KT751999	KT751680	KT751841
Platerodini	*Plateros* sp.	UPOL A00490	Taiwan	KT752169	KT752330	KT752000	KT751681	KT751842
Platerodini	*Plateros* sp.	UPOL A00491	Japan	KT752170	KT752331	KT752001	KT751682	KT751843
Platerodini	*Plateros* sp.	UPOL A00492	Japan	KT752171	KT752332	KT752002	KT751683	KT751844
Platerodini	*Plateros* sp.	UPOL A00493	Kenya	KT752172	KT752333	KT752003	KT751684	KT751845
Platerodini	*Plateros* sp.	UPOL A00494	Cameroon	KT752173	KT752334	KT752004	KT751685	KT751846
Platerodini	*Plateros* sp.	UPOL A00495	Cameroon	KT752174	KT752335	KT752005	KT751686	KT751847
Platerodini	*Plateros* sp.	UPOL A00496	Cameroon	KT752175	KT752336	KT752006	KT751687	–
Platerodini	*Plateros* sp.	UPOL A00497	Cameroon	KT752176	KT752337	KT752007	KT751688	–
Platerodini	*Plateros* sp.	UPOL A00498	Cameroon	KT752177	KT752338	KT752008	KT751689	KT751848
Platerodini	*Plateros* sp.	UPOL A00499	Zambia	KT752178	KT752339	KT752009	KT751690	KT751849
Platerodini	*Plateros* sp.	UPOL A00500	Zambia	KT752179	KT752340	KT752010	KT751691	KT751850
Platerodini	*Plateros* sp.	UPOL A00501	RSA	KT752180	KT752341	KT752011	KT751692	KT751851
Platerodini	*Plateros* sp.	UPOL A00502	Cameroon	KT752181	KT752342	KT752012	KT751693	KT751852
Platerodini	*Plateros* sp.	UPOL A00503	Cameroon	KT752182	KT752343	KT752013	KT751694	KT751853
Platerodini	*Plateros* sp.	UPOL A00504	Cameroon	KT752183	KT752344	KT752014	KT751695	KT751854
Platerodini	*Plateros* sp.	UPOL A00505	Canada	KT752184	KT752345	KT752015	KT751696	KT751855
Platerodini	*Plateros* sp.	UPOL A00507	Canada	KT752185	KT752346	KT752016	KT751697	KT751856
Platerodini	*Plateros* sp.	UPOL A00509	Argentina	KT752186	KT752347	KT752017	KT751698	KT751857
Platerodini	*Plateros* sp.	UPOL A00510	Argentina	KT752187	KT752348	KT752018	KT751699	KT751858
Platerodini	*Plateros* sp.	UPOL A00511	Costa Rica	KT752188	KT752349	KT752019	KT751700	KT751859
Platerodini	*Plateros* sp.	UPOL A00512	Costa Rica	KT752189	KT752350	KT752020	KT751701	KT751860
Platerodini	*Plateros* sp.	UPOL A00513	Panama	KT752190	KT752351	KT752021	KT751702	KT751861
Platerodini	*Plateros* sp.	UPOL A00555	Thailand	KT752221	KT752386	KT752053	KT751732	KT751890
Platerodini	*Plateros* sp.	UPOL A00579	Nicaragua	KT752245	KT752409	KT752077	KT751754	KT751911
Platerodini	*Plateros* sp.	UPOL A00580	USA	KT752246	KT752410	KT752078	KT751755	KT751912
Platerodini	*Plateros* sp.	UPOL A00619	Ecuador	–	KT752448	KT752117	KT751791	KT751950
Platerodini	*Plateros* sp.	UPOL A00632	Ecuador	KT752290	KT752455	–	KT751802	KT751961
Platerodini	*Plateros* sp.	UPOL A00634	Ecuador	KT752292	KT752457	KT752130	KT751804	KT751963
Platerodini	*Plateros* sp.	UPOL A00635	Nicaragua	KT752293	KT752458	KT752131	KT751805	KT751964
Platerodini	*Plateros* sp.	UPOL A00639	Ecuador	–	–	KT752135	KT751808	KT751966
Platerodini	*Plateros* sp.	UPOL A00643	Ecuador	–	KT752461	KT752138	KT751811	KT751969
Platerodini	*Plateros* sp.	UPOL A00646	Ecuador	KT752297	–	KT752140	KT751813	KT751971
Platerodini	*Plateros* sp.	UPOL ZL2012	Japan	–	–	EF143222	EF143237	EF143251
Platerodini	*Plateros* sp.	UPOL ZL2018	Japan	–	–	EF143227	EF143241	EF143255
Platerodini	*Plateros* sp.	UPOL 000031	Borneo	DQ181059	DQ181133	DQ180985	DQ181207	DQ181361
Platerodini	*Plateros* sp.	UPOL 000243	Borneo	DQ181065	DQ181139	DQ180991	DQ181213	DQ181367
Platerodini	*Plateros* sp.	UPOL 000303	Sumatra	DQ181067	DQ181141	DQ180993	DQ181215	DQ181369
Platerodini	*Plateros* sp.	UPOL 000L13	Borneo	DQ181044	DQ181118	DQ180970	DQ181192	DQ181346
Platerodini	*Plateros* sp.	UPOL 001031	USA	DQ181109	DQ181183	DQ181035	DQ181257	DQ181411
Platerodini	*Plateros* sp.	UPOL RK0377	French Guyana	KF625685	KF626271	KF625975	KF625384	–
Platerodrilini	*Horakiella emasensis*	UPOL 001043	Borneo	DQ181110	DQ181184	DQ181036	DQ181258	DQ181412
Platerodrilini	*Macrolibnetis depressus*	UPOL 000515	Malaysia	–	–	–	FJ390411	FJ390413
Platerodrilini	*Macrolibnetis depressus*	UPOL 000L21	Malaysia	DQ181050	DQ181124	DQ180976	DQ181198	DQ181352
Platerodrilini	*Macrolibnetis depressus*	UPOL VP0050	Malaysia	KF802504	KF802506	KF802467	–	KF802538
Platerodrilini	*Pendola* sp.	UPOL 000M45	Java	DQ181058	DQ181132	DQ180984	DQ181206	DQ181360
Platerodrilini	*Platerodrilini* gen. sp.	UPOL VP0009	Malaysia	KF802497	KF802507	KF802457	KF802527	KF802539
Platerodrilini	*Platerodrilini* gen. sp.	UPOL VP0010	Sumatra	KF802498	KF802508	KF802480	KF802533	KF802542
Platerodrilini	*Platerodrilini* gen. sp.	UPOL VP0012	Malaysia	KF802499	KF802509	KF802458	KF802528	KF802541
Platerodrilini	*Platerodrilini* gen. sp.	UPOL VP0013	Malaysia	KF802500	KF802510	–	KF802529	KF802540
Platerodrilini	*Platerodrilini* gen. sp.	UPOL VP0030	India	KF802493	KF802511	KF802462	KF802530	KF802543
Platerodrilini	*Platerodrilini* gen. sp.	UPOL VP0031	India	KF802495	KF802512	KF802463	KF802531	–
Platerodrilini	*Platerodrilini* gen. sp.	UPOL VP0034	India	KF802496	KF802513	KF802464	KF802532	KF802544
Platerodrilini	*Platerodrilus angustatus*	UPOL 001388	Sumatra	KF625713	KF626299	KF626001	KF625406	–
Platerodrilini	*Platerodrilus atricolor*	UPOL 001384	Malaysia	KF625710	KF626296	–	–	–
Platerodrilini	*Platerodrilus corporaali*	UPOL 001373	Sumatra	KF625702	KF626288	KF625991	KF625400	–
Platerodrilini	*Platerodrilus curtus*	UPOL 001380	Philippines	KF625708	KF626294	KF625997	KF625404	–
Platerodrilini	*Platerodrilus curtus*	UPOL 001381	Philippines	KF625781	KF626365	KF626073	–	–
Platerodrilini	*Platerodrilus curtus*	UPOL 001383	Philippines	KF625782	KF626366	KF626074	KF625474	–
Platerodrilini	*Platerodrilus foliaceus*	UPOL 000588	Borneo	DQ181091	DQ181165	DQ181017	DQ181239	DQ181393
Platerodrilini	*Platerodrilus foliaceus*	UPOL 000589	Borneo	–	–	EF143214	EF143229	EF143243
Platerodrilini	*Platerodrilus ijenensis*	UPOL 000586	Java	DQ181089	DQ181163	DQ181015	DQ181237	DQ181391
Platerodrilini	*Platerodrilus luteus*	UPOL 001379	Sumatra	KF625707	KF626293	KF625996	KF625403	–

Platerodrilini	*Platerodrilus major*	UPOL 001387	Sumatra	KF625712	KF626298	KF626000	–	–
Platerodrilini	*Platerodrilus maninjauensis*	UPOL 001374	Sumatra	KF625703	KF626289	KF625992	KF625401	–
Platerodrilini	*Platerodrilus maninjauensis*	UPOL 001377	Sumatra	KF625705	KF626291	KF625994	–	–
Platerodrilini	*Platerodrilus maninjauensis*	UPOL VP2303	Sumatra	KF802485	KF802514	KF802470	–	KF802545
Platerodrilini	*Platerodrilus maninjauensis*	UPOL VP2306	Sumatra	KF802487	KF802515	KF802473	–	KF802546
Platerodrilini	*Platerodrilus maninjauensis*	UPOL VP2307	Sumatra	KF802488	KF802516	KF802474	–	KF802547
Platerodrilini	*Platerodrilus montanus*	UPOL 001371	Sumatra	KF625700	KF626286	KF625989	KF625398	–
Platerodrilini	*Platerodrilus montanus*	UPOL VP2308	Sumatra	KF802489	KF802517	KF802475	–	KF802549
Platerodrilini	*Platerodrilus ngi*	UPOL VP0021	Singapore	KF802481	KF802520	KF802461	–	–
Platerodrilini	*Platerodrilus palawanensis*	UPOL 000371	Philippines	–	–	–	–	KF802552
Platerodrilini	*Platerodrilus ranauensis*	UPOL 000587	Sumatra	DQ181090	DQ181164	DQ181016	DQ181238	DQ181392
Platerodrilini	*Platerodrilus robinsoni*	UPOL 001378	Sumatra	KF625706	KF626292	KF625995	–	–
Platerodrilini	*Platerodrilus sibayakensis*	UPOL 001372	Sumatra	KF625701	KF626287	KF625990	KF625399	–
Platerodrilini	*Platerodrilus sibayakensis*	UPOL 001389	Sumatra	KF625784	KF626367	–	KF625475	–
Platerodrilini	Platerodrilus sp.	UPOL 000L01	Borneo	DQ181037	DQ181111	DQ180963	DQ181185	DQ181339
Platerodrilini	*Platerodrilus* sp.	UPOL VP0014	Philippines	KF802482	KF802519	KF802459	–	KF802553
Platerodrilini	*Platerodrilus* sp.	UPOL VP0020	Malaysia	KF802501	–	KF802460	KF802535	KF802554
Platerodrilini	*Platerodrilus* sp.	UPOL VP0044	Borneo	KF802505	–	KF802465	–	KF802550
Platerodrilini	*Platerodrilus* sp.	UPOL VP0047	Sumatra	KF802502	–	KF802466	–	KF802555
Platerodrilini	*Platerodrilus* sp.	UPOL VP2301	Borneo	KF802483	KF802518	KF802468	–	KF802551
Platerodrilini	*Platerodrilus* sp.	UPOL VP2302	Malaysia	KF802484	KF802521	KF802469	–	KF802556
Platerodrilini	*Platerodrilus* sp.	UPOL VP2304	Thailand	KF802486	KF802522	KF802471	–	–
Platerodrilini	*Platerodrilus* sp.	UPOL VP2309	Malaysia	KF802490	KF802524	KF802476	–	KF802557
Platerodrilini	*Platerodrilus* sp.	UPOL VP2310	Malaysia	KF802491	KF802525	KF802477	KF802536	KF802558
Platerodrilini	*Platerodrilus* sp.	UPOL VP2311	Laos	KF802503	–	KF802478	–	KF802559
Platerodrilini	*Platerodrilus* sp.	UPOL VP2316	Philippines	KF802492	KF802526	KF802479	–	KF802560
Platerodrilini	*Platerodrilus* sp.	UPOL A00575	Malaysia	KT752241	KT752406	KT752073	KT751750	KT751907
Platerodrilini	*Platerodrilus* sp.	UPOL A00576	Malaysia	KT752242	KT752407	KT752074	KT751751	KT751908
Platerodrilini	*Platerodrilus strbai*	UPOL 000472	Borneo	–	–	–	KF802537	KF802561
Platerodrilini	*Platerodrilus talamauensis*	UPOL 001375	Sumatra	KF625780	KF626364	KF626072	KF625473	–
Platerodrilini	*Platerodrilus talamauensis*	UPOL 001376	Sumatra	KF625704	KF626290	KF625993	KF625402	–
Platerodrilini	*Platerodrilus tujuhensis*	UPOL 001385	Sumatra	KF625711	KF626297	KF625999	KF625405	–
Platerodrilini	*Platerodrilus tujuhensis*	UPOL VP2305	Sumatra	KF802494	KF802523	KF802472	–	KF802548
Slipinskiini	*Flagrax* sp.	UPOL A00561	RSA	KT752227	KT752392	KT752059	KT751737	KT751894
Slipinskiini	*Flagrax* sp.	UPOL A00562	Cameroon	KT752228	KT752393	KT752060	KT751738	KT751895
Slipinskiini	*Flagrax* sp.	UPOL A00563	Cameroon	KT752229	KT752394	KT752061	KT751739	KT751896
Slipinskiini	*Flagrax* sp.	UPOL A00564	RSA	KT752230	KT752395	KT752062	KT751740	KT751897
Slipinskiini	*Flagrax* sp.	UPOL A00565	RSA	KT752231	KT752396	KT752063	KT751741	KT751898
Slipinskiini	*Flagrax* sp.	UPOL A00566	RSA	KT752232	KT752397	KT752064	KT751742	KT751899
Slipinskiini	*Flagrax* sp.	UPOL 000L26	RSA	DQ181054	DQ181128	DQ180980	DQ181202	DQ181356
Taphini	*Protaphes* sp.	UPOL A00525	Laos	KT752195	KT752356	KT752025	KT751707	–
Taphini	*Protaphes* sp.	UPOL A00527	Sumatra	KT752197	KT752358	KT752026	KT751709	–
Taphini	*Protaphes* sp.	UPOL A00531	Malaysia	KT752201	KT752362	KT752030	KT751713	KT751868
Taphini	*Protaphes* sp.	UPOL A00535	Sumatra	KT752204	KT752366	KT752034	KT751717	KT751872
Taphini	*Protaphes* sp.	UPOL A00543	Malaysia	KT752212	KT752374	KT752041	KT751722	KT751878
Taphini	*Protaphes* sp.	UPOL A00544	Malaysia	KT752213	KT752375	KT752042	KT751723	KT751879
Taphini	*Protaphes* sp.	UPOL A00573	Laos	KT752239	KT752404	KT752071	KT751748	KT751905
Taphini	*Taphes brevicollis*	UPOL A00526	Laos	KT752196	KT752357	–	KT751708	KT751864
Taphini	*Taphes brevicollis*	UPOL A00528	Laos	KT752198	KT752359	KT752027	KT751710	KT751865
Taphini	*Taphes brevicollis*	UPOL A00536	Sumatra	KT752205	KT752367	KT752035	KT751718	KT751873
Taphini	*Taphes brevicollis*	UPOL A00538	Sumatra	KT752207	KT752369	–	KT751719	KT751875
Taphini	*Taphes brevicollis*	UPOL 000812	Laos	DQ181098	DQ181172	DQ181024	DQ181246	DQ181400
Taphini	*Taphes* sp.	UPOL A00529	India	KT752199	KT752360	KT752028	KT751711	KT751866
Taphini	*Taphes* sp.	UPOL A00537	China	KT752206	KT752368	KT752036	–	KT751874
Taphini	*Taphes* sp.	UPOL A00539	Laos	KT752208	KT752370	KT752037	–	–
Thonalmini	*Thonalmus hubbardi*	UPOL 000595	Montserrat	DQ181094	DQ181168	DQ181020	DQ181242	DQ181396
Thonalmini	*Thonalmus sinuaticostis*	UPOL 000594	Montserrat	DQ181093	DQ181167	DQ181019	DQ181241	DQ181395
Thonalmini	*Thonalmus* sp.	UPOL A00592	Cuba	KT752258	KT752422	KT752090	KT751767	KT751924
Thonalmini	*Thonalmus* sp.	UPOL A00593	Cuba	KT752259	KT752423	KT752091	KT751768	KT751925
Thonalmini	*Thonalmus* sp.	UPOL A00595	Dominican Republic	KT752261	KT752425	KT752093	KT751769	KT751927

Supplementary Table S2. Tribe-level taxa not included in the molecular analysis.

Tribe	Number of spp.	Distribution
Melanerotini Kazantsev, 2010	1	Pacific islands
Proterotaphini Kazantsev, 2012	1	Australia
Vikhreviini Kazantsev, 2013	1	Malay Peninsula
Mimolibnetinae Kazantsev, 2013	3	Afrotropical region
Dominopterini Kazantsev, 2013	1	Antilles
Electropterini Kazantsev, 2013	3	Antilles
Miniduliticolini Kazantsev, 2002	1	Borneo

Kazantsev S.V. (2002) A generic review of Duliticolinae, new subfamily (Coleoptera: Lycidae). *Elytron* 16: 5–21.

Kazantsev S.V. (2009) New taxa of Lycidae from Samoa, Fiji and Tonga(Coleoptera: Lycidae). *Russian Entomological Journal* 18: 191–195.

Kazantsev S.V. (2012) A review of Erotinae and Dictyopterinae (Coleoptera: Lycidae), with description of new taxa and a note on biogeography of the subfamilies. *Russian Entomological Journal* 21: 395–414.

Kazantsev S.V. (2013) New and little known taxa of neotenic Lycidae (Coleoptera), with discussion of their phylogeny. *Russian Entomological Journal* 22: 9–31.

Table S2. Primers used for PCR amplification.

Fragment	Code	Sequence (5’ > 3’)
18S rRNA	5’	GACAACCTGGTTGATCCTGCCAGT
	b5.0	TAACCGCAACAACTTTAAT
	ai	CCTGAGAAACGGCTACCACATC
	b2.5	TCTTTGGCAAA TGCTTTCGC
	a1.0	GGTGAAATTCTTGGACCGTC
	bi	GAGTCTCGTTCGTTATCGGA
	a2.0	ATGGTTGCAAAGCTGAAAC
	3’I	CACCTACGGAAACCTTGTTACGAC
28S rRNA	ff	TTA CAC ACT CCT TAG CGG AT
	dd	GGG ACC CGT CTT GAA ACA C
rrnL mtDNA	16a	CGC CTG TTT AAC AAA AAC AT
	ND1A	GGT CCC TTA CGA ATT TGA ATA TAT CCT
	ND1-2	ATC AAA AGG AGC TCG ATT AGT TTC
cox1 mtDNA	JerryM	CAA CAY YTA TTT TGR TTY TTT GG
	MarcyM	TAR TTC RTA TGW RCA ATA YCA YTG RTG
	JerryN	CAA CAY YTA TTY TGA TTY TTY GG
	MarcyN	TTC RTA WGT TCA RTA TCA TTG RTG
nad5 mtDNA	OF1	CCT ACT CCT GTT TCT GCT TTA GTT CAT TC
	R6	GAA ACG AAA AAT CGT ATT TAA TTT CGA CT
	R2M	AAT TGA ASC CAA AAA GAG GTA TAT CAC TG

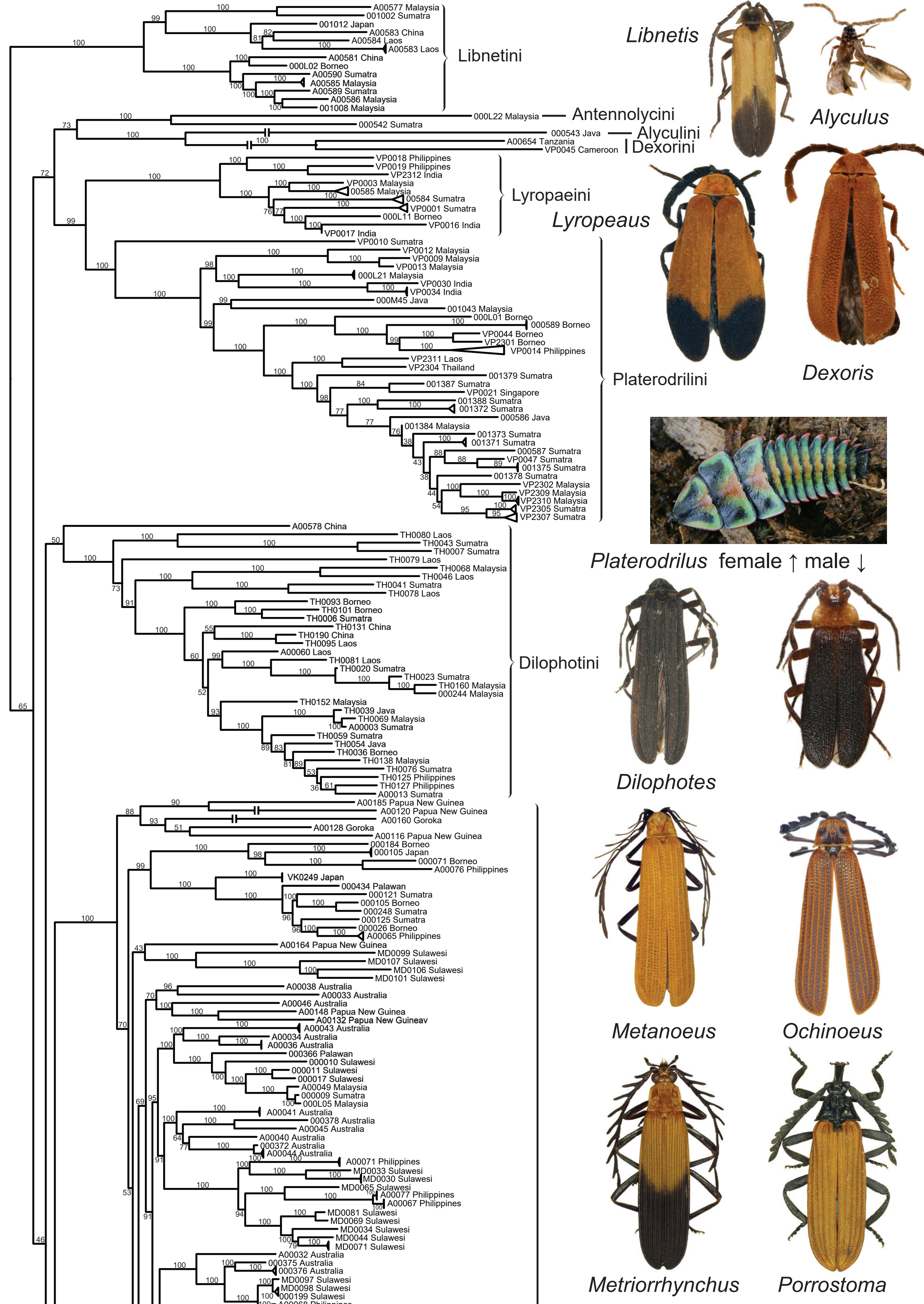

A00577 Malaysia
001002 Sumatra
001012 Japan
A00583 China
A00584 Laos
A00583 Laos
A00581 China
000L02 Borneo
A00590 Sumatra
A00585 Malaysia
A00589 Sumatra
A00586 Malaysia
001008 Malaysia
Libnetini
000L22 Malaysia
Antennolycini
000542 Sumatra
000543 Java
Alyculini
A00654 Tanzania
VP0045 Cameroon
Dexorini
VP0018 Philippines
VP0019 Philippines
VP2312 India
VP0003 Malaysia
00585 Malaysia
00584 Sumatra
VP0001 Sumatra
000L11 Borneo
VP0016 India
VP0017 India
Lyropaeini
VP0010 Sumatra
VP0012 Malaysia
VP0009 Malaysia
VP0013 Malaysia
000L21 Malaysia
VP0030 India
VP0034 India
000M45 Java
001043 Malaysia
000L01 Borneo
000589 Borneo
VP0044 Borneo
VP2301 Borneo
VP0014 Philippines
VP2311 Laos
VP2304 Thailand
001379 Sumatra
001387 Sumatra
VP0021 Singapore
001388 Sumatra
001372 Sumatra
000586 Java
001384 Malaysia
001373 Sumatra
001371 Sumatra
000587 Sumatra
VP0047 Sumatra
001375 Sumatra
001378 Sumatra
VP2302 Malaysia
VP2309 Malaysia
VP2310 Malaysia
VP2305 Sumatra
VP2307 Sumatra
Platerodrilini
A00578 China
TH0080 Laos
TH0043 Sumatra
TH0007 Sumatra
TH0079 Laos
TH0068 Malaysia
TH0046 Laos
TH0041 Sumatra
TH0078 Laos
TH0093 Borneo
TH0101 Borneo
TH0006 Sumatra
TH0131 China
TH0190 China
TH0095 Laos
A00060 Laos
TH0081 Laos
TH0020 Sumatra
TH0023 Sumatra
TH0160 Malaysia
000244 Malaysia
TH0152 Malaysia
TH0039 Java
TH0069 Malaysia
A00003 Sumatra
TH0059 Sumatra
TH0054 Java
TH0036 Borneo
TH0138 Malaysia
TH0076 Sumatra
TH0125 Philippines
TH0127 Philippines
A00013 Sumatra
Dilophotini
A00185 Papua New Guinea
A00120 Papua New Guinea
A00160 Goroka
A00128 Goroka
A00116 Papua New Guinea
000184 Borneo
000105 Japan
000071 Borneo
A00076 Philippines
VK0249 Japan
000434 Palawan
000121 Sumatra
000105 Borneo
000248 Sumatra
000125 Sumatra
000026 Borneo
A00065 Philippines
A00164 Papua New Guinea
MD0099 Sulawesi
MD0107 Sulawesi
MD0106 Sulawesi
MD0101 Sulawesi
A00038 Australia
A00033 Australia
A00046 Australia
A00148 Papua New Guinea
A00132 Papua New Guineav
A00043 Australia
A00034 Australia
A00036 Australia
000366 Palawan
000010 Sulawesi
000011 Sulawesi
000017 Sulawesi
A00049 Malaysia
000009 Sumatra
000L05 Malaysia
A00041 Australia
000378 Australia
A00045 Australia
A00040 Australia
000372 Australia
A00044 Australia
A00071 Philippines
MD0033 Sulawesi
MD0030 Sulawesi
MD0065 Sulawesi
A00077 Philippines
A00067 Philippines
MD0081 Sulawesi
MD0069 Sulawesi
MD0034 Sulawesi
MD0044 Sulawesi
MD0071 Sulawesi
A00032 Australia
000375 Australia
000376 Australia
MD0097 Sulawesi
MD0098 Sulawesi
000199 Sulawesi
Libnetis
Alyculus
Lyropeaus
Dexoris
Platerodrilus female ↑ male ↓
Dilophotes
Metanoeus
Ochinoeus
Metriorrhynchus
Porrostoma

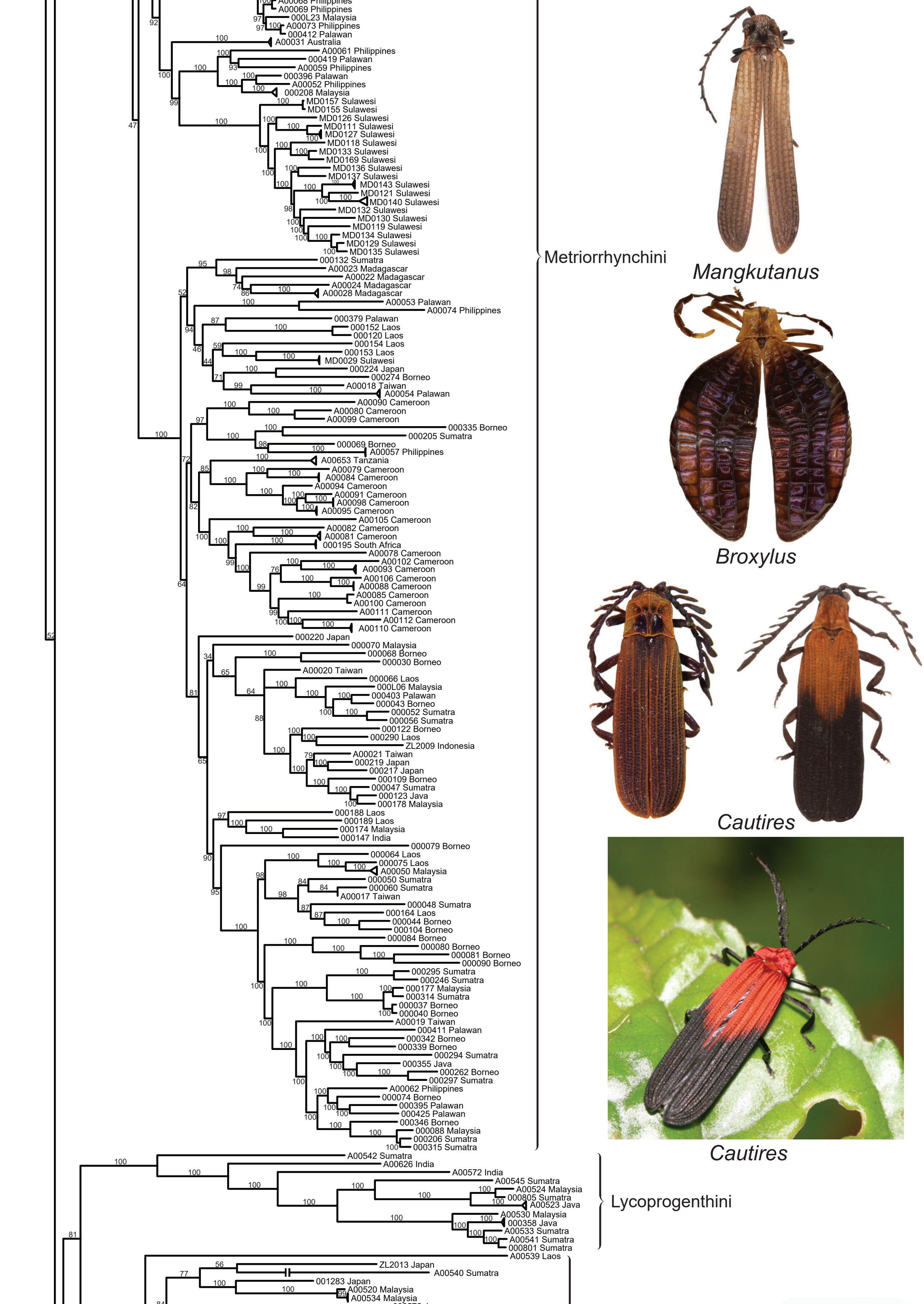

A00068 Philippines
A00069 Philippines
000L23 Malaysia
A00073 Philippines
000412 Palawan
A00031 Australia
A00061 Philippines
000419 Palawan
A00059 Philippines
000396 Palawan
A00052 Philippines
000208 Malaysia
MD0157 Sulawesi
MD0155 Sulawesi
MD0126 Sulawesi
MD0111 Sulawesi
MD0127 Sulawesi
MD0118 Sulawesi
MD0133 Sulawesi
MD0169 Sulawesi
MD0136 Sulawesi
MD0137 Sulawesi
MD0143 Sulawesi
MD0121 Sulawesi
MD0140 Sulawesi
MD0132 Sulawesi
MD0130 Sulawesi
MD0119 Sulawesi
MD0134 Sulawesi
MD0129 Sulawesi
MD0135 Sulawesi
000132 Sumatra
A00023 Madagascar
A00022 Madagascar
A00024 Madagascar
A00028 Madagascar
A00053 Palawan
A00074 Philippines
000379 Palawan
000152 Laos
000120 Laos
000154 Laos
000153 Laos
MD0029 Sulawesi
000224 Japan
000274 Borneo
A00018 Taiwan
A00054 Palawan
A00090 Cameroon
A00080 Cameroon
A00099 Cameroon
000335 Borneo
000205 Sumatra
000069 Borneo
A00057 Philippines
A00653 Tanzania
A00079 Cameroon
A00084 Cameroon
A00094 Cameroon
A00091 Cameroon
A00098 Cameroon
A00095 Cameroon
A00105 Cameroon
A00082 Cameroon
A00081 Cameroon
000195 South Africa
A00078 Cameroon
A00102 Cameroon
A00093 Cameroon
A00106 Cameroon
A00088 Cameroon
A00085 Cameroon
A00100 Cameroon
A00111 Cameroon
A00112 Cameroon
A00110 Cameroon
000220 Japan
000070 Malaysia
000068 Borneo
000030 Borneo
A00020 Taiwan
000066 Laos
000L06 Malaysia
000403 Palawan
000043 Borneo
000052 Sumatra
000056 Sumatra
000122 Borneo
000290 Laos
ZL2009 Indonesia
A00021 Taiwan
000219 Japan
000217 Japan
000109 Borneo
000047 Sumatra
000123 Java
000178 Malaysia
000188 Laos
000189 Laos
000174 Malaysia
000147 India
000079 Borneo
000064 Laos
000075 Laos
A00050 Malaysia
000050 Sumatra
000060 Sumatra
A00017 Taiwan
000048 Sumatra
000164 Laos
000044 Borneo
000104 Borneo
000084 Borneo
000080 Borneo
000081 Borneo
000090 Borneo
000295 Sumatra
000246 Sumatra
000177 Malaysia
000314 Sumatra
000037 Borneo
000040 Borneo
A00019 Taiwan
000411 Palawan
000342 Borneo
000339 Borneo
000294 Sumatra
000355 Java
000262 Borneo
000297 Sumatra
A00062 Philippines
000074 Borneo
000395 Palawan
000425 Palawan
000346 Borneo
000088 Malaysia
000206 Sumatra
000315 Sumatra
Metriorrhynchini
A00542 Sumatra
A00626 India
A00572 India
A00545 Sumatra
A00524 Malaysia
000805 Sumatra
A00523 Java
A00530 Malaysia
000358 Java
A00533 Sumatra
A00541 Sumatra
000801 Sumatra
Lycoprogenthini
A00539 Laos
ZL2013 Japan
A00540 Sumatra
001283 Japan
A00520 Malaysia
A00534 Malaysia
Mangkutanus
Broxylus
Cautires
Cautires

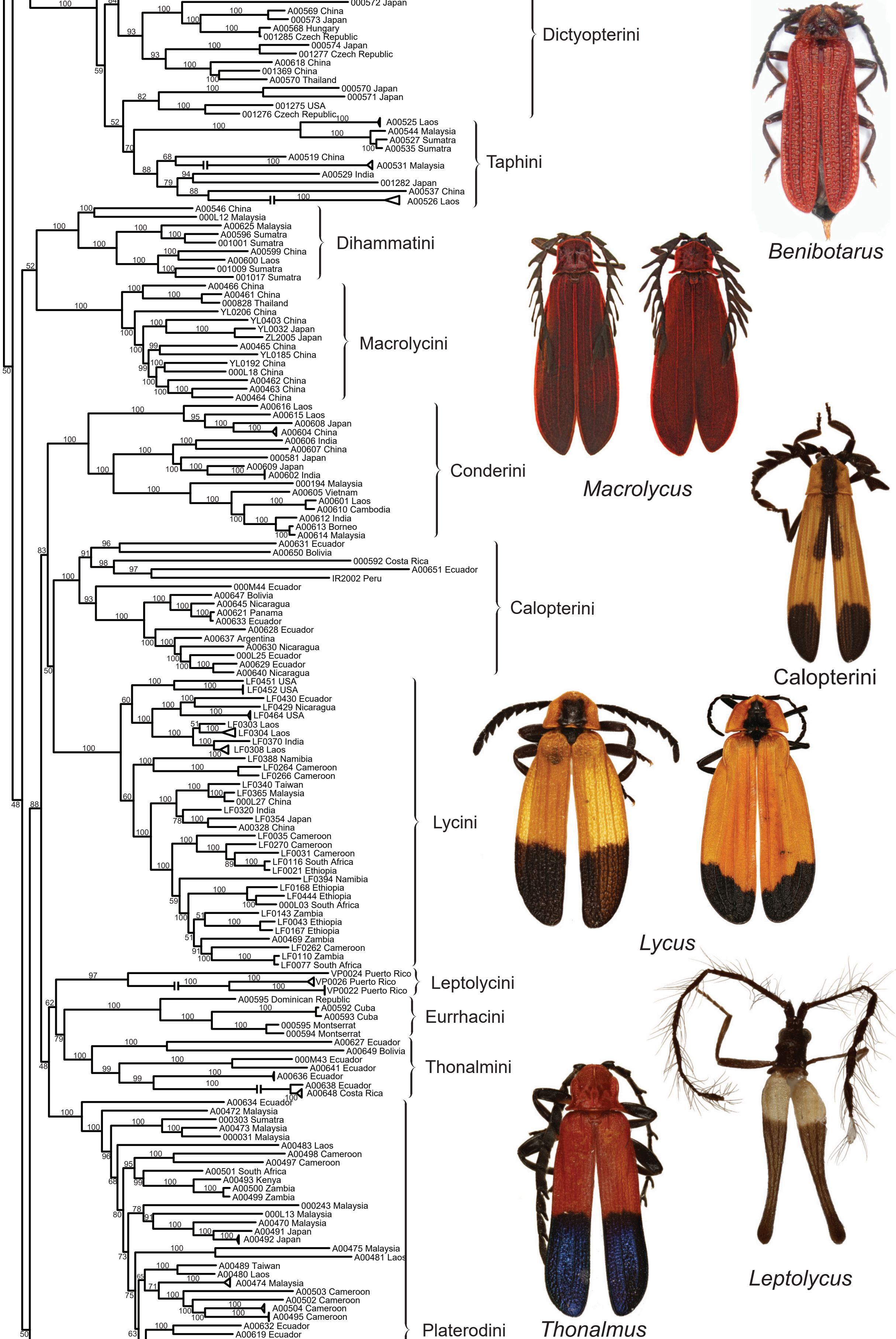
000572 Japan
A00569 China
000573 Japan
A00568 Hungary
001285 Czech Republic
000574 Japan
001277 Czech Republic
A00618 China
001369 China
A00570 Thailand
000570 Japan
000571 Japan
001275 USA
001276 Czech Republic
Dictyopterini
A00525 Laos
A00544 Malaysia
A00527 Sumatra
A00535 Sumatra
A00519 China
A00531 Malaysia
A00529 India
001282 Japan
A00537 China
A00526 Laos
Taphini
A00546 China
000L12 Malaysia
A00625 Malaysia
A00596 Sumatra
001001 Sumatra
A00599 China
A00600 Laos
001009 Sumatra
001017 Sumatra
Dihammatini
A00466 China
A00461 China
000828 Thailand
YL0206 China
YL0403 China
YL0032 Japan
ZL2005 Japan
A00465 China
YL0185 China
YL0192 China
000L18 China
A00462 China
A00463 China
A00464 China
Macrolycini
A00616 Laos
A00615 Laos
A00608 Japan
A00604 China
A00606 India
A00607 China
000581 Japan
A00609 Japan
A00602 India
000194 Malaysia
A00605 Vietnam
A00601 Laos
A00610 Cambodia
A00612 India
A00613 Borneo
A00614 Malaysia
Conderini
A00631 Ecuador
A00650 Bolivia
000592 Costa Rica
A00651 Ecuador
IR2002 Peru
000M44 Ecuador
A00647 Bolivia
A00645 Nicaragua
A00621 Panama
A00633 Ecuador
A00628 Ecuador
A00637 Argentina
A00630 Nicaragua
000L25 Ecuador
A00629 Ecuador
A00640 Nicaragua
Calopterini
LF0451 USA
LF0452 USA
LF0430 Ecuador
LF0429 Nicaragua
LF0464 USA
LF0303 Laos
LF0304 Laos
LF0370 India
LF0308 Laos
LF0388 Namibia
LF0264 Cameroon
LF0266 Cameroon
LF0340 Taiwan
LF0365 Malaysia
000L27 China
LF0320 India
LF0354 Japan
A00328 China
LF0035 Cameroon
LF0270 Cameroon
LF0031 Cameroon
LF0116 South Africa
LF0021 Ethiopia
LF0394 Namibia
LF0168 Ethiopia
LF0444 Ethiopia
000L03 South Africa
LF0143 Zambia
LF0043 Ethiopia
LF0167 Ethiopia
A00469 Zambia
LF0262 Cameroon
LF0110 Zambia
LF0077 South Africa
Lycini
VP0024 Puerto Rico
VP0026 Puerto Rico
VP0022 Puerto Rico
Leptolycini
A00595 Dominican Republic
A00592 Cuba
A00593 Cuba
000595 Montserrat
000594 Montserrat
Eurrhacini
A00627 Ecuador
A00649 Bolivia
000M43 Ecuador
A00641 Ecuador
A00636 Ecuador
A00638 Ecuador
A00648 Costa Rica
Thonalmini
A00634 Ecuador
A00472 Malaysia
000303 Sumatra
A00473 Malaysia
000031 Malaysia
A00483 Laos
A00498 Cameroon
A00497 Cameroon
A00501 South Africa
A00493 Kenya
A00500 Zambia
A00499 Zambia
000243 Malaysia
000L13 Malaysia
A00470 Malaysia
A00491 Japan
A00492 Japan
A00475 Malaysia
A00481 Laos
A00489 Taiwan
A00480 Laos
A00474 Malaysia
A00503 Cameroon
A00502 Cameroon
A00504 Cameroon
A00495 Cameroon
A00632 Ecuador
A00619 Ecuador
Platerodini
Benibotarus
Macrolycus
Calopterini
Lycus
Leptolycus
Thonalmus

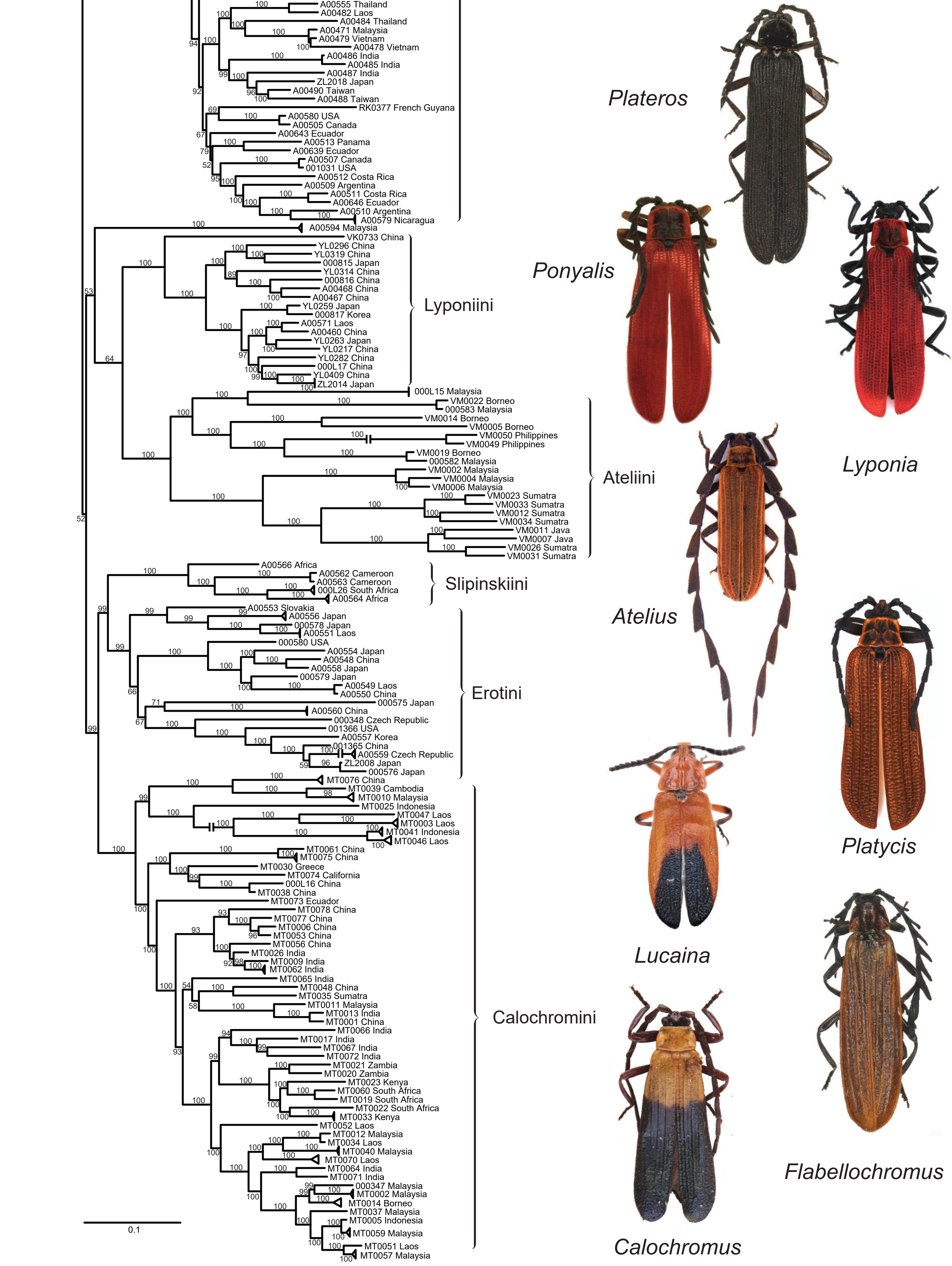

Figure S1. The IQ-tree topology inferred from the MAFFT alignment of all markers and 89 taxa as an outgroup. Species level terminal collapsed.

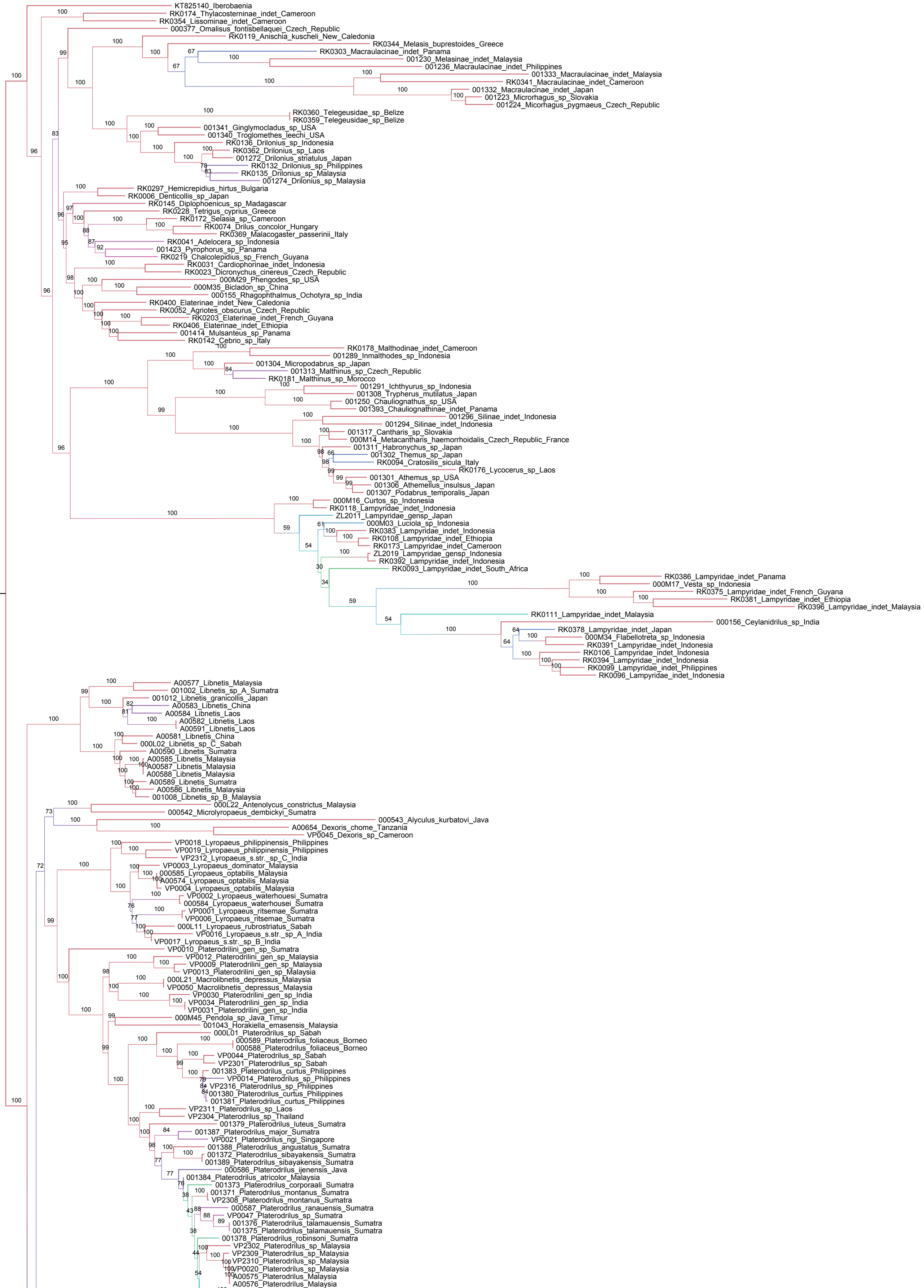
KT825140_Iberobaenia
RK0174_Thylacosterninae_indet_Cameroon
RK0354_Lissominae_indet_Cameroon
000377_Omalisus_fontisbellaquei_Czech_Republic
RK0119_Anischia_kuscheli_New_Caledonia
RK0344_Melasis_buprestoides_Greece
RK0303_Macraulacinae_indet_Panama
001230_Melasinae_indet_Malaysia
001236_Macraulacinae_indet_Philippines
001333_Macraulacinae_indet_Malaysia
RK0341_Macraulacinae_indet_Cameroon
001332_Macraulacinae_indet_Japan
001223_Microrhagus_sp_Slovakia
001224_Micorhagus_pygmaeus_Czech_Republic
RK0360_Telegeusidae_sp_Belize
RK0359_Telegeusidae_sp_Belize
001341_Ginglymocladus_sp_USA
001340_Troglomethes_leechi_USA
RK0136_Drilonius_sp_Indonesia
RK0362_Drilonius_sp_Laos
001272_Drilonius_striatulus_Japan
RK0132_Drilonius_sp_Philippines
RK0135_Drilonius_sp_Malaysia
001274_Drilonius_sp_Malaysia
RK0297_Hemicrepidius_hirtus_Bulgaria
RK0006_Denticollis_sp_Japan
RK0145_Diplophoenicus_sp_Madagascar
RK0228_Tetrigus_cyprius_Greece
RK0172_Selasia_sp_Cameroon
RK0074_Drilus_concolor_Hungary
RK0369_Malacogaster_passerinii_Italy
RK0041_Adelocera_sp_Indonesia
001423_Pyrophorus_sp_Panama
RK0219_Chalcolepidius_sp_French_Guyana
RK0031_Cardiophorinae_indet_Indonesia
RK0023_Dicronychus_cinereus_Czech_Republic
000M29_Phengodes_sp_USA
000M35_Bicladon_sp_China
000155_Rhagophthalmus_Ochotyra_sp_India
RK0400_Elaterinae_indet_New_Caledonia
RK0052_Agriotes_obscurus_Czech_Republic
RK0203_Elaterinae_indet_French_Guyana
RK0406_Elaterinae_indet_Ethiopia
001414_Mulsanteus_sp_Panama
RK0142_Cebrio_sp_Italy
RK0178_Malthodinae_indet_Cameroon
001289_Inmalthodes_sp_Indonesia
001304_Micropodabrus_sp_Japan
001313_Malthinus_sp_Czech_Republic
RK0181_Malthinus_sp_Morocco
001291_Ichthyurus_sp_Indonesia
001308_Trypherus_mutilatus_Japan
001250_Chauliognathus_sp_USA
001393_Chauliognathinae_indet_Panama
001296_Silinae_indet_Indonesia
001294_Silinae_indet_Indonesia
001317_Cantharis_sp_Slovakia
000M14_Metacantharis_haemorrhoidalis_Czech_Republic_France
001311_Habronychus_sp_Japan
001302_Themus_sp_Japan
RK0094_Cratosilis_sicula_Italy
RK0176_Lycocerus_sp_Laos
001301_Athemus_sp_USA
001306_Athemellus_insulsus_Japan
001307_Podabrus_temporalis_Japan
000M16_Curtos_sp_Indonesia
RK0118_Lampyridae_indet_Indonesia
ZL2011_Lampyridae_gensp_Japan
000M03_Luciola_sp_Indonesia
RK0383_Lampyridae_indet_Indonesia
RK0108_Lampyridae_indet_Ethiopia
RK0173_Lampyridae_indet_Cameroon
ZL2019_Lampyridae_gensp_Indonesia
RK0392_Lampyridae_indet_Indonesia
RK0093_Lampyridae_indet_South_Africa
RK0386_Lampyridae_indet_Panama
000M17_Vesta_sp_Indonesia
RK0375_Lampyridae_indet_French_Guyana
RK0381_Lampyridae_indet_Ethiopia
RK0396_Lampyridae_indet_Malaysia
RK0111_Lampyridae_indet_Malaysia
000156_Ceylanidrilus_sp_India
RK0378_Lampyridae_indet_Japan
000M34_Flabellotreta_sp_Indonesia
RK0391_Lampyridae_indet_Indonesia
RK0106_Lampyridae_indet_Indonesia
RK0394_Lampyridae_indet_Indonesia
RK0099_Lampyridae_indet_Philippines
RK0096_Lampyridae_indet_Indonesia
A00577_Libnetis_Malaysia
001002_Libnetis_sp_A_Sumatra
001012_Libnetis_granicollis_Japan
A00583_Libnetis_China
A00584_Libnetis_Laos
A00582_Libnetis_Laos
A00591_Libnetis_Laos
A00581_Libnetis_China
000L02_Libnetis_sp_C_Sabah
A00590_Libnetis_Sumatra
A00585_Libnetis_Malaysia
A00587_Libnetis_Malaysia
A00588_Libnetis_Malaysia
A00589_Libnetis_Sumatra
A00586_Libnetis_Malaysia
001008_Libnetis_sp_B_Malaysia
000L22_Antenolycus_constrictus_Malaysia
000542_Microlyropaeus_dembickyi_Sumatra
000543_Alyculus_kurbatovi_Java
A00654_Dexoris_chome_Tanzania
VP0045_Dexoris_sp_Cameroon
VP0018_Lyropaeus_philippinensis_Philippines
VP0019_Lyropaeus_philippinensis_Philippines
VP2312_Lyropaeus_s.str._sp_C_India
VP0003_Lyropaeus_dominator_Malaysia
000585_Lyropaeus_optabilis_Malaysia
A00574_Lyropaeus_optabilis_Malaysia
VP0004_Lyropaeus_optabilis_Malaysia
VP0002_Lyropaeus_waterhouesi_Sumatra
000584_Lyropaeus_waterhousei_Sumatra
VP0001_Lyropaeus_ritsemae_Sumatra
VP0006_Lyropaeus_ritsemae_Sumatra
000L11_Lyropaeus_rubrostriatus_Sabah
VP0016_Lyropaeus_s.str._sp_A_India
VP0017_Lyropaeus_s.str._sp_B_India
VP0010_Platerodrilini_gen_sp_Sumatra
VP0012_Platerodrilini_gen_sp_Malaysia
VP0009_Platerodrilini_gen_sp_Malaysia
VP0013_Platerodrilini_gen_sp_Malaysia
000L21_Macrolibnetis_depressus_Malaysia
VP0050_Macrolibnetis_depressus_Malaysia
VP0030_Platerodrilini_gen_sp_India
VP0034_Platerodrilini_gen_sp_India
VP0031_Platerodrilini_gen_sp_India
000M45_Pendola_sp_Java_Timur
001043_Horakiella_emasensis_Malaysia
000L01_Platerodrilus_sp_Sabah
000589_Platerodrilus_foliaceus_Borneo
000588_Platerodrilus_foliaceus_Borneo
VP0044_Platerodrilus_sp_Sabah
VP2301_Platerodrilus_sp_Sabah
001383_Platerodrilus_curtus_Philippines
VP0014_Platerodrilus_sp_Philippines
VP2316_Platerodrilus_sp_Philippines
001380_Platerodrilus_curtus_Philippines
001381_Platerodrilus_curtus_Philippines
VP2311_Platerodrilus_sp_Laos
VP2304_Platerodrilus_sp_Thailand
001379_Platerodrilus_luteus_Sumatra
001387_Platerodrilus_major_Sumatra
VP0021_Platerodrilus_ngi_Singapore
001388_Platerodrilus_angustatus_Sumatra
001372_Platerodrilus_sibayakensis_Sumatra
001389_Platerodrilus_sibayakensis_Sumatra
000586_Platerodrilus_ijenensis_Java
001384_Platerodrilus_atricolor_Malaysia
001373_Platerodrilus_corporaali_Sumatra
001371_Platerodrilus_montanus_Sumatra
VP2308_Platerodrilus_montanus_Sumatra
000587_Platerodrilus_ranauensis_Sumatra
VP0047_Platerodrilus_sp_Sumatra
001376_Platerodrilus_talamauensis_Sumatra
001375_Platerodrilus_talamauensis_Sumatra
001378_Platerodrilus_robinsoni_Sumatra
VP2302_Platerodrilus_sp_Malaysia
VP2309_Platerodrilus_sp_Malaysia
VP2310_Platerodrilus_sp_Malaysia
VP0020_Platerodrilus_sp_Malaysia
A00575_Platerodrilus_Malaysia
A00576_Platerodrilus_Malaysia

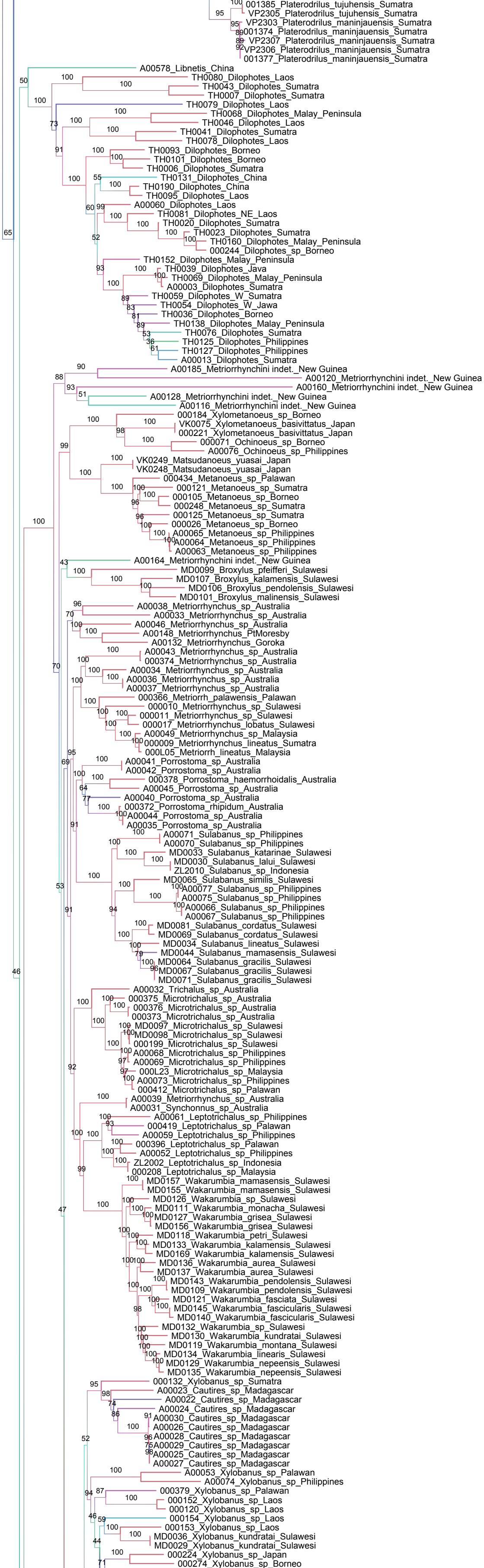
001385_Platerodrilus_tujuhensis_Sumatra
VP2305_Platerodrilus_tujuhensis_Sumatra
VP2303_Platerodrilus_maninjauensis_Sumatra
001374_Platerodrilus_maninjauensis_Sumatra
VP2307_Platerodrilus_maninjauensis_Sumatra
VP2306_Platerodrilus_maninjauensis_Sumatra
001377_Platerodrilus_maninjauensis_Sumatra
A00578_Libnetis_China
TH0080_Dilophotes_Laos
TH0043_Dilophotes_Sumatra
TH0007_Dilophotes_Sumatra
TH0079_Dilophotes_Laos
TH0068_Dilophotes_Malay_Peninsula
TH0046_Dilophotes_Laos
TH0041_Dilophotes_Sumatra
TH0078_Dilophotes_Laos
TH0093_Dilophotes_Borneo
TH0101_Dilophotes_Borneo
TH0006_Dilophotes_Sumatra
TH0131_Dilophotes_China
TH0190_Dilophotes_China
TH0095_Dilophotes_Laos
A00060_Dilophotes_Laos
TH0081_Dilophotes_NE_Laos
TH0020_Dilophotes_Sumatra
TH0023_Dilophotes_Sumatra
TH0160_Dilophotes_Malay_Peninsula
000244_Dilophotes_sp_Borneo
TH0152_Dilophotes_Malay_Peninsula
TH0039_Dilophotes_Java
TH0069_Dilophotes_Malay_Peninsula
A00003_Dilophotes_Sumatra
TH0059_Dilophotes_W_Sumatra
TH0054_Dilophotes_W_Jawa
TH0036_Dilophotes_Borneo
TH0138_Dilophotes_Malay_Peninsula
TH0076_Dilophotes_Sumatra
TH0125_Dilophotes_Philippines
TH0127_Dilophotes_Philippines
A00013_Dilophotes_Sumatra
A00185_Metriorrhynchini indet._New Guinea
A00120_Metriorrhynchini indet._New Guinea
A00160_Metriorrhynchini indet._New Guinea
A00128_Metriorrhynchini indet._New Guinea
A00116_Metriorrhynchini indet._New Guinea
000184_Xylometanoeus_sp_Borneo
VK0075_Xylometanoeus_basivittatus_Japan
000221_Xylometanoeus_basivittatus_Japan
000071_Ochinoeus_sp_Borneo
A00076_Ochinoeus_sp_Philippines
VK0249_Matsudanoeus_yuasai_Japan
VK0248_Matsudanoeus_yuasai_Japan
000434_Metanoeus_sp_Palawan
000121_Metanoeus_sp_Sumatra
000105_Metanoeus_sp_Borneo
000248_Metanoeus_sp_Sumatra
000125_Metanoeus_sp_Sumatra
000026_Metanoeus_sp_Borneo
A00065_Metanoeus_sp_Philippines
A00064_Metanoeus_sp_Philippines
A00063_Metanoeus_sp_Philippines
A00164_Metriorrhynchini indet._New Guinea
MD0099_Broxylus_pfeifferi_Sulawesi
MD0107_Broxylus_kalamensis_Sulawesi
MD0106_Broxylus_pendolensis_Sulawesi
MD0101_Broxylus_malinensis_Sulawesi
A00038_Metriorrhynchus_sp_Australia
A00033_Metriorrhynchus_sp_Australia
A00046_Metriorrhynchus_sp_Australia
A00148_Metriorrhynchus_PtMoresby
A00132_Metriorrhynchus_Goroka
A00043_Metriorrhynchus_sp_Australia
000374_Metriorrhynchus_sp_Australia
A00034_Metriorrhynchus_sp_Australia
A00036_Metriorrhynchus_sp_Australia
A00037_Metriorrhynchus_sp_Australia
000366_Metriorrh_palawensis_Palawan
000010_Metriorrhynchus_sp_Sulawesi
000011_Metriorrhynchus_sp_Sulawesi
000017_Metriorrhynchus_lobatus_Sulawesi
A00049_Metriorrhynchus_sp_Malaysia
000009_Metriorrhynchus_lineatus_Sumatra
000L05_Metriorrh_lineatus_Malaysia
A00041_Porrostoma_sp_Australia
A00042_Porrostoma_sp_Australia
000378_Porrostoma_haemorrhoidalis_Australia
A00045_Porrostoma_sp_Australia
A00040_Porrostoma_sp_Australia
000372_Porrostoma_rhipidum_Australia
A00044_Porrostoma_sp_Australia
A00035_Porrostoma_sp_Australia
A00071_Sulabanus_sp_Philippines
A00070_Sulabanus_sp_Philippines
MD0033_Sulabanus_katarinae_Sulawesi
MD0030_Sulabanus_lalui_Sulawesi
ZL2010_Sulabanus_sp_Indonesia
MD0065_Sulabanus_similis_Sulawesi
A00077_Sulabanus_sp_Philippines
A00075_Sulabanus_sp_Philippines
A00066_Sulabanus_sp_Philippines
A00067_Sulabanus_sp_Philippines
MD0081_Sulabanus_cordatus_Sulawesi
MD0069_Sulabanus_cordatus_Sulawesi
MD0034_Sulabanus_lineatus_Sulawesi
MD0044_Sulabanus_mamasensis_Sulawesi
MD0064_Sulabanus_gracilis_Sulawesi
MD0067_Sulabanus_gracilis_Sulawesi
MD0071_Sulabanus_gracilis_Sulawesi
A00032_Trichalus_sp_Australia
000375_Microtrichalus_sp_Australia
000376_Microtrichalus_sp_Australia
000373_Microtrichalus_sp_Australia
MD0097_Microtrichalus_sp_Sulawesi
MD0098_Microtrichalus_sp_Sulawesi
000199_Microtrichalus_sp_Sulawesi
A00068_Microtrichalus_sp_Philippines
A00069_Microtrichalus_sp_Philippines
000L23_Microtrichalus_sp_Malaysia
A00073_Microtrichalus_sp_Philippines
000412_Microtrichalus_sp_Palawan
A00039_Metriorrhynchus_sp_Australia
A00031_Synchonnus_sp_Australia
A00061_Leptotrichalus_sp_Philippines
000419_Leptotrichalus_sp_Palawan
A00059_Leptotrichalus_sp_Philippines
000396_Leptotrichalus_sp_Palawan
A00052_Leptotrichalus_sp_Philippines
ZL2002_Leptotrichalus_sp_Indonesia
000208_Leptotrichalus_sp_Malaysia
MD0157_Wakarumbia_mamasensis_Sulawesi
MD0155_Wakarumbia_mamasensis_Sulawesi
MD0126_Wakarumbia_sp_Sulawesi
MD0111_Wakarumbia_monacha_Sulawesi
MD0127_Wakarumbia_grisea_Sulawesi
MD0156_Wakarumbia_grisea_Sulawesi
MD0118_Wakarumbia_petri_Sulawesi
MD0133_Wakarumbia_kalamensis_Sulawesi
MD0169_Wakarumbia_kalamensis_Sulawesi
MD0136_Wakarumbia_aurea_Sulawesi
MD0137_Wakarumbia_aurea_Sulawesi
MD0143_Wakarumbia_pendolensis_Sulawesi
MD0109_Wakarumbia_pendolensis_Sulawesi
MD0121_Wakarumbia_fasciata_Sulawesi
MD0145_Wakarumbia_fascicularis_Sulawesi
MD0140_Wakarumbia_fascicularis_Sulawesi
MD0132_Wakarumbia_sp_Sulawesi
MD0130_Wakarumbia_kundratai_Sulawesi
MD0119_Wakarumbia_montana_Sulawesi
MD0134_Wakarumbia_linearis_Sulawesi
MD0129_Wakarumbia_nepeensis_Sulawesi
MD0135_Wakarumbia_nepeensis_Sulawesi
000132_Xylobanus_sp_Sumatra
A00023_Cautires_sp_Madagascar
A00022_Cautires_sp_Madagascar
A00024_Cautires_sp_Madagascar
A00030_Cautires_sp_Madagascar
A00026_Cautires_sp_Madagascar
A00028_Cautires_sp_Madagascar
A00029_Cautires_sp_Madagascar
A00025_Cautires_sp_Madagascar
A00027_Cautires_sp_Madagascar
A00053_Xylobanus_sp_Palawan
A00074_Xylobanus_sp_Philippines
000379_Xylobanus_sp_Palawan
000152_Xylobanus_sp_Laos
000120_Xylobanus_sp_Laos
000154_Xylobanus_sp_Laos
000153_Xylobanus_sp_Laos
MD0036_Xylobanus_kundratai_Sulawesi
MD0029_Xylobanus_kundratai_Sulawesi
000224_Xylobanus_sp_Japan
000274_Xylobanus_sp_Borneo

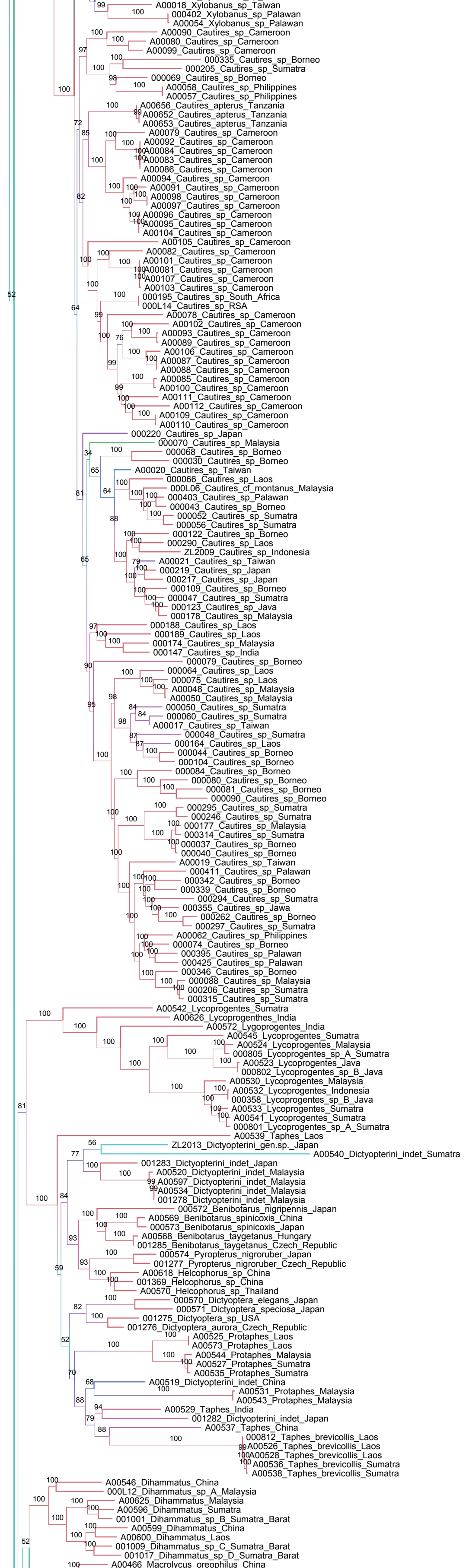
A00018_Xylobanus_sp_Taiwan
000402_Xylobanus_sp_Palawan
A00054_Xylobanus_sp_Palawan
A00090_Cautires_sp_Cameroon
A00080_Cautires_sp_Cameroon
A00099_Cautires_sp_Cameroon
000335_Cautires_sp_Borneo
000205_Cautires_sp_Sumatra
000069_Cautires_sp_Borneo
A00058_Cautires_sp_Philippines
A00057_Cautires_sp_Philippines
A00656_Cautires_apterus_Tanzania
A00652_Cautires_apterus_Tanzania
A00653_Cautires_apterus_Tanzania
A00079_Cautires_sp_Cameroon
A00092_Cautires_sp_Cameroon
A00084_Cautires_sp_Cameroon
A00083_Cautires_sp_Cameroon
A00086_Cautires_sp_Cameroon
A00094_Cautires_sp_Cameroon
A00091_Cautires_sp_Cameroon
A00098_Cautires_sp_Cameroon
A00097_Cautires_sp_Cameroon
A00096_Cautires_sp_Cameroon
A00095_Cautires_sp_Cameroon
A00104_Cautires_sp_Cameroon
A00105_Cautires_sp_Cameroon
A00082_Cautires_sp_Cameroon
A00101_Cautires_sp_Cameroon
A00081_Cautires_sp_Cameroon
A00107_Cautires_sp_Cameroon
A00103_Cautires_sp_Cameroon
000195_Cautires_sp_South_Africa
000L14_Cautires_sp_RSA
A00078_Cautires_sp_Cameroon
A00102_Cautires_sp_Cameroon
A00093_Cautires_sp_Cameroon
A00089_Cautires_sp_Cameroon
A00106_Cautires_sp_Cameroon
A00087_Cautires_sp_Cameroon
A00088_Cautires_sp_Cameroon
A00085_Cautires_sp_Cameroon
A00100_Cautires_sp_Cameroon
A00111_Cautires_sp_Cameroon
A00112_Cautires_sp_Cameroon
A00109_Cautires_sp_Cameroon
A00110_Cautires_sp_Cameroon
000220_Cautires_sp_Japan
000070_Cautires_sp_Malaysia
000068_Cautires_sp_Borneo
000030_Cautires_sp_Borneo
A00020_Cautires_sp_Taiwan
000066_Cautires_sp_Laos
000L06_Cautires_cf_montanus_Malaysia
000403_Cautires_sp_Palawan
000043_Cautires_sp_Borneo
000052_Cautires_sp_Sumatra
000056_Cautires_sp_Sumatra
000122_Cautires_sp_Borneo
000290_Cautires_sp_Laos
ZL2009_Cautires_sp_Indonesia
A00021_Cautires_sp_Taiwan
000219_Cautires_sp_Japan
000217_Cautires_sp_Japan
000109_Cautires_sp_Borneo
000047_Cautires_sp_Sumatra
000123_Cautires_sp_Java
000178_Cautires_sp_Malaysia
000188_Cautires_sp_Laos
000189_Cautires_sp_Laos
000174_Cautires_sp_Malaysia
000147_Cautires_sp_India
000079_Cautires_sp_Borneo
000064_Cautires_sp_Laos
000075_Cautires_sp_Laos
A00048_Cautires_sp_Malaysia
A00050_Cautires_sp_Malaysia
000050_Cautires_sp_Sumatra
000060_Cautires_sp_Sumatra
A00017_Cautires_sp_Taiwan
000048_Cautires_sp_Sumatra
000164_Cautires_sp_Laos
000044_Cautires_sp_Borneo
000104_Cautires_sp_Borneo
000084_Cautires_sp_Borneo
000080_Cautires_sp_Borneo
000081_Cautires_sp_Borneo
000090_Cautires_sp_Borneo
000295_Cautires_sp_Sumatra
000246_Cautires_sp_Sumatra
000177_Cautires_sp_Malaysia
000314_Cautires_sp_Sumatra
000037_Cautires_sp_Borneo
000040_Cautires_sp_Borneo
A00019_Cautires_sp_Taiwan
000411_Cautires_sp_Palawan
000342_Cautires_sp_Borneo
000339_Cautires_sp_Borneo
000294_Cautires_sp_Sumatra
000355_Cautires_sp_Jawa
000262_Cautires_sp_Borneo
000297_Cautires_sp_Sumatra
A00062_Cautires_sp_Philippines
000074_Cautires_sp_Borneo
000395_Cautires_sp_Palawan
000425_Cautires_sp_Palawan
000346_Cautires_sp_Borneo
000088_Cautires_sp_Malaysia
000206_Cautires_sp_Sumatra
000315_Cautires_sp_Sumatra
A00542_Lycoprogentes_Sumatra
A00626_Lycoprogenthes_India
A00572_Lygoprogentes_India
A00545_Lycoprogentes_Sumatra
A00524_Lycoprogentes_Malaysia
000805_Lycoprogentes_sp_A_Sumatra
A00523_Lycoprogentes_Java
000802_Lycoprogentes_sp_B_Java
A00530_Lycoprogentes_Malaysia
A00532_Lycoprogentes_Indonesia
000358_Lycoprogentes_sp_B_Java
A00533_Lycoprogentes_Sumatra
A00541_Lycoprogentes_Sumatra
000801_Lycoprogentes_sp_A_Sumatra
A00539_Taphes_Laos
ZL2013_Dictyopterini_gen.sp._Japan
A00540_Dictyopterini_indet_Sumatra
001283_Dictyopterini_indet_Japan
A00520_Dictyopterini_indet_Malaysia
A00597_Dictyopterini_indet_Malaysia
A00534_Dictyopterini_indet_Malaysia
001278_Dictyopterini_indet_Malaysia
000572_Benibotarus_nigripennis_Japan
A00569_Benibotarus_spinicoxis_China
000573_Benibotarus_spinicoxis_Japan
A00568_Benibotarus_taygetanus_Hungary
001285_Benibotarus_taygetanus_Czech_Republic
000574_Pyropterus_nigroruber_Japan
001277_Pyropterus_nigroruber_Czech_Republic
A00618_Helcophorus_sp_China
001369_Helcophorus_sp_China
A00570_Helcophorus_sp_Thailand
000570_Dictyoptera_elegans_Japan
000571_Dictyoptera_speciosa_Japan
001275_Dictyoptera_sp_USA
001276_Dictyoptera_aurora_Czech_Republic
A00525_Protaphes_Laos
A00573_Protaphes_Laos
A00544_Protaphes_Malaysia
A00527_Protaphes_Sumatra
A00535_Protaphes_Sumatra
A00519_Dictyopterini_indet_China
A00531_Protaphes_Malaysia
A00543_Protaphes_Malaysia
A00529_Taphes_India
001282_Dictyopterini_indet_Japan
A00537_Taphes_China
000812_Taphes_brevicollis_Laos
A00526_Taphes_brevicollis_Laos
A00528_Taphes_brevicollis_Laos
A00536_Taphes_brevicollis_Sumatra
A00538_Taphes_brevicollis_Sumatra
A00546_Dihammatus_China
000L12_Dihammatus_sp_A_Malaysia
A00625_Dihammatus_Malaysia
A00596_Dihammatus_Sumatra
001001_Dihammatus_sp_B_Sumatra_Barat
A00599_Dihammatus_China
A00600_Dihammatus_Laos
001009_Dihammatus_sp_C_Sumatra_Barat
001017_Dihammatus_sp_D_Sumatra_Barat
A00466_Macrolycus_oreophilus_China

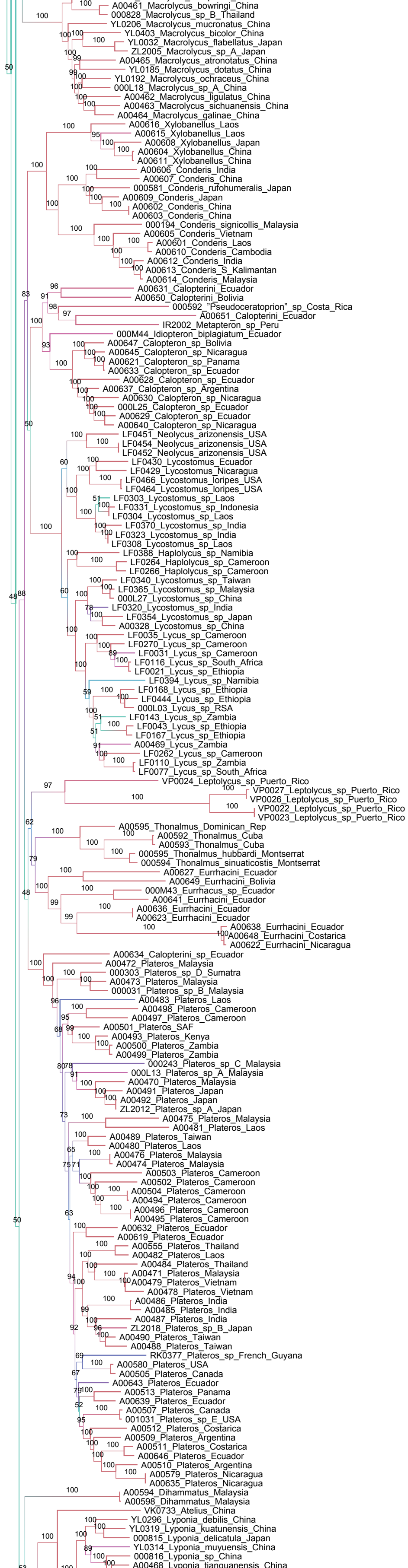
A00461_Macrolycus_bowringi_China
000828_Macrolycus_sp_B_Thailand
YL0206_Macrolycus_mucronatus_China
YL0403_Macrolycus_bicolor_China
YL0032_Macrolycus_flabellatus_Japan
ZL2005_Macrolycus_sp_A_Japan
A00465_Macrolycus_atronotatus_China
YL0185_Macrolycus_dotatus_China
YL0192_Macrolycus_ochraceus_China
000L18_Macrolycus_sp_A_China
A00462_Macrolycus_ligulatus_China
A00463_Macrolycus_sichuanensis_China
A00464_Macrolycus_galinae_China
A00616_Xylobanellus_Laos
A00615_Xylobanellus_Laos
A00608_Xylobanellus_Japan
A00604_Xylobanellus_China
A00611_Xylobanellus_China
A00606_Conderis_India
A00607_Conderis_China
000581_Conderis_rufohumeralis_Japan
A00609_Conderis_Japan
A00602_Conderis_China
A00603_Conderis_China
000194_Conderis_signicollis_Malaysia
A00605_Conderis_Vietnam
A00601_Conderis_Laos
A00610_Conderis_Cambodia
A00612_Conderis_India
A00613_Conderis_S_Kalimantan
A00614_Conderis_Malaysia
A00631_Calopterini_Ecuador
A00650_Calopterini_Bolivia
000592_"Pseudoceratoprion"_sp_Costa_Rica
A00651_Calopterini_Ecuador
IR2002_Metapteron_sp_Peru
000M44_Idiopteron_biplagiatum_Ecuador
A00647_Calopteron_sp_Bolivia
A00645_Calopteron_sp_Nicaragua
A00621_Calopteron_sp_Panama
A00633_Calopteron_sp_Ecuador
A00628_Calopteron_sp_Ecuador
A00637_Calopteron_sp_Argentina
A00630_Calopteron_sp_Nicaragua
000L25_Calopteron_sp_Ecuador
A00629_Calopteron_sp_Ecuador
A00640_Calopteron_sp_Nicaragua
LF0451_Neolycus_arizonensis_USA
LF0454_Neolycus_arizonensis_USA
LF0452_Neolycus_arizonensis_USA
LF0430_Lycostomus_Ecuador
LF0429_Lycostomus_Nicaragua
LF0466_Lycostomus_loripes_USA
LF0464_Lycostomus_loripes_USA
LF0303_Lycostomus_sp_Laos
LF0331_Lycostomus_sp_Indonesia
LF0304_Lycostomus_sp_Laos
LF0370_Lycostomus_sp_India
LF0323_Lycostomus_sp_India
LF0308_Lycostomus_sp_Laos
LF0388_Haplolycus_sp_Namibia
LF0264_Haplolycus_sp_Cameroon
LF0266_Haplolycus_sp_Cameroon
LF0340_Lycostomus_sp_Taiwan
LF0365_Lycostomus_sp_Malaysia
000L27_Lycostomus_sp_China
LF0320_Lycostomus_sp_India
LF0354_Lycostomus_sp_Japan
A00328_Lycostomus_sp_China
LF0035_Lycus_sp_Cameroon
LF0270_Lycus_sp_Cameroon
LF0031_Lycus_sp_Cameroon
LF0116_Lycus_sp_South_Africa
LF0021_Lycus_sp_Ethiopia
LF0394_Lycus_sp_Namibia
LF0168_Lycus_sp_Ethiopia
LF0444_Lycus_sp_Ethiopia
000L03_Lycus_sp_RSA
LF0143_Lycus_sp_Zambia
LF0043_Lycus_sp_Ethiopia
LF0167_Lycus_sp_Ethiopia
A00469_Lycus_Zambia
LF0262_Lycus_sp_Cameroon
LF0110_Lycus_sp_Zambia
LF0077_Lycus_sp_South_Africa
VP0024_Leptolycus_sp_Puerto_Rico
VP0027_Leptolycus_sp_Puerto_Rico
VP0026_Leptolycus_sp_Puerto_Rico
VP0022_Leptolycus_sp_Puerto_Rico
VP0023_Leptolycus_sp_Puerto_Rico
A00595_Thonalmus_Dominican_Rep
A00592_Thonalmus_Cuba
A00593_Thonalmus_Cuba
000595_Thonalmus_hubbardi_Montserrat
000594_Thonalmus_sinuaticostis_Montserrat
A00627_Eurrhacini_Ecuador
A00649_Eurrhacini_Bolivia
000M43_Eurrhacus_sp_Ecuador
A00641_Eurrhacini_Ecuador
A00636_Eurrhacini_Ecuador
A00623_Eurrhacini_Ecuador
A00638_Eurrhacini_Ecuador
A00648_Eurrhacini_Costarica
A00622_Eurrhacini_Nicaragua
A00634_Calopterini_sp_Ecuador
A00472_Plateros_Malaysia
000303_Plateros_sp_D_Sumatra
A00473_Plateros_Malaysia
000031_Plateros_sp_B_Malaysia
A00483_Plateros_Laos
A00498_Plateros_Cameroon
A00497_Plateros_Cameroon
A00501_Plateros_SAF
A00493_Plateros_Kenya
A00500_Plateros_Zambia
A00499_Plateros_Zambia
000243_Plateros_sp_C_Malaysia
000L13_Plateros_sp_A_Malaysia
A00470_Plateros_Malaysia
A00491_Plateros_Japan
A00492_Plateros_Japan
ZL2012_Plateros_sp_A_Japan
A00475_Plateros_Malaysia
A00481_Plateros_Laos
A00489_Plateros_Taiwan
A00480_Plateros_Laos
A00476_Plateros_Malaysia
A00474_Plateros_Malaysia
A00503_Plateros_Cameroon
A00502_Plateros_Cameroon
A00504_Plateros_Cameroon
A00494_Plateros_Cameroon
A00496_Plateros_Cameroon
A00495_Plateros_Cameroon
A00632_Plateros_Ecuador
A00619_Plateros_Ecuador
A00555_Plateros_Thailand
A00482_Plateros_Laos
A00484_Plateros_Thailand
A00471_Plateros_Malaysia
A00479_Plateros_Vietnam
A00478_Plateros_Vietnam
A00486_Plateros_India
A00485_Plateros_India
A00487_Plateros_India
ZL2018_Plateros_sp_B_Japan
A00490_Plateros_Taiwan
A00488_Plateros_Taiwan
RK0377_Plateros_sp_French_Guyana
A00580_Plateros_USA
A00505_Plateros_Canada
A00643_Plateros_Ecuador
A00513_Plateros_Panama
A00639_Plateros_Ecuador
A00507_Plateros_Canada
001031_Plateros_sp_E_USA
A00512_Plateros_Costarica
A00509_Plateros_Argentina
A00511_Plateros_Costarica
A00646_Plateros_Ecuador
A00510_Plateros_Argentina
A00579_Plateros_Nicaragua
A00635_Plateros_Nicaragua
A00594_Dihammatus_Malaysia
A00598_Dihammatus_Malaysia
VK0733_Atelius_China
YL0296_Lyponia_debilis_China
YL0319_Lyponia_kuatunensis_China
000815_Lyponia_delicatula_Japan
YL0314_Lyponia_muyuensis_China
000816_Lyponia_sp_China
A00468_Lyponia_tianguanensis_China

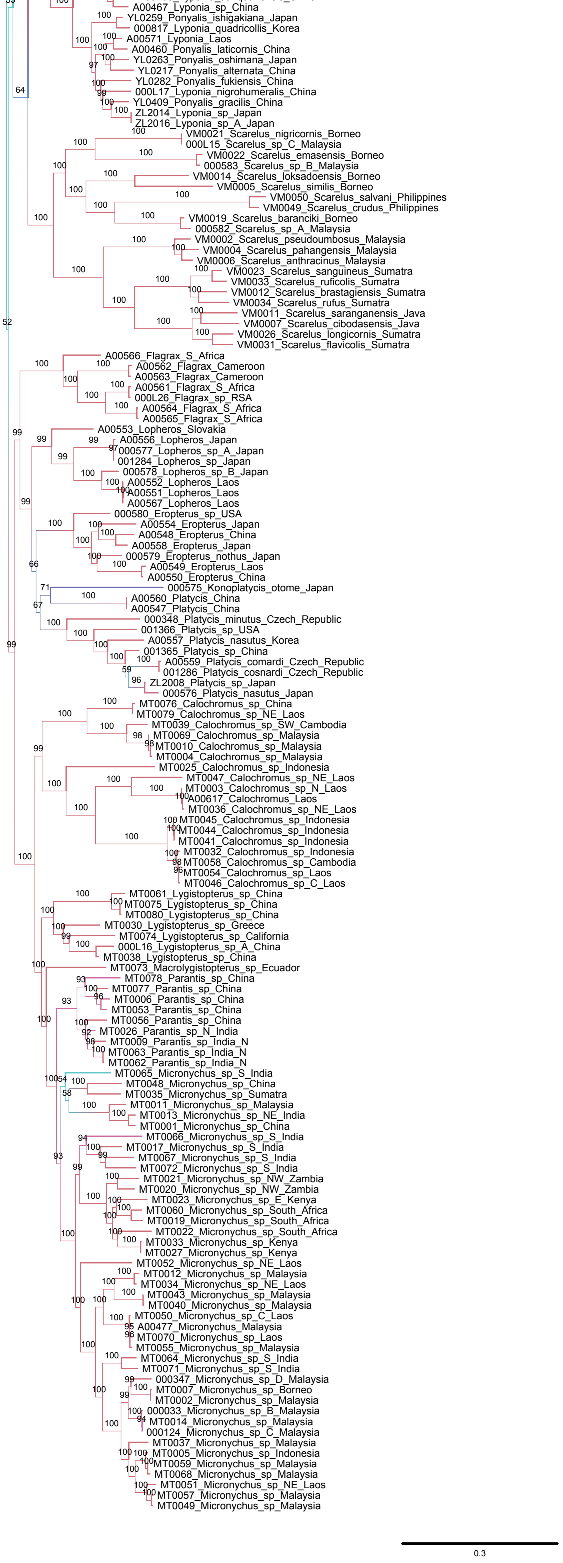

Figure S2. The IQ-tree topology inferred from the MAFFT alignment of all markers and 89 taxa as an outgroup. All taxa represented, full resolution three of net-winged beetles.

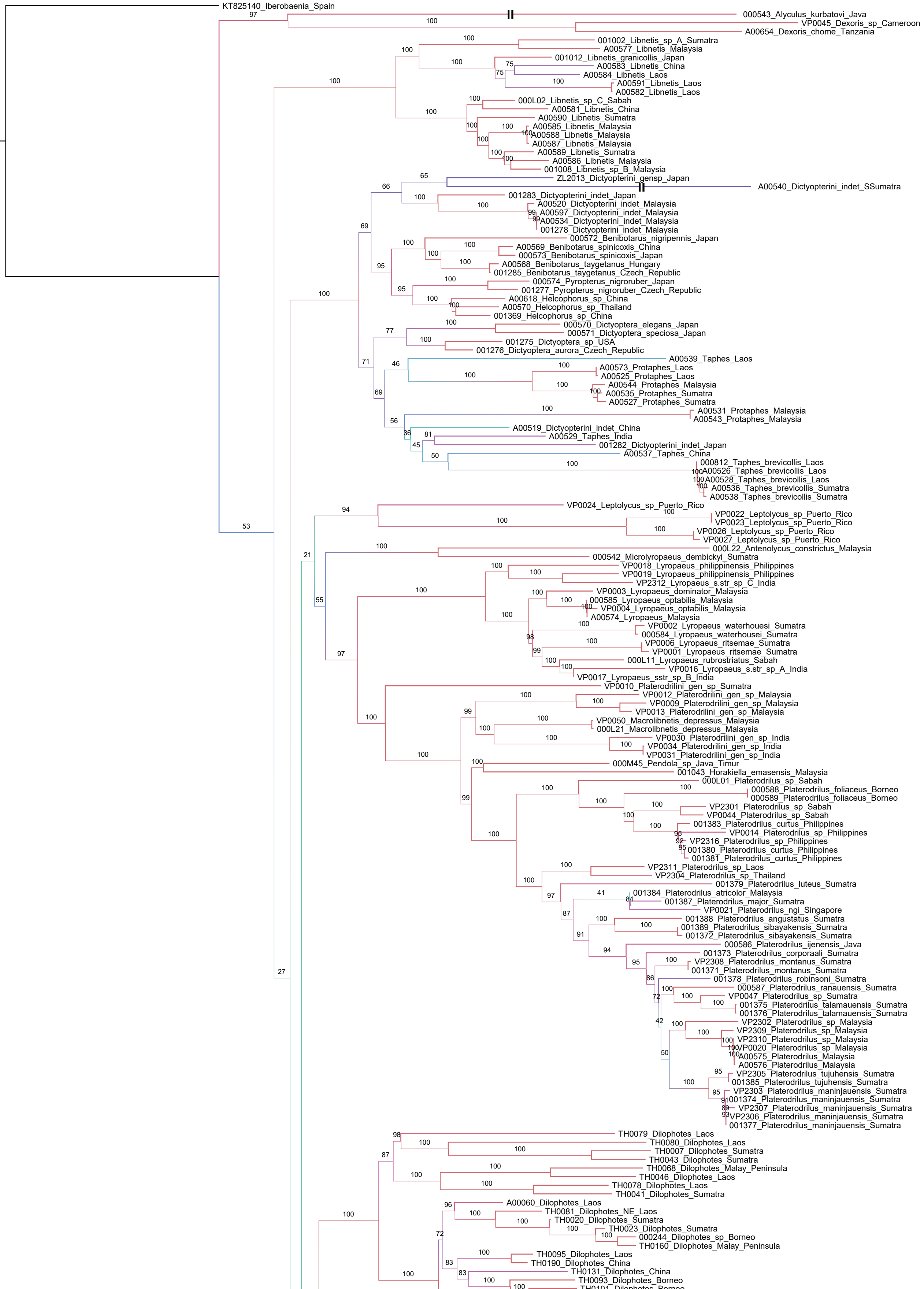
KT825140_Iberobaenia_Spain
000543_Alyculus_kurbatovi_Java
VP0045_Dexoris_sp_Cameroon
A00654_Dexoris_chome_Tanzania
001002_Libnetis_sp_A_Sumatra
A00577_Libnetis_Malaysia
001012_Libnetis_granicollis_Japan
A00583_Libnetis_China
A00584_Libnetis_Laos
A00591_Libnetis_Laos
A00582_Libnetis_Laos
000L02_Libnetis_sp_C_Sabah
A00581_Libnetis_China
A00590_Libnetis_Sumatra
A00585_Libnetis_Malaysia
A00588_Libnetis_Malaysia
A00587_Libnetis_Malaysia
A00589_Libnetis_Sumatra
A00586_Libnetis_Malaysia
001008_Libnetis_sp_B_Malaysia
ZL2013_Dictyopterini_gensp_Japan
A00540_Dictyopterini_indet_SSumatra
001283_Dictyopterini_indet_Japan
A00520_Dictyopterini_indet_Malaysia
A00597_Dictyopterini_indet_Malaysia
A00534_Dictyopterini_indet_Malaysia
001278_Dictyopterini_indet_Malaysia
000572_Benibotarus_nigripennis_Japan
A00569_Benibotarus_spinicoxis_China
000573_Benibotarus_spinicoxis_Japan
A00568_Benibotarus_taygetanus_Hungary
001285_Benibotarus_taygetanus_Czech_Republic
000574_Pyropterus_nigroruber_Japan
001277_Pyropterus_nigroruber_Czech_Republic
A00618_Helcophorus_sp_China
A00570_Helcophorus_sp_Thailand
001369_Helcophorus_sp_China
000570_Dictyoptera_elegans_Japan
000571_Dictyoptera_speciosa_Japan
001275_Dictyoptera_sp_USA
001276_Dictyoptera_aurora_Czech_Republic
A00539_Taphes_Laos
A00573_Protaphes_Laos
A00525_Protaphes_Laos
A00544_Protaphes_Malaysia
A00535_Protaphes_Sumatra
A00527_Protaphes_Sumatra
A00531_Protaphes_Malaysia
A00543_Protaphes_Malaysia
A00519_Dictyopterini_indet_China
A00529_Taphes_India
001282_Dictyopterini_indet_Japan
A00537_Taphes_China
000812_Taphes_brevicollis_Laos
A00526_Taphes_brevicollis_Laos
A00528_Taphes_brevicollis_Laos
A00536_Taphes_brevicollis_Sumatra
A00538_Taphes_brevicollis_Sumatra
VP0024_Leptolycus_sp_Puerto_Rico
VP0022_Leptolycus_sp_Puerto_Rico
VP0023_Leptolycus_sp_Puerto_Rico
VP0026_Leptolycus_sp_Puerto_Rico
VP0027_Leptolycus_sp_Puerto_Rico
000L22_Antenolycus_constrictus_Malaysia
000542_Microlyropaeus_dembickyi_Sumatra
VP0018_Lyropaeus_philippinensis_Philippines
VP0019_Lyropaeus_philippinensis_Philippines
VP2312_Lyropaeus_s.str_sp_C_India
VP0003_Lyropaeus_dominator_Malaysia
000585_Lyropaeus_optabilis_Malaysia
VP0004_Lyropaeus_optabilis_Malaysia
A00574_Lyropaeus_Malaysia
VP0002_Lyropaeus_waterhouesi_Sumatra
000584_Lyropaeus_waterhousei_Sumatra
VP0006_Lyropaeus_ritsemae_Sumatra
VP0001_Lyropaeus_ritsemae_Sumatra
000L11_Lyropaeus_rubrostriatus_Sabah
VP0016_Lyropaeus_s.str_sp_A_India
VP0017_Lyropaeus_sstr_sp_B_India
VP0010_Platerodrilini_gen_sp_Sumatra
VP0012_Platerodrilini_gen_sp_Malaysia
VP0009_Platerodrilini_gen_sp_Malaysia
VP0013_Platerodrilini_gen_sp_Malaysia
VP0050_Macrolibnetis_depressus_Malaysia
000L21_Macrolibnetis_depressus_Malaysia
VP0030_Platerodrilini_gen_sp_India
VP0034_Platerodrilini_gen_sp_India
VP0031_Platerodrilini_gen_sp_India
000M45_Pendola_sp_Java_Timur
001043_Horakiella_emasensis_Malaysia
000L01_Platerodrilus_sp_Sabah
000588_Platerodrilus_foliaceus_Borneo
000589_Platerodrilus_foliaceus_Borneo
VP2301_Platerodrilus_sp_Sabah
VP0044_Platerodrilus_sp_Sabah
001383_Platerodrilus_curtus_Philippines
VP0014_Platerodrilus_sp_Philippines
VP2316_Platerodrilus_sp_Philippines
001380_Platerodrilus_curtus_Philippines
001381_Platerodrilus_curtus_Philippines
VP2311_Platerodrilus_sp_Laos
VP2304_Platerodrilus_sp_Thailand
001379_Platerodrilus_luteus_Sumatra
001384_Platerodrilus_atricolor_Malaysia
001387_Platerodrilus_major_Sumatra
VP0021_Platerodrilus_ngi_Singapore
001388_Platerodrilus_angustatus_Sumatra
001389_Platerodrilus_sibayakensis_Sumatra
001372_Platerodrilus_sibayakensis_Sumatra
000586_Platerodrilus_ijenensis_Java
001373_Platerodrilus_corporaali_Sumatra
VP2308_Platerodrilus_montanus_Sumatra
001371_Platerodrilus_montanus_Sumatra
001378_Platerodrilus_robinsoni_Sumatra
000587_Platerodrilus_ranauensis_Sumatra
VP0047_Platerodrilus_sp_Sumatra
001375_Platerodrilus_talamauensis_Sumatra
001376_Platerodrilus_talamauensis_Sumatra
VP2302_Platerodrilus_sp_Malaysia
VP2309_Platerodrilus_sp_Malaysia
VP2310_Platerodrilus_sp_Malaysia
VP0020_Platerodrilus_sp_Malaysia
A00575_Platerodrilus_Malaysia
A00576_Platerodrilus_Malaysia
VP2305_Platerodrilus_tujuhensis_Sumatra
001385_Platerodrilus_tujuhensis_Sumatra
VP2303_Platerodrilus_maninjauensis_Sumatra
001374_Platerodrilus_maninjauensis_Sumatra
VP2307_Platerodrilus_maninjauensis_Sumatra
VP2306_Platerodrilus_maninjauensis_Sumatra
001377_Platerodrilus_maninjauensis_Sumatra
TH0079_Dilophotes_Laos
TH0080_Dilophotes_Laos
TH0007_Dilophotes_Sumatra
TH0043_Dilophotes_Sumatra
TH0068_Dilophotes_Malay_Peninsula
TH0046_Dilophotes_Laos
TH0078_Dilophotes_Laos
TH0041_Dilophotes_Sumatra
A00060_Dilophotes_Laos
TH0081_Dilophotes_NE_Laos
TH0020_Dilophotes_Sumatra
TH0023_Dilophotes_Sumatra
000244_Dilophotes_sp_Borneo
TH0160_Dilophotes_Malay_Peninsula
TH0095_Dilophotes_Laos
TH0190_Dilophotes_China
TH0131_Dilophotes_China
TH0093_Dilophotes_Borneo

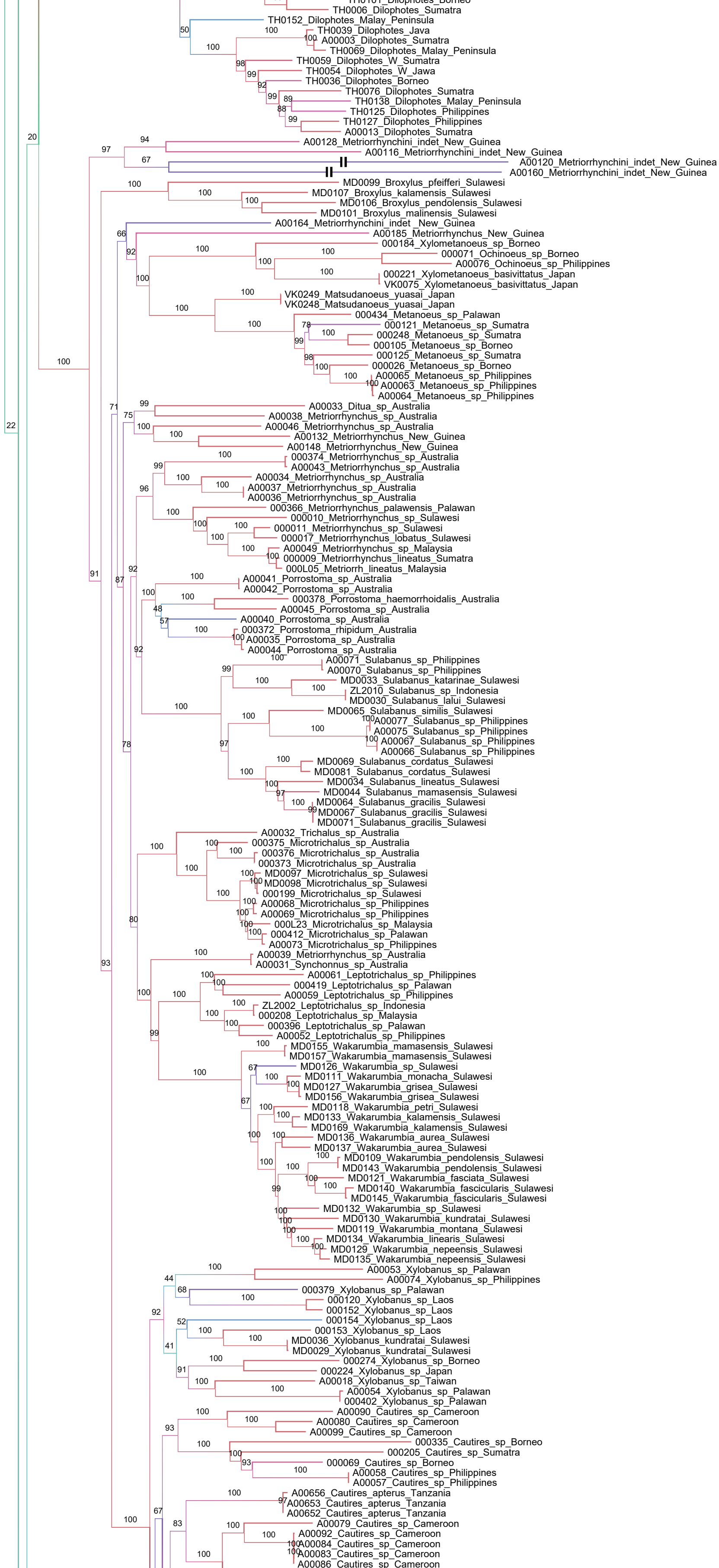
TH0006_Dilophotes_Sumatra
TH0152_Dilophotes_Malay_Peninsula
TH0039_Dilophotes_Java
A00003_Dilophotes_Sumatra
TH0069_Dilophotes_Malay_Peninsula
TH0059_Dilophotes_W_Sumatra
TH0054_Dilophotes_W_Jawa
TH0036_Dilophotes_Borneo
TH0076_Dilophotes_Sumatra
TH0138_Dilophotes_Malay_Peninsula
TH0125_Dilophotes_Philippines
TH0127_Dilophotes_Philippines
A00013_Dilophotes_Sumatra
A00128_Metriorrhynchini_indet_New_Guinea
A00116_Metriorrhynchini_indet_New_Guinea
A00120_Metriorrhynchini_indet_New_Guinea
A00160_Metriorrhynchini_indet_New_Guinea
MD0099_Broxylus_pfeifferi_Sulawesi
MD0107_Broxylus_kalamensis_Sulawesi
MD0106_Broxylus_pendolensis_Sulawesi
MD0101_Broxylus_malinensis_Sulawesi
A00164_Metriorrhynchini_indet _New_Guinea
A00185_Metriorrhynchus_New_Guinea
000184_Xylometanoeus_sp_Borneo
000071_Ochinoeus_sp_Borneo
A00076_Ochinoeus_sp_Philippines
000221_Xylometanoeus_basivittatus_Japan
VK0075_Xylometanoeus_basivittatus_Japan
VK0249_Matsudanoeus_yuasai_Japan
VK0248_Matsudanoeus_yuasai_Japan
000434_Metanoeus_sp_Palawan
000121_Metanoeus_sp_Sumatra
000248_Metanoeus_sp_Sumatra
000105_Metanoeus_sp_Borneo
000125_Metanoeus_sp_Sumatra
000026_Metanoeus_sp_Borneo
A00065_Metanoeus_sp_Philippines
A00063_Metanoeus_sp_Philippines
A00064_Metanoeus_sp_Philippines
A00033_Ditua_sp_Australia
A00038_Metriorrhynchus_sp_Australia
A00046_Metriorrhynchus_sp_Australia
A00132_Metriorrhynchus_New_Guinea
A00148_Metriorrhynchus_New_Guinea
000374_Metriorrhynchus_sp_Australia
A00043_Metriorrhynchus_sp_Australia
A00034_Metriorrhynchus_sp_Australia
A00037_Metriorrhynchus_sp_Australia
A00036_Metriorrhynchus_sp_Australia
000366_Metriorrhynchus_palawensis_Palawan
000010_Metriorrhynchus_sp_Sulawesi
000011_Metriorrhynchus_sp_Sulawesi
000017_Metriorrhynchus_lobatus_Sulawesi
A00049_Metriorrhynchus_sp_Malaysia
000009_Metriorrhynchus_lineatus_Sumatra
000L05_Metriorrh_lineatus_Malaysia
A00041_Porrostoma_sp_Australia
A00042_Porrostoma_sp_Australia
000378_Porrostoma_haemorrhoidalis_Australia
A00045_Porrostoma_sp_Australia
A00040_Porrostoma_sp_Australia
000372_Porrostoma_rhipidum_Australia
A00035_Porrostoma_sp_Australia
A00044_Porrostoma_sp_Australia
A00071_Sulabanus_sp_Philippines
A00070_Sulabanus_sp_Philippines
MD0033_Sulabanus_katarinae_Sulawesi
ZL2010_Sulabanus_sp_Indonesia
MD0030_Sulabanus_lalui_Sulawesi
MD0065_Sulabanus_similis_Sulawesi
A00077_Sulabanus_sp_Philippines
A00075_Sulabanus_sp_Philippines
A00067_Sulabanus_sp_Philippines
A00066_Sulabanus_sp_Philippines
MD0069_Sulabanus_cordatus_Sulawesi
MD0081_Sulabanus_cordatus_Sulawesi
MD0034_Sulabanus_lineatus_Sulawesi
MD0044_Sulabanus_mamasensis_Sulawesi
MD0064_Sulabanus_gracilis_Sulawesi
MD0067_Sulabanus_gracilis_Sulawesi
MD0071_Sulabanus_gracilis_Sulawesi
A00032_Trichalus_sp_Australia
000375_Microtrichalus_sp_Australia
000376_Microtrichalus_sp_Australia
000373_Microtrichalus_sp_Australia
MD0097_Microtrichalus_sp_Sulawesi
MD0098_Microtrichalus_sp_Sulawesi
000199_Microtrichalus_sp_Sulawesi
A00068_Microtrichalus_sp_Philippines
A00069_Microtrichalus_sp_Philippines
000L23_Microtrichalus_sp_Malaysia
000412_Microtrichalus_sp_Palawan
A00073_Microtrichalus_sp_Philippines
A00039_Metriorrhynchus_sp_Australia
A00031_Synchonnus_sp_Australia
A00061_Leptotrichalus_sp_Philippines
000419_Leptotrichalus_sp_Palawan
A00059_Leptotrichalus_sp_Philippines
ZL2002_Leptotrichalus_sp_Indonesia
000208_Leptotrichalus_sp_Malaysia
000396_Leptotrichalus_sp_Palawan
A00052_Leptotrichalus_sp_Philippines
MD0155_Wakarumbia_mamasensis_Sulawesi
MD0157_Wakarumbia_mamasensis_Sulawesi
MD0126_Wakarumbia_sp_Sulawesi
MD0111_Wakarumbia_monacha_Sulawesi
MD0127_Wakarumbia_grisea_Sulawesi
MD0156_Wakarumbia_grisea_Sulawesi
MD0118_Wakarumbia_petri_Sulawesi
MD0133_Wakarumbia_kalamensis_Sulawesi
MD0169_Wakarumbia_kalamensis_Sulawesi
MD0136_Wakarumbia_aurea_Sulawesi
MD0137_Wakarumbia_aurea_Sulawesi
MD0109_Wakarumbia_pendolensis_Sulawesi
MD0143_Wakarumbia_pendolensis_Sulawesi
MD0121_Wakarumbia_fasciata_Sulawesi
MD0140_Wakarumbia_fascicularis_Sulawesi
MD0145_Wakarumbia_fascicularis_Sulawesi
MD0132_Wakarumbia_sp_Sulawesi
MD0130_Wakarumbia_kundratai_Sulawesi
MD0119_Wakarumbia_montana_Sulawesi
MD0134_Wakarumbia_linearis_Sulawesi
MD0129_Wakarumbia_nepeensis_Sulawesi
MD0135_Wakarumbia_nepeensis_Sulawesi
A00053_Xylobanus_sp_Palawan
A00074_Xylobanus_sp_Philippines
000379_Xylobanus_sp_Palawan
000120_Xylobanus_sp_Laos
000152_Xylobanus_sp_Laos
000154_Xylobanus_sp_Laos
000153_Xylobanus_sp_Laos
MD0036_Xylobanus_kundratai_Sulawesi
MD0029_Xylobanus_kundratai_Sulawesi
000274_Xylobanus_sp_Borneo
000224_Xylobanus_sp_Japan
A00018_Xylobanus_sp_Taiwan
A00054_Xylobanus_sp_Palawan
000402_Xylobanus_sp_Palawan
A00090_Cautires_sp_Cameroon
A00080_Cautires_sp_Cameroon
A00099_Cautires_sp_Cameroon
000335_Cautires_sp_Borneo
000205_Cautires_sp_Sumatra
000069_Cautires_sp_Borneo
A00058_Cautires_sp_Philippines
A00057_Cautires_sp_Philippines
A00656_Cautires_apterus_Tanzania
A00653_Cautires_apterus_Tanzania
A00652_Cautires_apterus_Tanzania
A00079_Cautires_sp_Cameroon
A00092_Cautires_sp_Cameroon
A00084_Cautires_sp_Cameroon
A00083_Cautires_sp_Cameroon
A00086_Cautires_sp_Cameroon

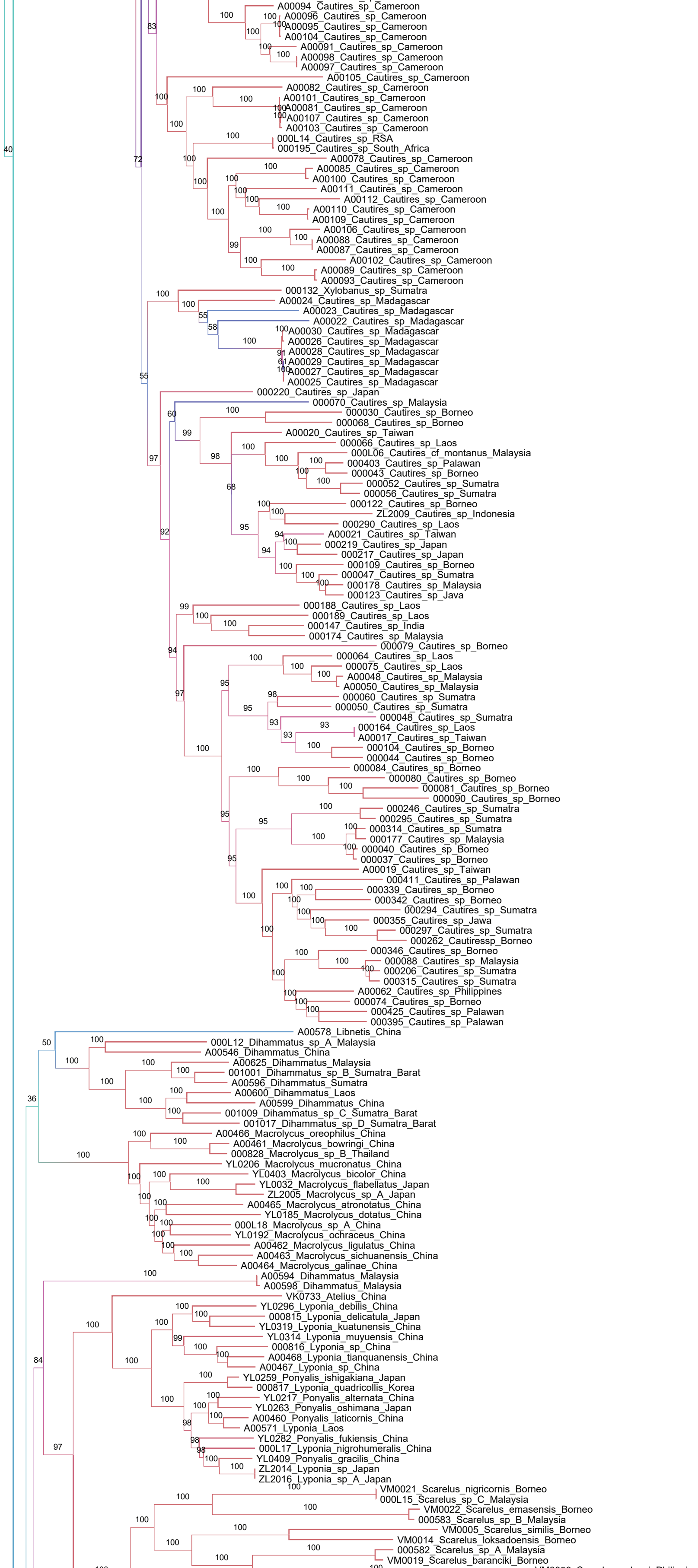
A00094_Cautires_sp_Cameroon
A00096_Cautires_sp_Cameroon
A00095_Cautires_sp_Cameroon
A00104_Cautires_sp_Cameroon
A00091_Cautires_sp_Cameroon
A00098_Cautires_sp_Cameroon
A00097_Cautires_sp_Cameroon
A00105_Cautires_sp_Cameroon
A00082_Cautires_sp_Cameroon
A00101_Cautires_sp_Cameroon
A00081_Cautires_sp_Cameroon
A00107_Cautires_sp_Cameroon
A00103_Cautires_sp_Cameroon
000L14_Cautires_sp_RSA
000195_Cautires_sp_South_Africa
A00078_Cautires_sp_Cameroon
A00085_Cautires_sp_Cameroon
A00100_Cautires_sp_Cameroon
A00111_Cautires_sp_Cameroon
A00112_Cautires_sp_Cameroon
A00110_Cautires_sp_Cameroon
A00109_Cautires_sp_Cameroon
A00106_Cautires_sp_Cameroon
A00088_Cautires_sp_Cameroon
A00087_Cautires_sp_Cameroon
A00102_Cautires_sp_Cameroon
A00089_Cautires_sp_Cameroon
A00093_Cautires_sp_Cameroon
000132_Xylobanus_sp_Sumatra
A00024_Cautires_sp_Madagascar
A00023_Cautires_sp_Madagascar
A00022_Cautires_sp_Madagascar
A00030_Cautires_sp_Madagascar
A00026_Cautires_sp_Madagascar
A00028_Cautires_sp_Madagascar
A00029_Cautires_sp_Madagascar
A00027_Cautires_sp_Madagascar
A00025_Cautires_sp_Madagascar
000220_Cautires_sp_Japan
000070_Cautires_sp_Malaysia
000030_Cautires_sp_Borneo
000068_Cautires_sp_Borneo
A00020_Cautires_sp_Taiwan
000066_Cautires_sp_Laos
000L06_Cautires_cf_montanus_Malaysia
000403_Cautires_sp_Palawan
000043_Cautires_sp_Borneo
000052_Cautires_sp_Sumatra
000056_Cautires_sp_Sumatra
000122_Cautires_sp_Borneo
ZL2009_Cautires_sp_Indonesia
000290_Cautires_sp_Laos
A00021_Cautires_sp_Taiwan
000219_Cautires_sp_Japan
000217_Cautires_sp_Japan
000109_Cautires_sp_Borneo
000047_Cautires_sp_Sumatra
000178_Cautires_sp_Malaysia
000123_Cautires_sp_Java
000188_Cautires_sp_Laos
000189_Cautires_sp_Laos
000147_Cautires_sp_India
000174_Cautires_sp_Malaysia
000079_Cautires_sp_Borneo
000064_Cautires_sp_Laos
000075_Cautires_sp_Laos
A00048_Cautires_sp_Malaysia
A00050_Cautires_sp_Malaysia
000060_Cautires_sp_Sumatra
000050_Cautires_sp_Sumatra
000048_Cautires_sp_Sumatra
000164_Cautires_sp_Laos
A00017_Cautires_sp_Taiwan
000104_Cautires_sp_Borneo
000044_Cautires_sp_Borneo
000084_Cautires_sp_Borneo
000080_Cautires_sp_Borneo
000081_Cautires_sp_Borneo
000090_Cautires_sp_Borneo
000246_Cautires_sp_Sumatra
000295_Cautires_sp_Sumatra
000314_Cautires_sp_Sumatra
000177_Cautires_sp_Malaysia
000040_Cautires_sp_Borneo
000037_Cautires_sp_Borneo
A00019_Cautires_sp_Taiwan
000411_Cautires_sp_Palawan
000339_Cautires_sp_Borneo
000342_Cautires_sp_Borneo
000294_Cautires_sp_Sumatra
000355_Cautires_sp_Jawa
000297_Cautires_sp_Sumatra
000262_Cautiressp_Borneo
000346_Cautires_sp_Borneo
000088_Cautires_sp_Malaysia
000206_Cautires_sp_Sumatra
000315_Cautires_sp_Sumatra
A00062_Cautires_sp_Philippines
000074_Cautires_sp_Borneo
000425_Cautires_sp_Palawan
000395_Cautires_sp_Palawan
A00578_Libnetis_China
000L12_Dihammatus_sp_A_Malaysia
A00546_Dihammatus_China
A00625_Dihammatus_Malaysia
001001_Dihammatus_sp_B_Sumatra_Barat
A00596_Dihammatus_Sumatra
A00600_Dihammatus_Laos
A00599_Dihammatus_China
001009_Dihammatus_sp_C_Sumatra_Barat
001017_Dihammatus_sp_D_Sumatra_Barat
A00466_Macrolycus_oreophilus_China
A00461_Macrolycus_bowringi_China
000828_Macrolycus_sp_B_Thailand
YL0206_Macrolycus_mucronatus_China
YL0403_Macrolycus_bicolor_China
YL0032_Macrolycus_flabellatus_Japan
ZL2005_Macrolycus_sp_A_Japan
A00465_Macrolycus_atronotatus_China
YL0185_Macrolycus_dotatus_China
000L18_Macrolycus_sp_A_China
YL0192_Macrolycus_ochraceus_China
A00462_Macrolycus_ligulatus_China
A00463_Macrolycus_sichuanensis_China
A00464_Macrolycus_galinae_China
A00594_Dihammatus_Malaysia
A00598_Dihammatus_Malaysia
VK0733_Atelius_China
YL0296_Lyponia_debilis_China
000815_Lyponia_delicatula_Japan
YL0319_Lyponia_kuatunensis_China
YL0314_Lyponia_muyuensis_China
000816_Lyponia_sp_China
A00468_Lyponia_tianquanensis_China
A00467_Lyponia_sp_China
YL0259_Ponyalis_ishigakiana_Japan
000817_Lyponia_quadricollis_Korea
YL0217_Ponyalis_alternata_China
YL0263_Ponyalis_oshimana_Japan
A00460_Ponyalis_laticornis_China
A00571_Lyponia_Laos
YL0282_Ponyalis_fukiensis_China
000L17_Lyponia_nigrohumeralis_China
YL0409_Ponyalis_gracilis_China
ZL2014_Lyponia_sp_Japan
ZL2016_Lyponia_sp_A_Japan
VM0021_Scarelus_nigricornis_Borneo
000L15_Scarelus_sp_C_Malaysia
VM0022_Scarelus_emasensis_Borneo
000583_Scarelus_sp_B_Malaysia
VM0005_Scarelus_similis_Borneo
VM0014_Scarelus_loksadoensis_Borneo
000582_Scarelus_sp_A_Malaysia
VM0019_Scarelus_baranciki_Borneo

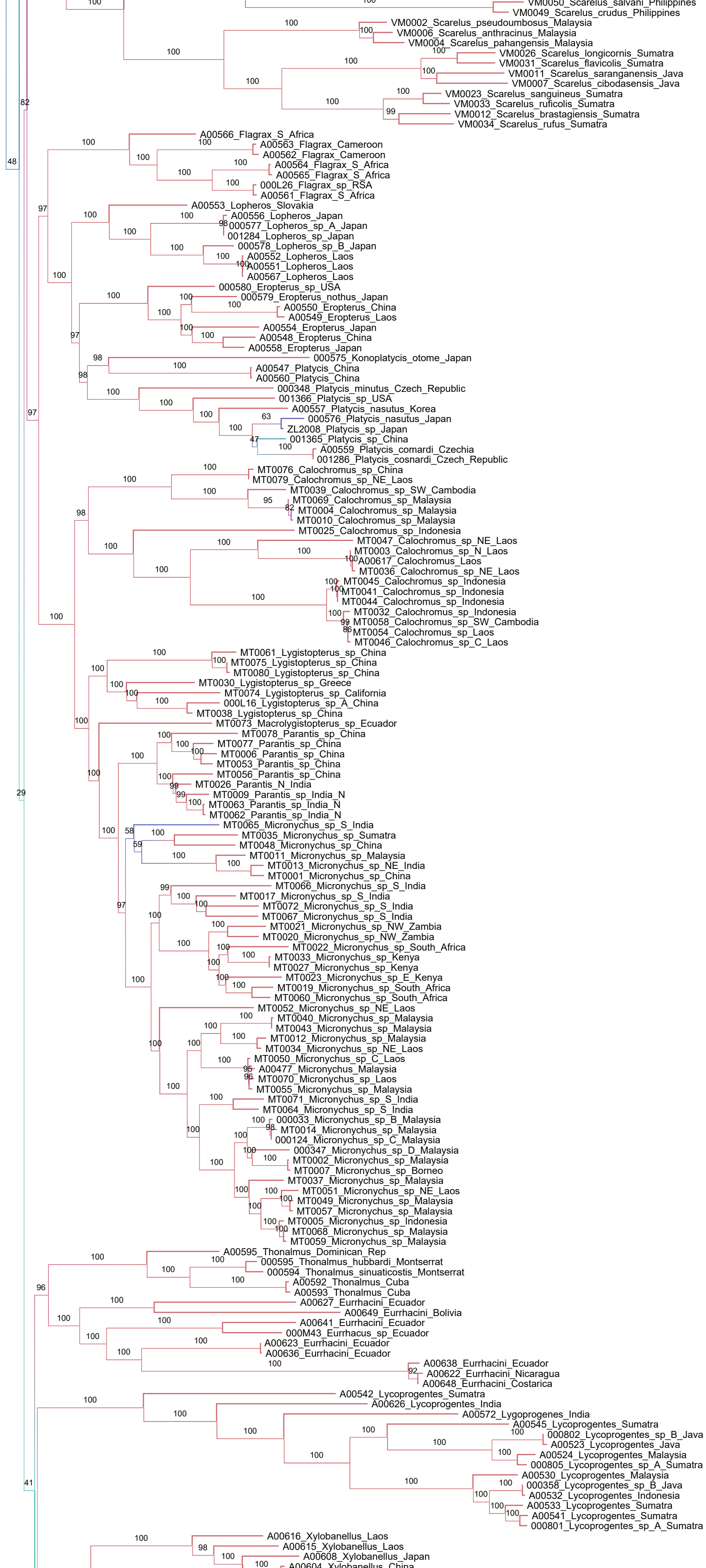
VM0050_Scarelus_salvani_Philippines
VM0049_Scarelus_crudus_Philippines
VM0002_Scarelus_pseudoumbosus_Malaysia
VM0006_Scarelus_anthracinus_Malaysia
VM0004_Scarelus_pahangensis_Malaysia
VM0026_Scarelus_longicornis_Sumatra
VM0031_Scarelus_flavicolis_Sumatra
VM0011_Scarelus_saranganensis_Java
VM0007_Scarelus_cibodasensis_Java
VM0023_Scarelus_sanguineus_Sumatra
VM0033_Scarelus_ruficolis_Sumatra
VM0012_Scarelus_brastagiensis_Sumatra
VM0034_Scarelus_rufus_Sumatra
A00566_Flagrax_S_Africa
A00563_Flagrax_Cameroon
A00562_Flagrax_Cameroon
A00564_Flagrax_S_Africa
A00565_Flagrax_S_Africa
000L26_Flagrax_sp_RSA
A00561_Flagrax_S_Africa
A00553_Lopheros_Slovakia
A00556_Lopheros_Japan
000577_Lopheros_sp_A_Japan
001284_Lopheros_sp_Japan
000578_Lopheros_sp_B_Japan
A00552_Lopheros_Laos
A00551_Lopheros_Laos
A00567_Lopheros_Laos
000580_Eropterus_sp_USA
000579_Eropterus_nothus_Japan
A00550_Eropterus_China
A00549_Eropterus_Laos
A00554_Eropterus_Japan
A00548_Eropterus_China
A00558_Eropterus_Japan
000575_Konoplatycis_otome_Japan
A00547_Platycis_China
A00560_Platycis_China
000348_Platycis_minutus_Czech_Republic
001366_Platycis_sp_USA
A00557_Platycis_nasutus_Korea
000576_Platycis_nasutus_Japan
ZL2008_Platycis_sp_Japan
001365_Platycis_sp_China
A00559_Platycis_comardi_Czechia
001286_Platycis_cosnardi_Czech_Republic
MT0076_Calochromus_sp_China
MT0079_Calochromus_sp_NE_Laos
MT0039_Calochromus_sp_SW_Cambodia
MT0069_Calochromus_sp_Malaysia
MT0004_Calochromus_sp_Malaysia
MT0010_Calochromus_sp_Malaysia
MT0025_Calochromus_sp_Indonesia
MT0047_Calochromus_sp_NE_Laos
MT0003_Calochromus_sp_N_Laos
A00617_Calochromus_Laos
MT0036_Calochromus_sp_NE_Laos
MT0045_Calochromus_sp_Indonesia
MT0041_Calochromus_sp_Indonesia
MT0044_Calochromus_sp_Indonesia
MT0032_Calochromus_sp_Indonesia
MT0058_Calochromus_sp_SW_Cambodia
MT0054_Calochromus_sp_Laos
MT0046_Calochromus_sp_C_Laos
MT0061_Lygistopterus_sp_China
MT0075_Lygistopterus_sp_China
MT0080_Lygistopterus_sp_China
MT0030_Lygistopterus_sp_Greece
MT0074_Lygistopterus_sp_California
000L16_Lygistopterus_sp_A_China
MT0038_Lygistopterus_sp_China
MT0073_Macrolygistopterus_sp_Ecuador
MT0078_Parantis_sp_China
MT0077_Parantis_sp_China
MT0006_Parantis_sp_China
MT0053_Parantis_sp_China
MT0056_Parantis_sp_China
MT0026_Parantis_N_India
MT0009_Parantis_sp_India_N
MT0063_Parantis_sp_India_N
MT0062_Parantis_sp_India_N
MT0065_Micronychus_sp_S_India
MT0035_Micronychus_sp_Sumatra
MT0048_Micronychus_sp_China
MT0011_Micronychus_sp_Malaysia
MT0013_Micronychus_sp_NE_India
MT0001_Micronychus_sp_China
MT0066_Micronychus_sp_S_India
MT0017_Micronychus_sp_S_India
MT0072_Micronychus_sp_S_India
MT0067_Micronychus_sp_S_India
MT0021_Micronychus_sp_NW_Zambia
MT0020_Micronychus_sp_NW_Zambia
MT0022_Micronychus_sp_South_Africa
MT0033_Micronychus_sp_Kenya
MT0027_Micronychus_sp_Kenya
MT0023_Micronychus_sp_E_Kenya
MT0019_Micronychus_sp_South_Africa
MT0060_Micronychus_sp_South_Africa
MT0052_Micronychus_sp_NE_Laos
MT0040_Micronychus_sp_Malaysia
MT0043_Micronychus_sp_Malaysia
MT0012_Micronychus_sp_Malaysia
MT0034_Micronychus_sp_NE_Laos
MT0050_Micronychus_sp_C_Laos
A00477_Micronychus_Malaysia
MT0070_Micronychus_sp_Laos
MT0055_Micronychus_sp_Malaysia
MT0071_Micronychus_sp_S_India
MT0064_Micronychus_sp_S_India
000033_Micronychus_sp_B_Malaysia
MT0014_Micronychus_sp_Malaysia
000124_Micronychus_sp_C_Malaysia
000347_Micronychus_sp_D_Malaysia
MT0002_Micronychus_sp_Malaysia
MT0007_Micronychus_sp_Borneo
MT0037_Micronychus_sp_Malaysia
MT0051_Micronychus_sp_NE_Laos
MT0049_Micronychus_sp_Malaysia
MT0057_Micronychus_sp_Malaysia
MT0005_Micronychus_sp_Indonesia
MT0068_Micronychus_sp_Malaysia
MT0059_Micronychus_sp_Malaysia
A00595_Thonalmus_Dominican_Rep
000595_Thonalmus_hubbardi_Montserrat
000594_Thonalmus_sinuaticostis_Montserrat
A00592_Thonalmus_Cuba
A00593_Thonalmus_Cuba
A00627_Eurrhacini_Ecuador
A00649_Eurrhacini_Bolivia
A00641_Eurrhacini_Ecuador
000M43_Eurrhacus_sp_Ecuador
A00623_Eurrhacini_Ecuador
A00636_Eurrhacini_Ecuador
A00638_Eurrhacini_Ecuador
A00622_Eurrhacini_Nicaragua
A00648_Eurrhacini_Costarica
A00542_Lycoprogentes_Sumatra
A00626_Lycoprogentes_India
A00572_Lygoprogenes_India
A00545_Lycoprogentes_Sumatra
000802_Lycoprogentes_sp_B_Java
A00523_Lycoprogentes_Java
A00524_Lycoprogentes_Malaysia
000805_Lycoprogentes_sp_A_Sumatra
A00530_Lycoprogentes_Malaysia
000358_Lycoprogentes_sp_B_Java
A00532_Lycoprogentes_Indonesia
A00533_Lycoprogentes_Sumatra
A00541_Lycoprogentes_Sumatra
000801_Lycoprogentes_sp_A_Sumatra
A00616_Xylobanellus_Laos
A00615_Xylobanellus_Laos
A00608_Xylobanellus_Japan
A00604_Xylobanellus_China

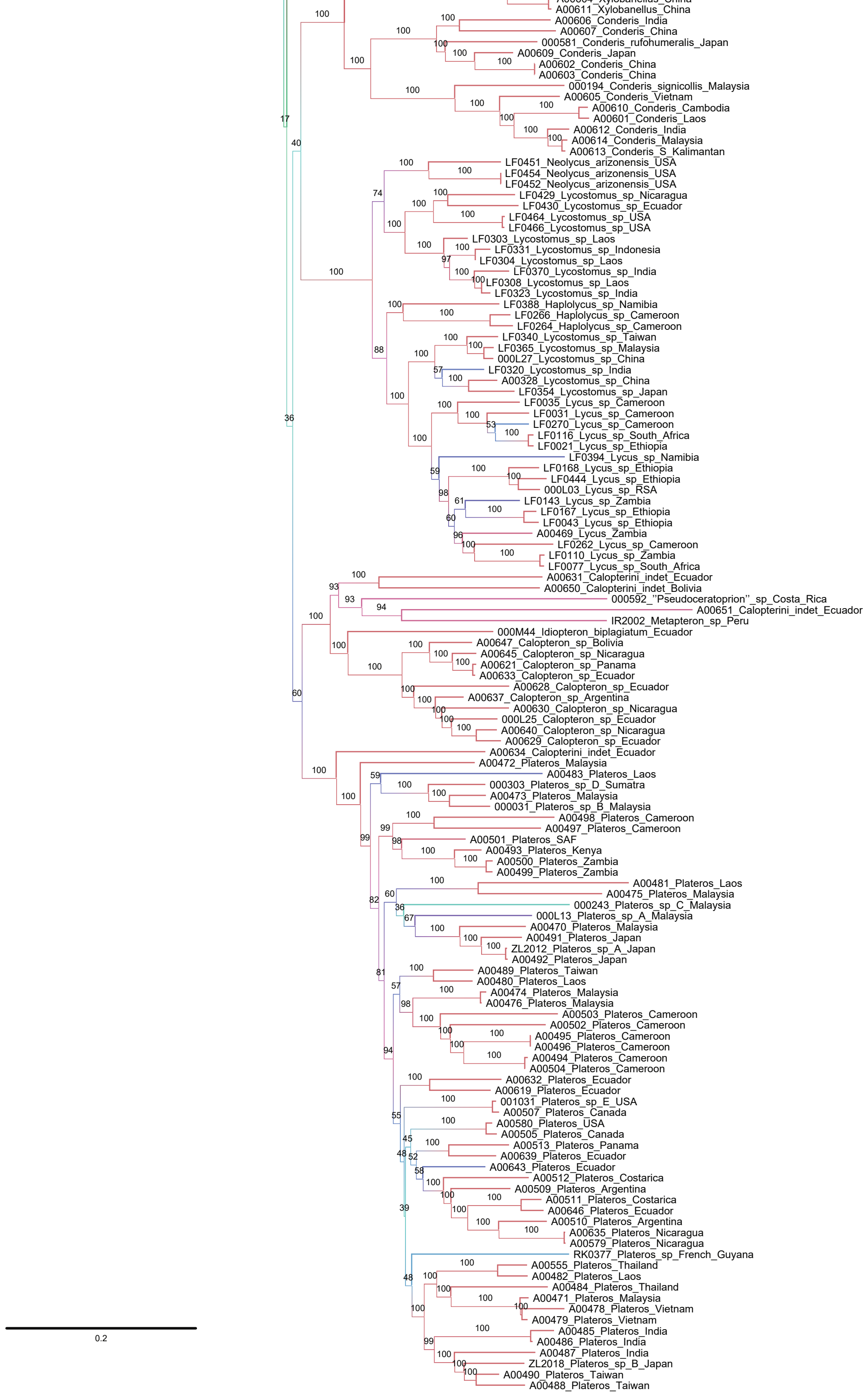

Figure S3. The iQ-tree topology inferred from the MAFFT alignment of all markers and Iberobaenia as a single outgroup.

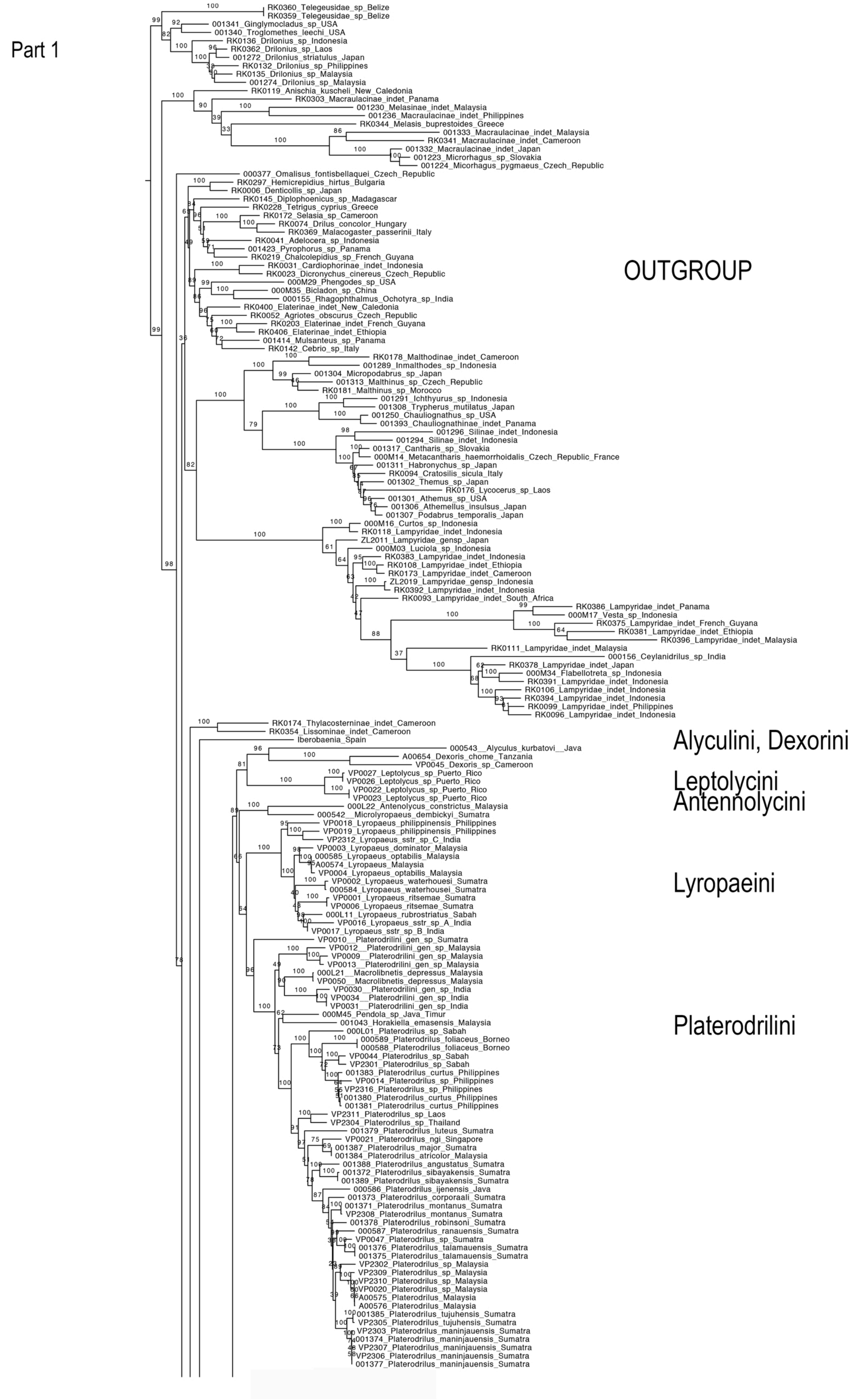

Figure S4. The iQ-tree topology inferred from the MAFFT alignment of all markers and 89 taxa as an outgroup with the -bnni parameter applied. All taxa represented, full resolution tree of net-winged beetles.

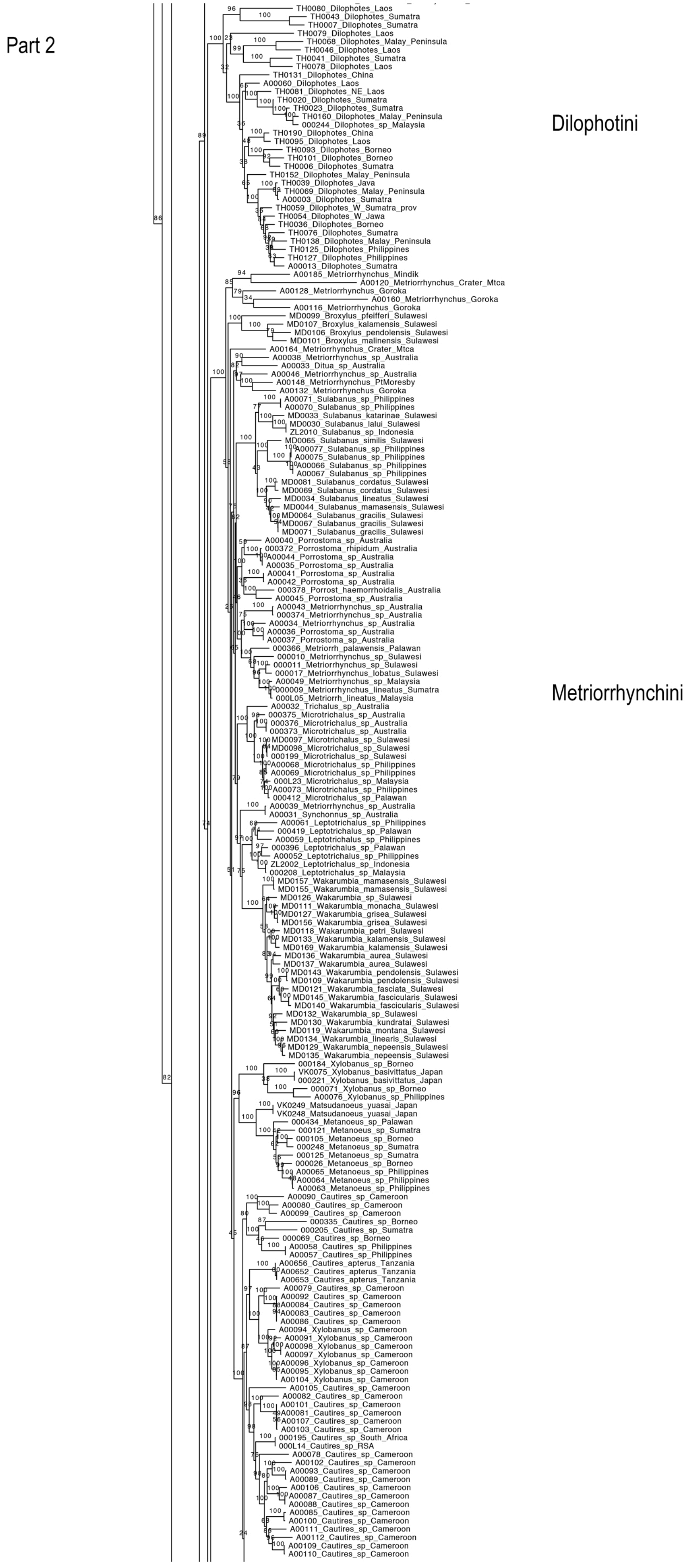

Figure S4. The iQ-tree topology inferred from the MAFFT alignment of all markers and 89 taxa as an outgroup with the -bnni parameter applied. All taxa represented, full resolution tree of net-winged beetles.

Part 3

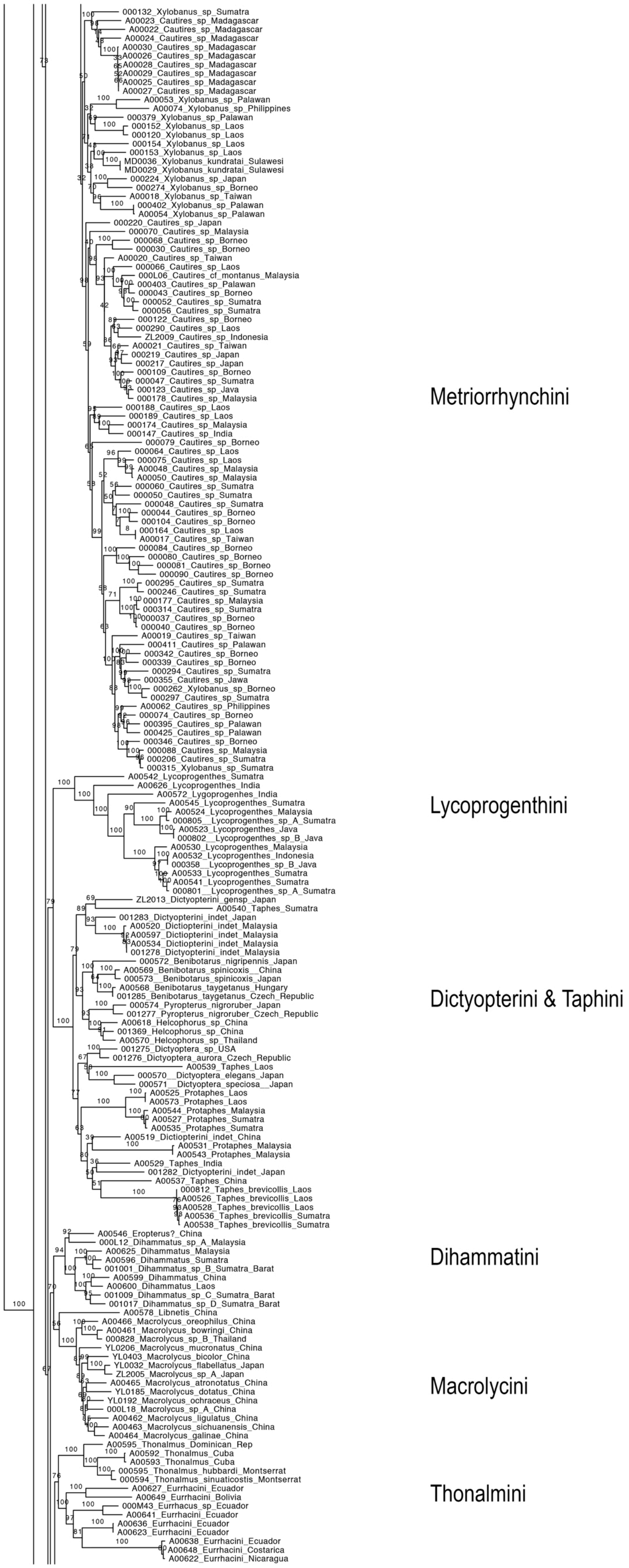

Figure S4. The iQ-tree topology inferred from the MAFFT alignment of all markers and 89 taxa as an outgroup with the -bnni parameter applied. All taxa represented, full resolution tree of net-winged beetles.

Part 4

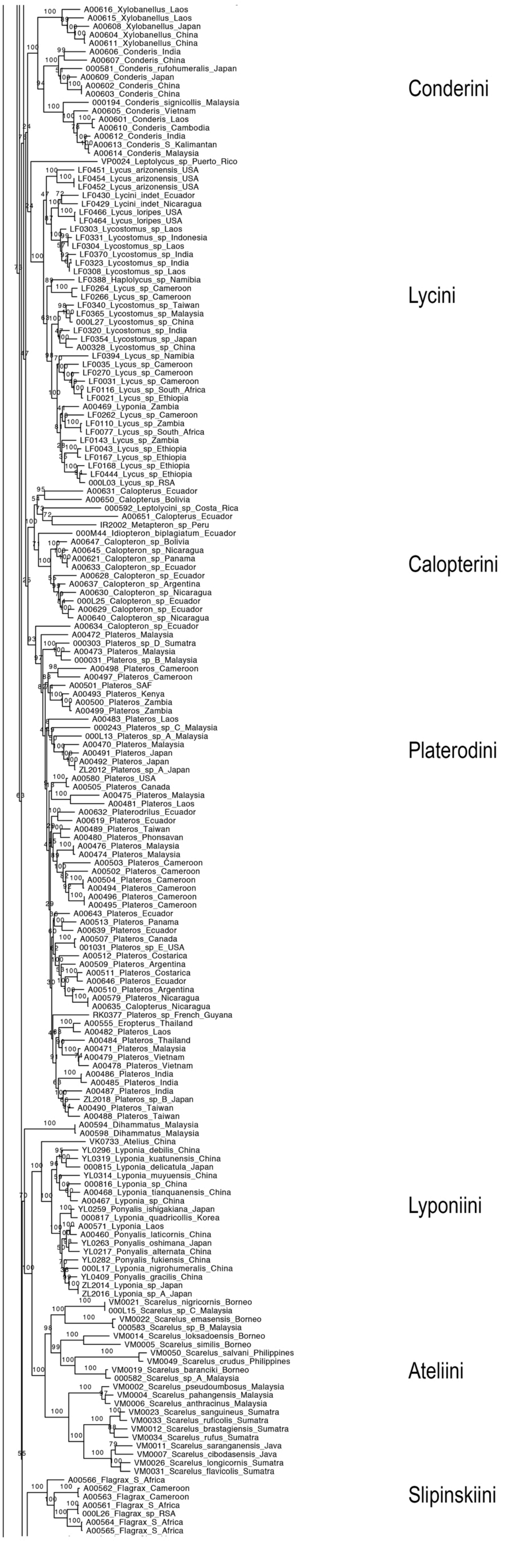

Figure S4. The iQ-tree topology inferred from the MAFFT alignment of all markers and 89 taxa as an outgroup with the -bnni parameter applied. All taxa represented, full resolution tree of net-winged beetles.

Part 5

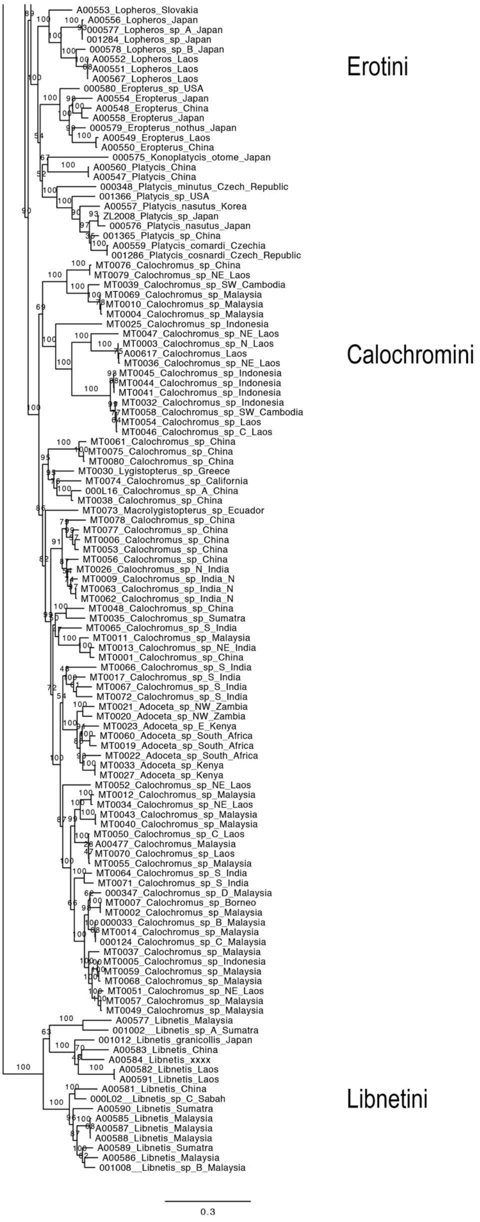

Figure S4. The iQ-tree topology inferred from the MAFFT alignment of all markers and 89 taxa as an outgroup with the -bnni parameter applied. All taxa represented, full resolution tree of net-winged beetles.

Part 1

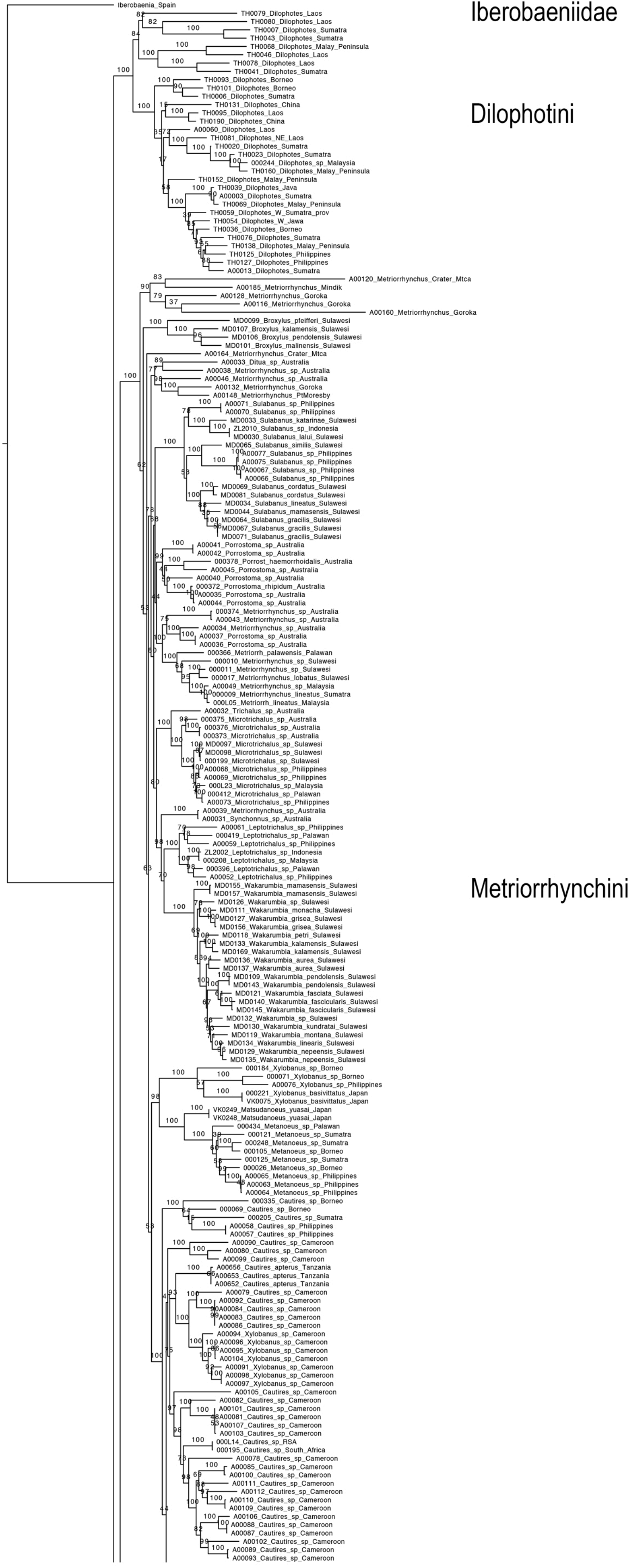

Figure S5. The iQ-tree topology inferred from the MAFFT alignment of all markers and *Iberobaenia* as a single outgroup with the -bnni parameter applied.

Part 2

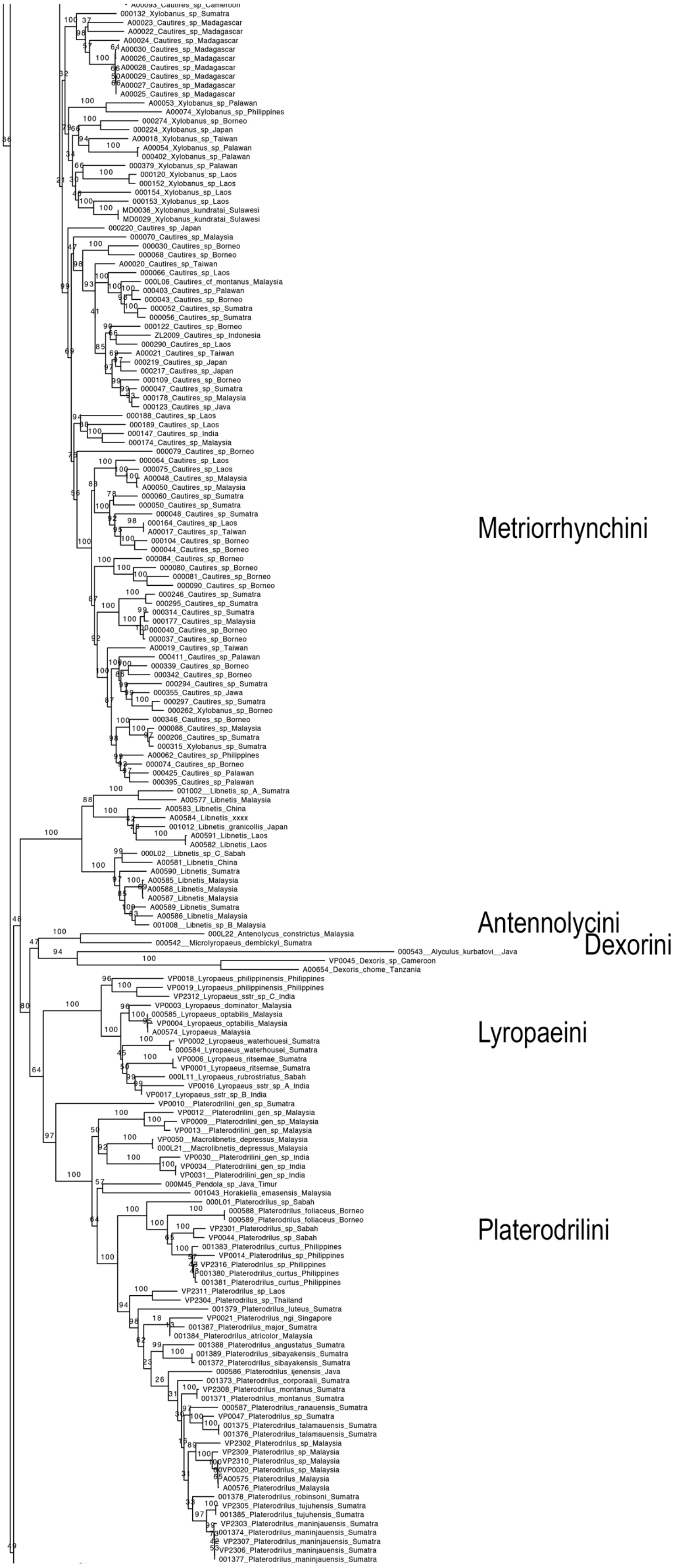

Figure S5. The iQ-tree topology inferred from the MAFFT alignment of all markers and *Iberobaenia* as a single outgroup with the -bnni parameter applied.

Part 3

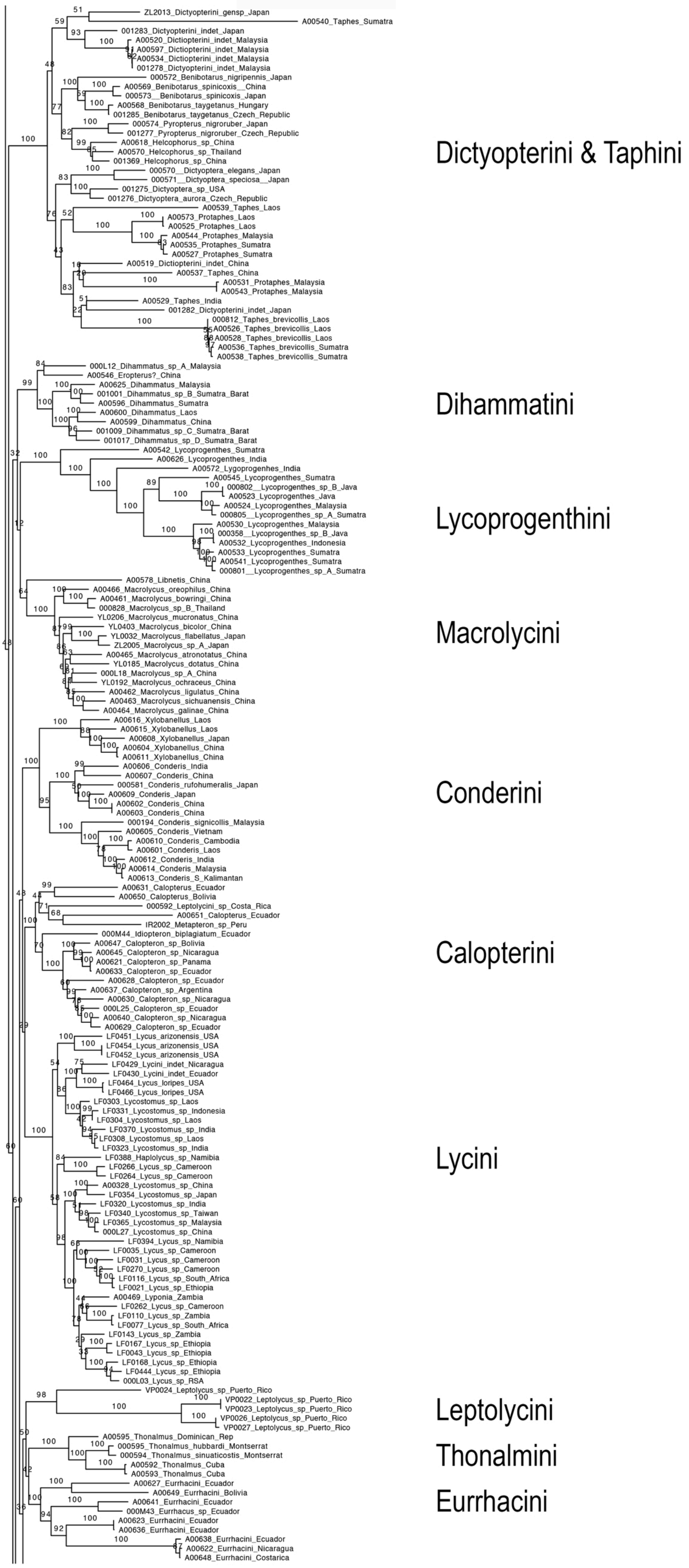

Figure S5. The iQ-tree topology inferred from the MAFFT alignment of all markers and *Iberobaenia* as a single outgroup with the -bnni parameter applied.

Part 4

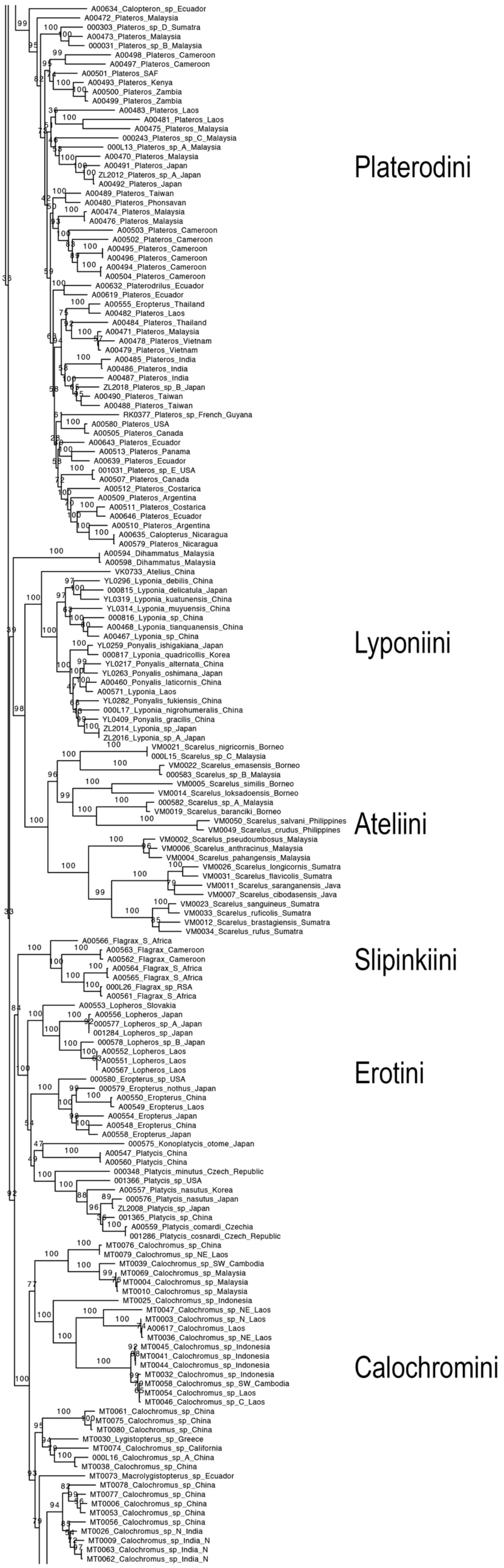

Figure S5. The iQ-tree topology inferred from the MAFFT alignment of all markers and Iberobaenia as a single outgroup with the -bnni parameter applied.

Part 5

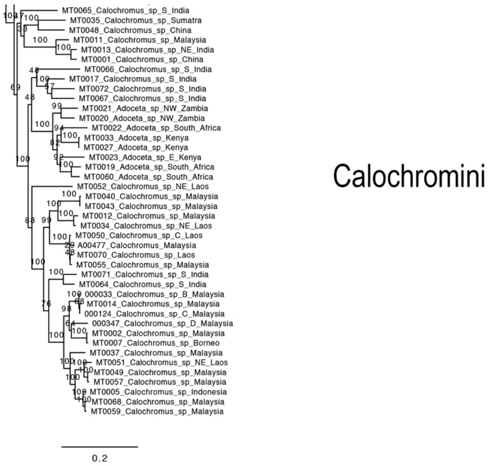

Figure S5. The iQ-tree topology inferred from the MAFFT alignment of all markers and *Iberobaenia* as a single outgroup with the -bnni parameter applied.

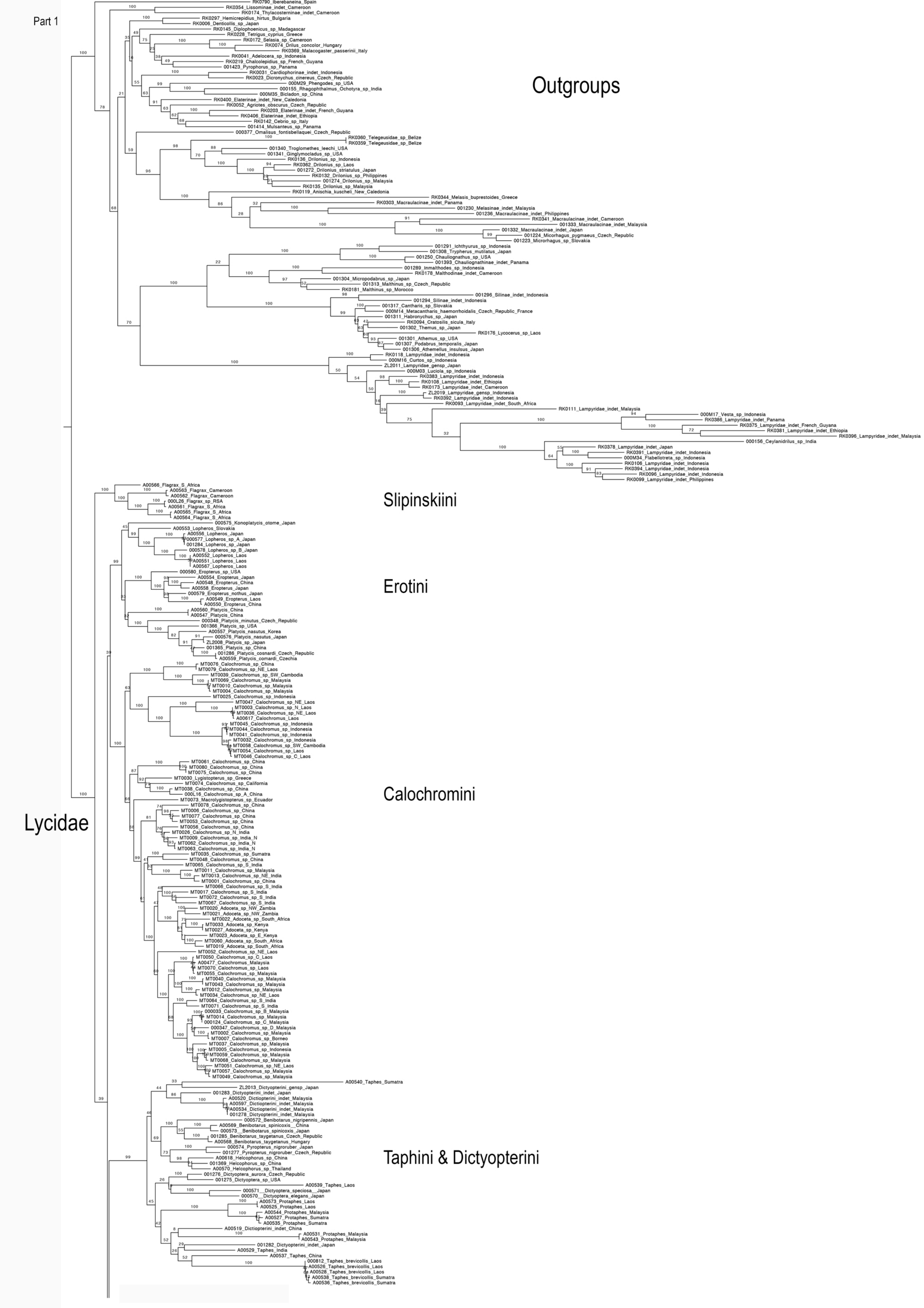

Figure S6. The RAXML topology inferred from the MAFFT alignment of all markers and 89 taxa as an outgroup.
All taxa represented, full resolution tree of net-winged beetles.

Part 2

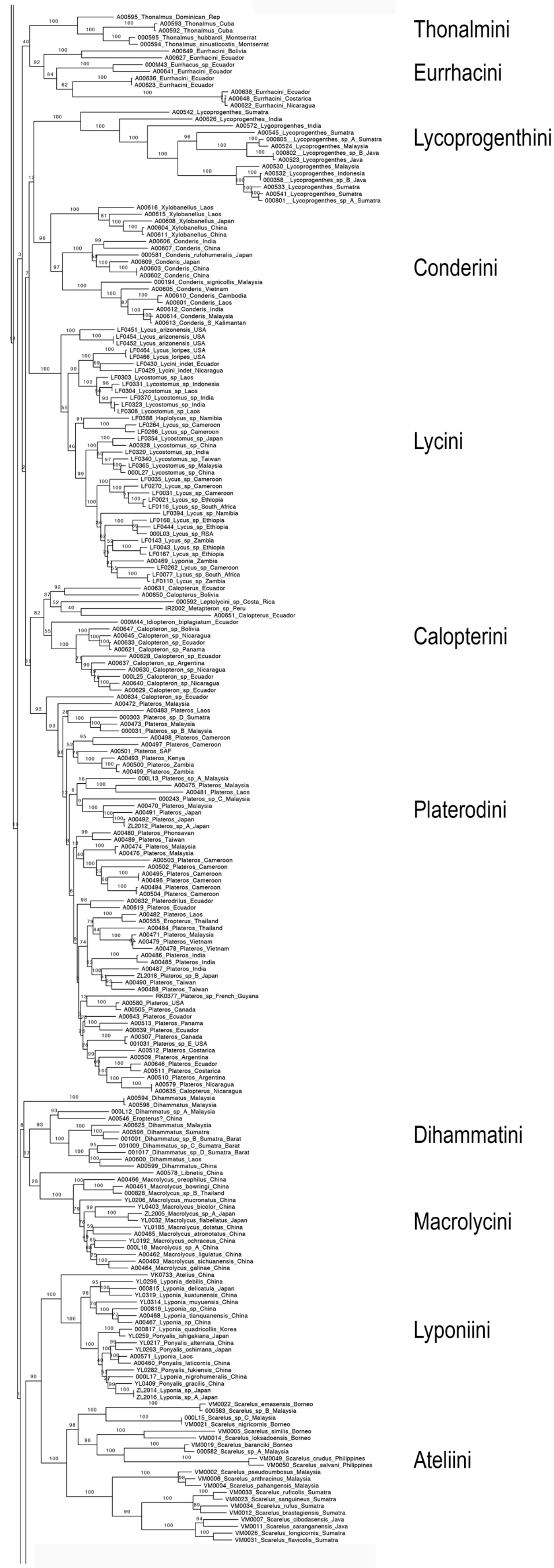

Figure S6. The RAXML topology inferred from the MAFFT alignment of all markers and 89 taxa as an outgroup. All taxa represented, full resolution tree of net-winged beetles.

Part 3

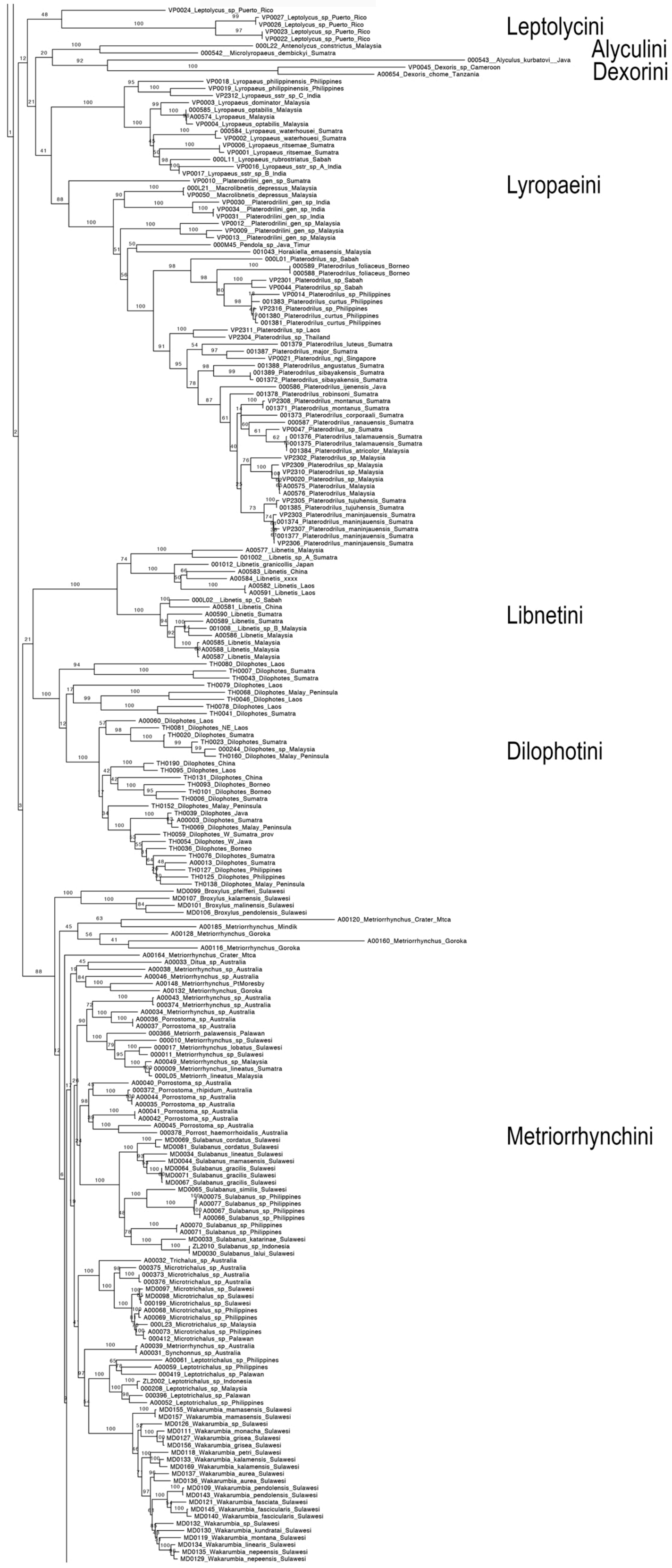

Figure S6. The RAXML topology inferred from the MAFFT alignment of all markers and 89 taxa as an outgroup. All taxa represented, full resolution tree of net-winged beetles.

Part 4

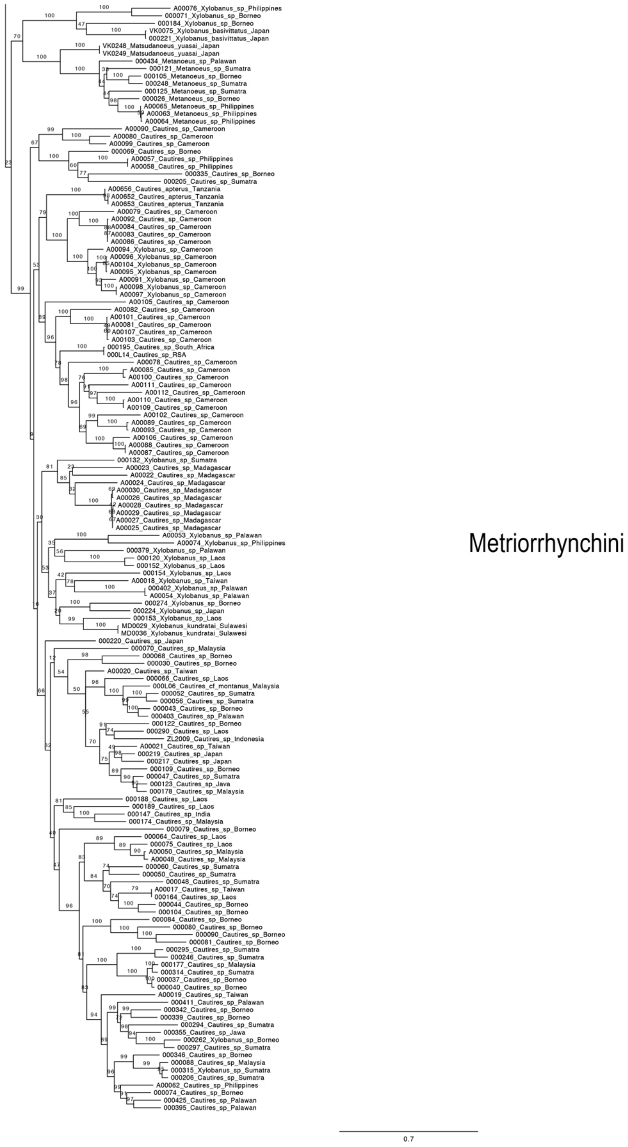

Figure S6. The RAXML topology inferred from the MAFFT alignment of all markers and 89 taxa as an outgroup.
All taxa represented, full resolution tree of net-winged beetles.

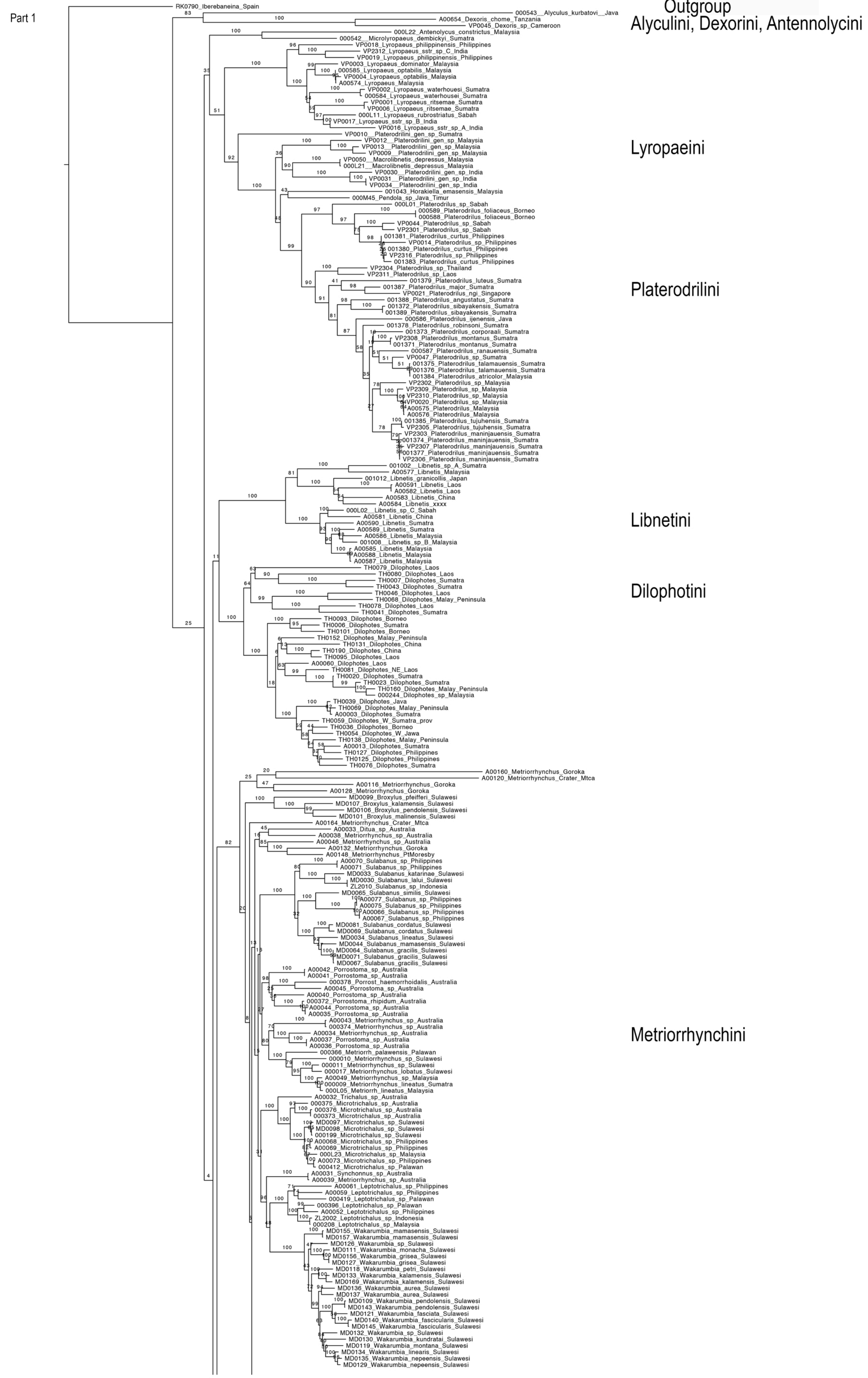

Figure S7. The RAxML topology inferred from the MAFFT alignment of all markers and Iberobaenia as a single outgroup.

Part 2

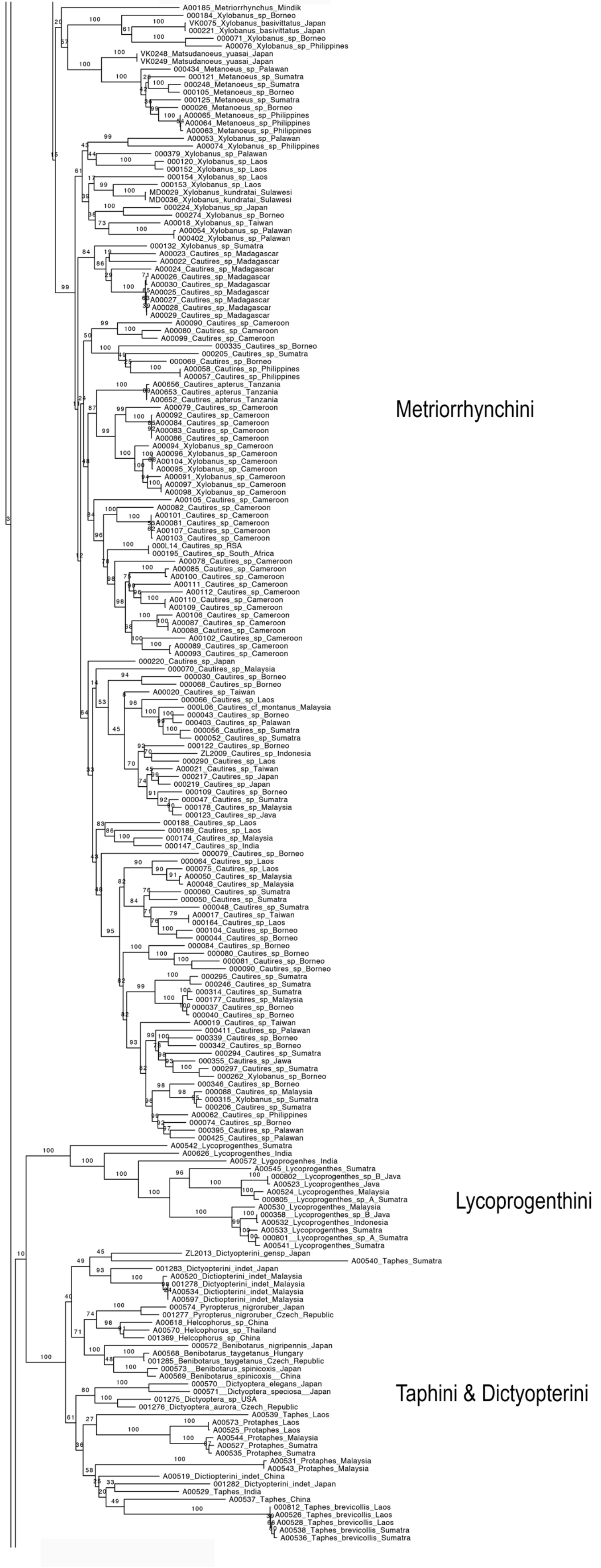

Figure S7. The RAxML topology inferred from the MAFFT alignment of all markers and Iberobaenia as a single outgroup.

Part 3

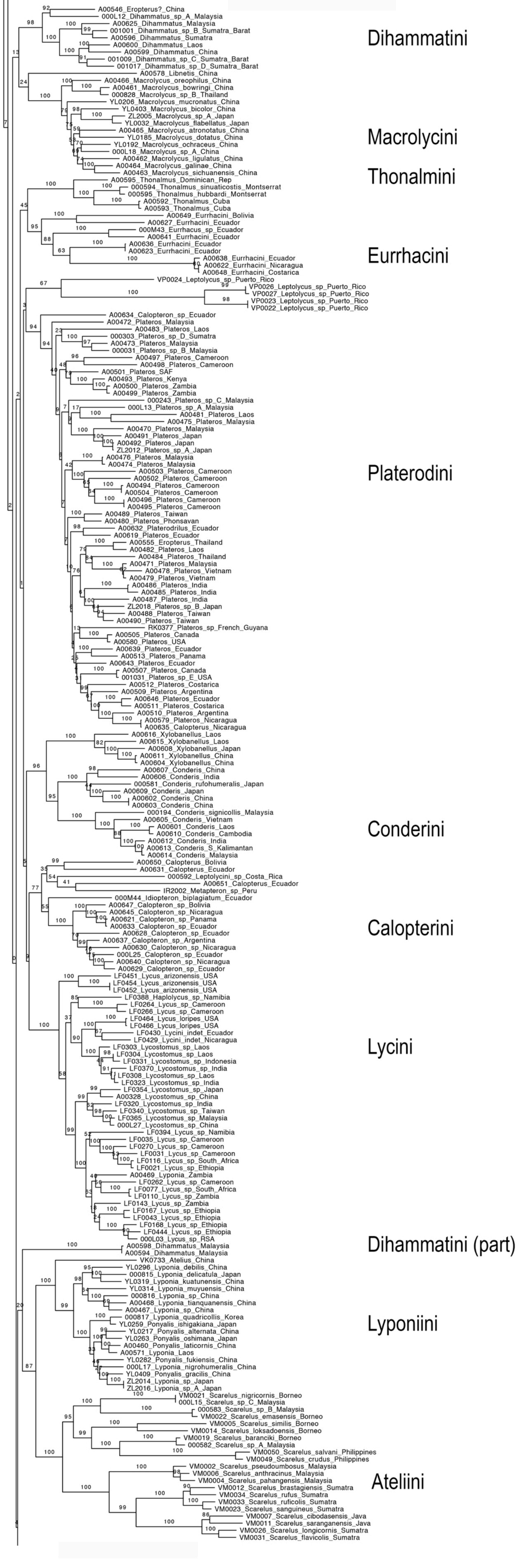

Figure S7. The RAxML topology inferred from the MAFFT alignment of all markers and Iberobaenia as a single outgroup.

Part 4

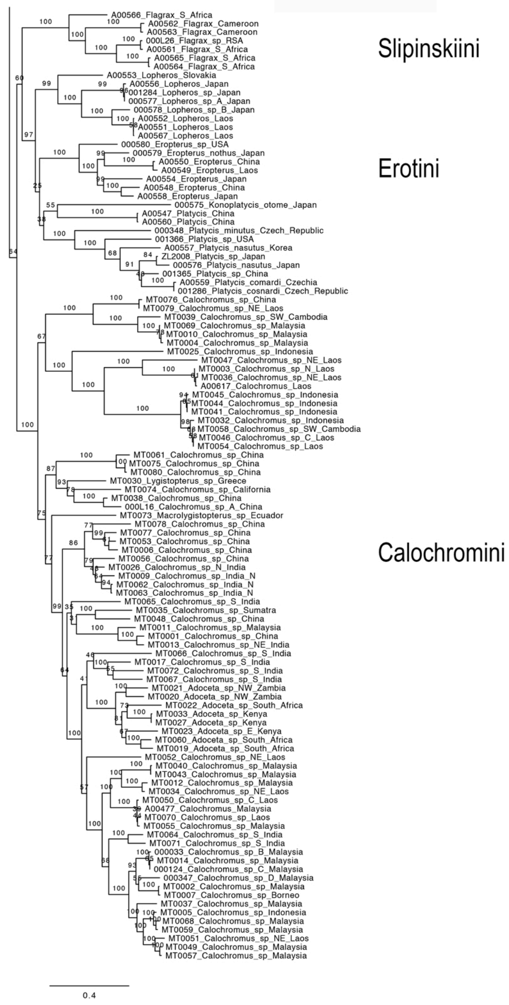

Figure S7. The RAxML topology inferred from the MAFFT alignment of all markers and Iberobaenia as a single outgroup.

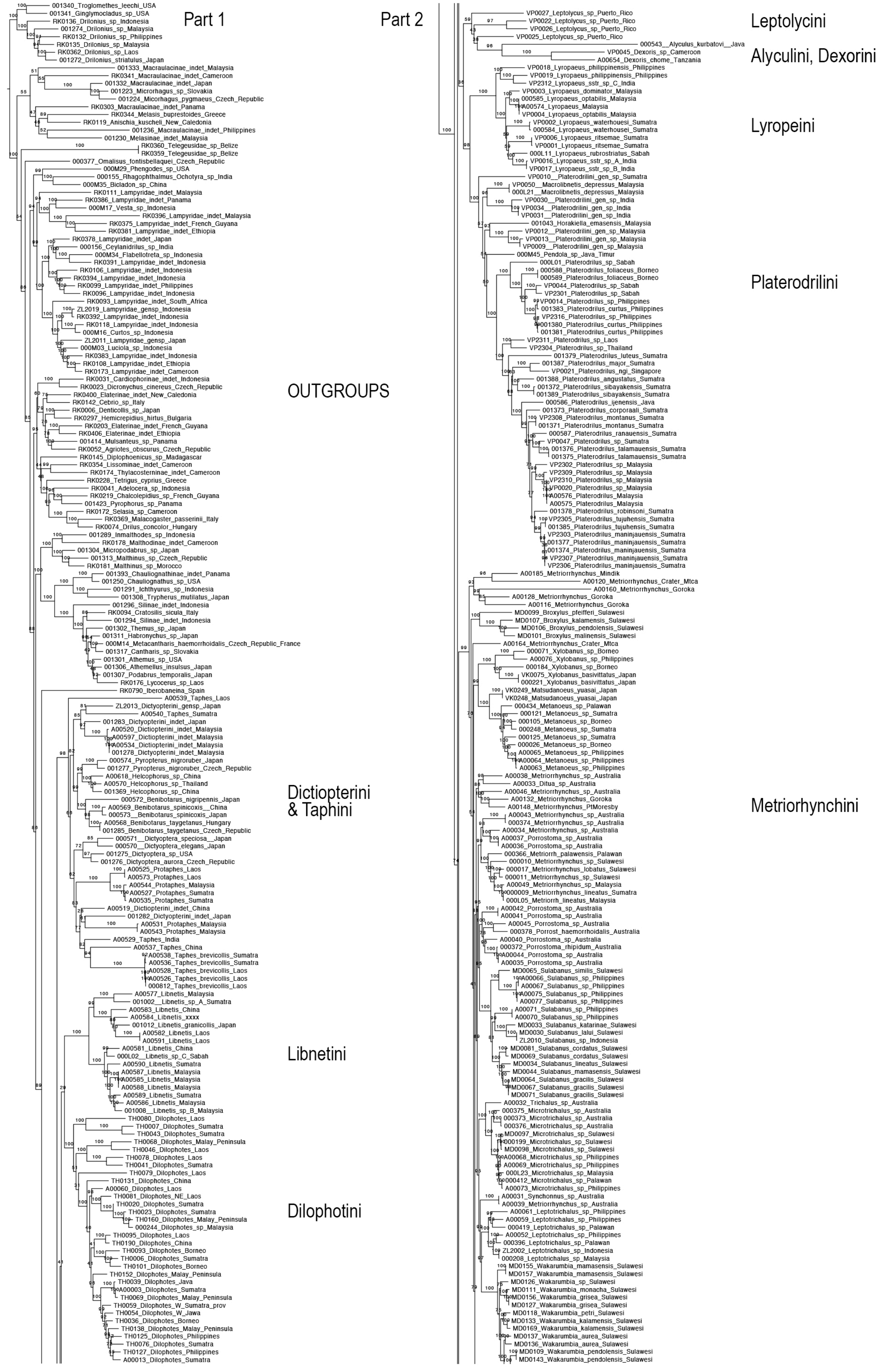

Figure S8. The IQ-tree topology inferred from the MAFFT alignment of rRNA markers and 89 taxa as an outgroup with the -bnni option applied.

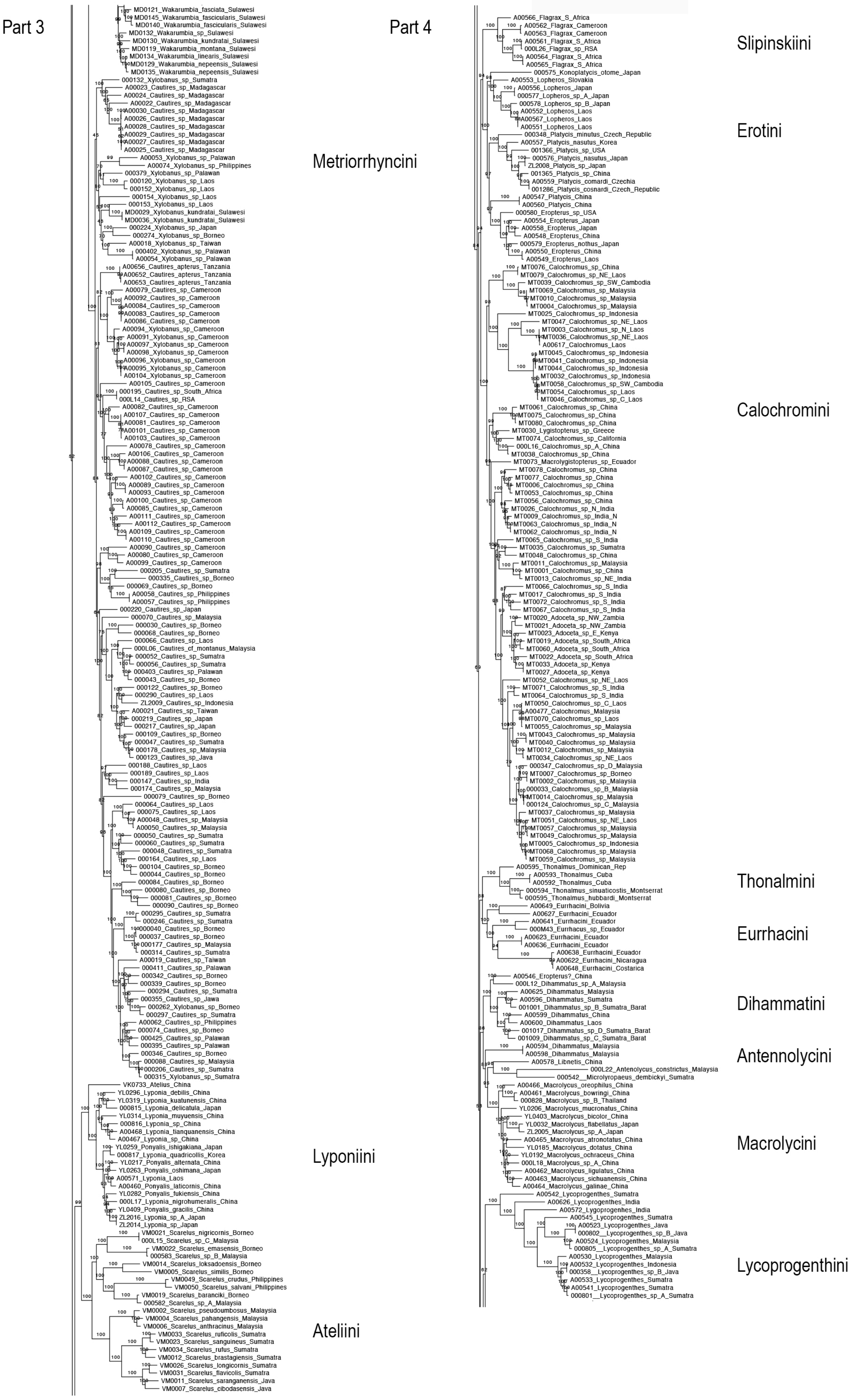

Figure S8. The IQ-tree topology inferred from the MAFFT alignment of rRNA markers and 89 taxa as an outgroup with the -bnni option applied.

Part 5

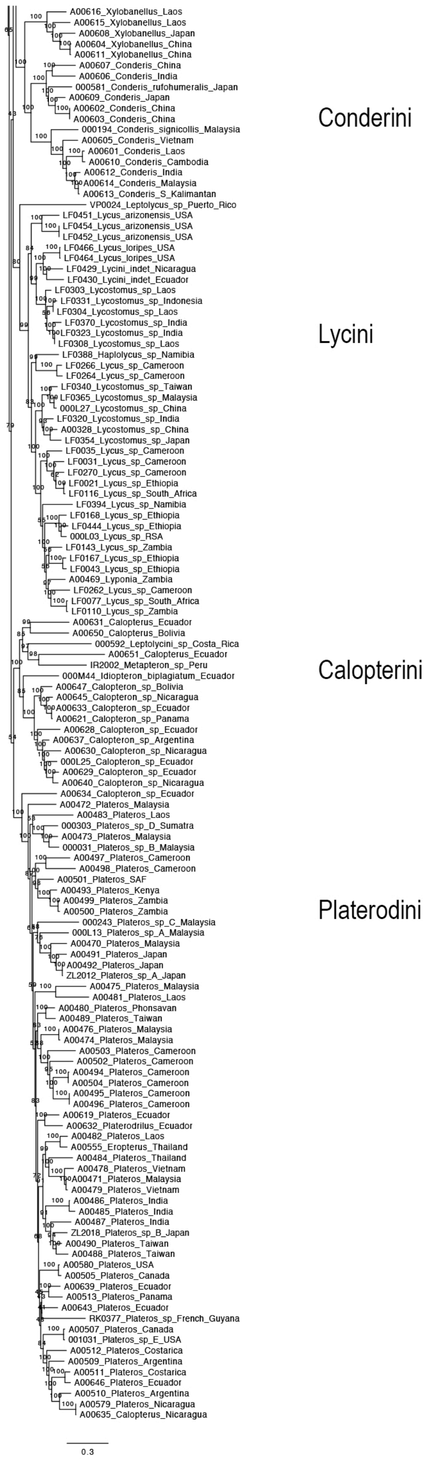

Figure S8. The IQ-tree topology inferred from the MAFFT alignment of rRNA markers and 89 taxa as an outgroup with the -bnni option applied.

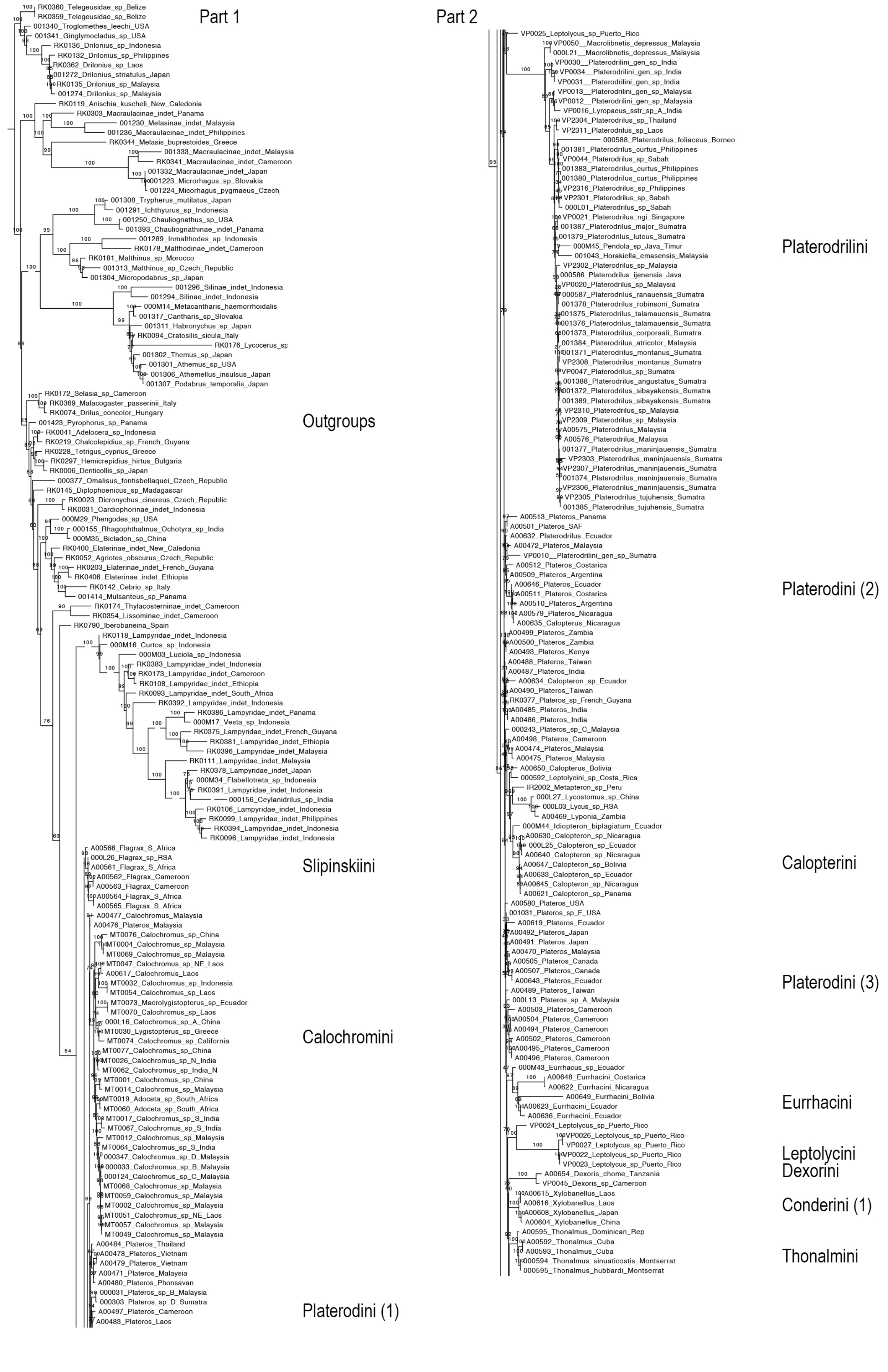

Figure S9. The IQ-tree topology inferred from the MAFFT alignment of mtDNA markers and 89 taxa as an outgroup with the -bnni option applied.

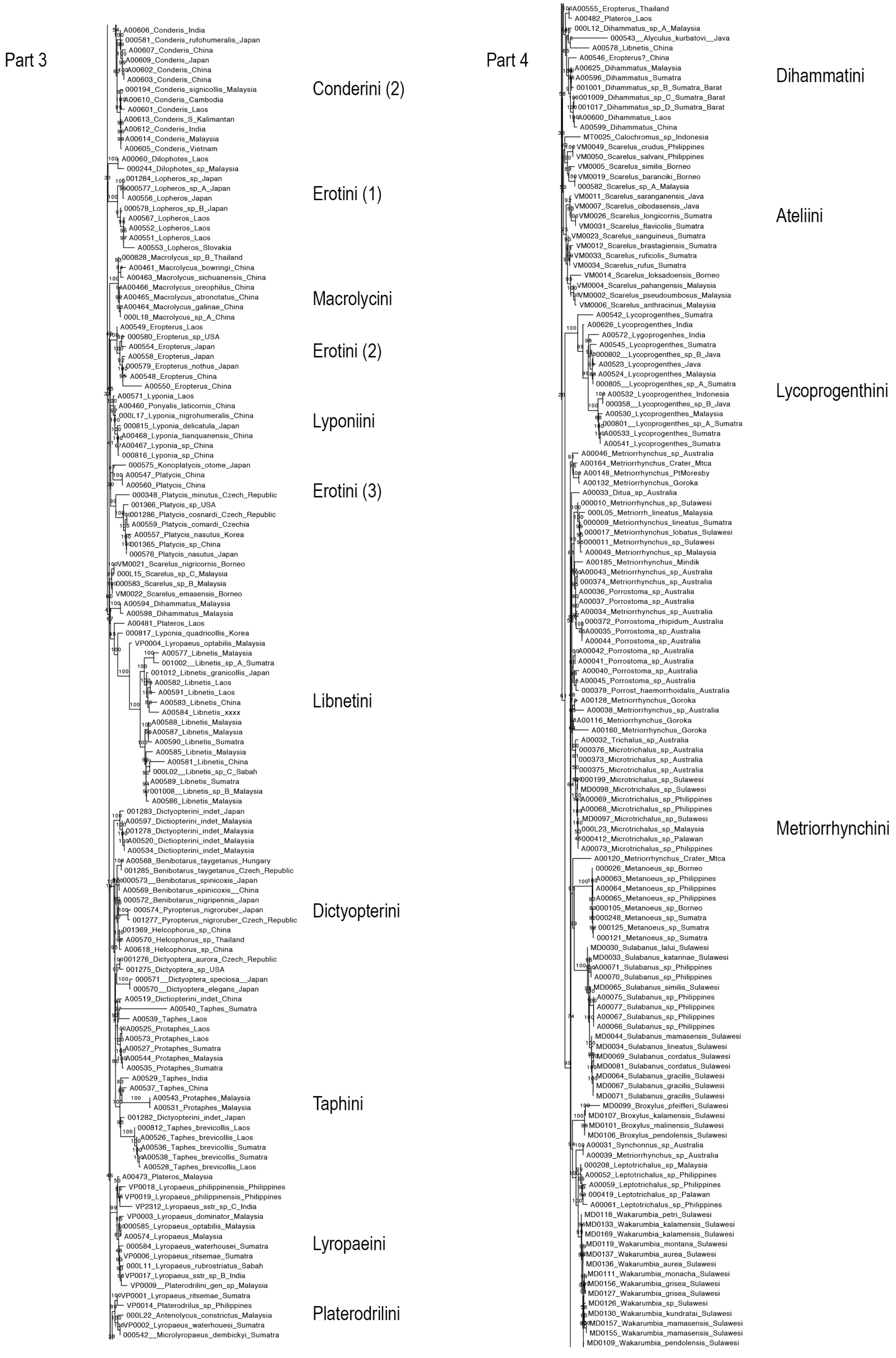

Figure S9. The IQ-tree topology inferred from the MAFFT alignment of mtDNA markers and 89 taxa as an outgroup with the -bnni option applied.

Part 5

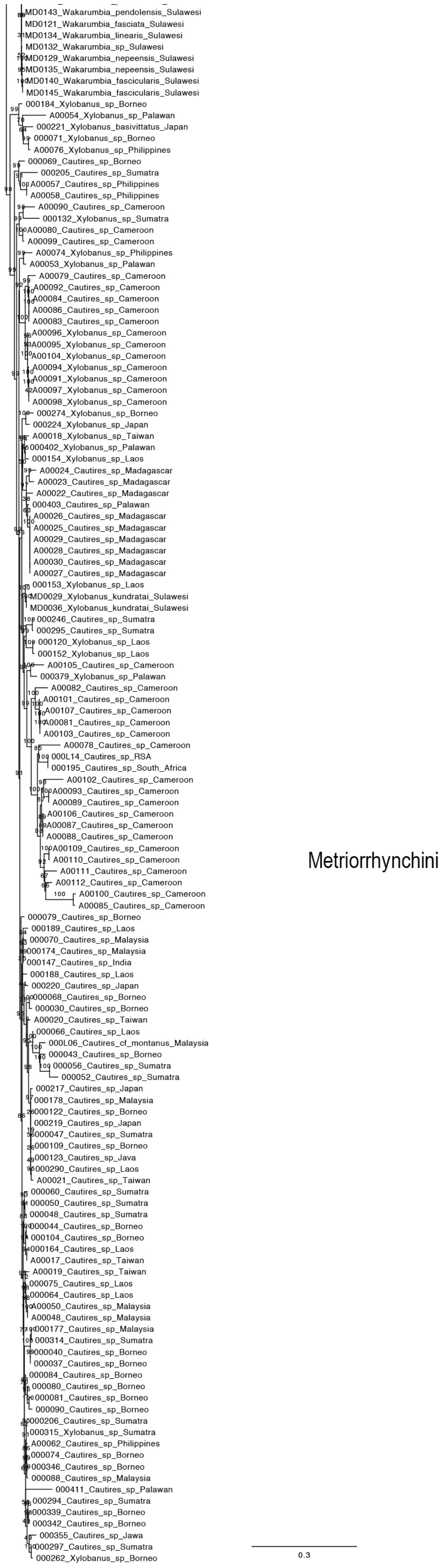

Figure S9. The IQ-tree topology inferred from the MAFFT alignment of mtDNA markers and 89 taxa as an outgroup with the -bnni option applied.

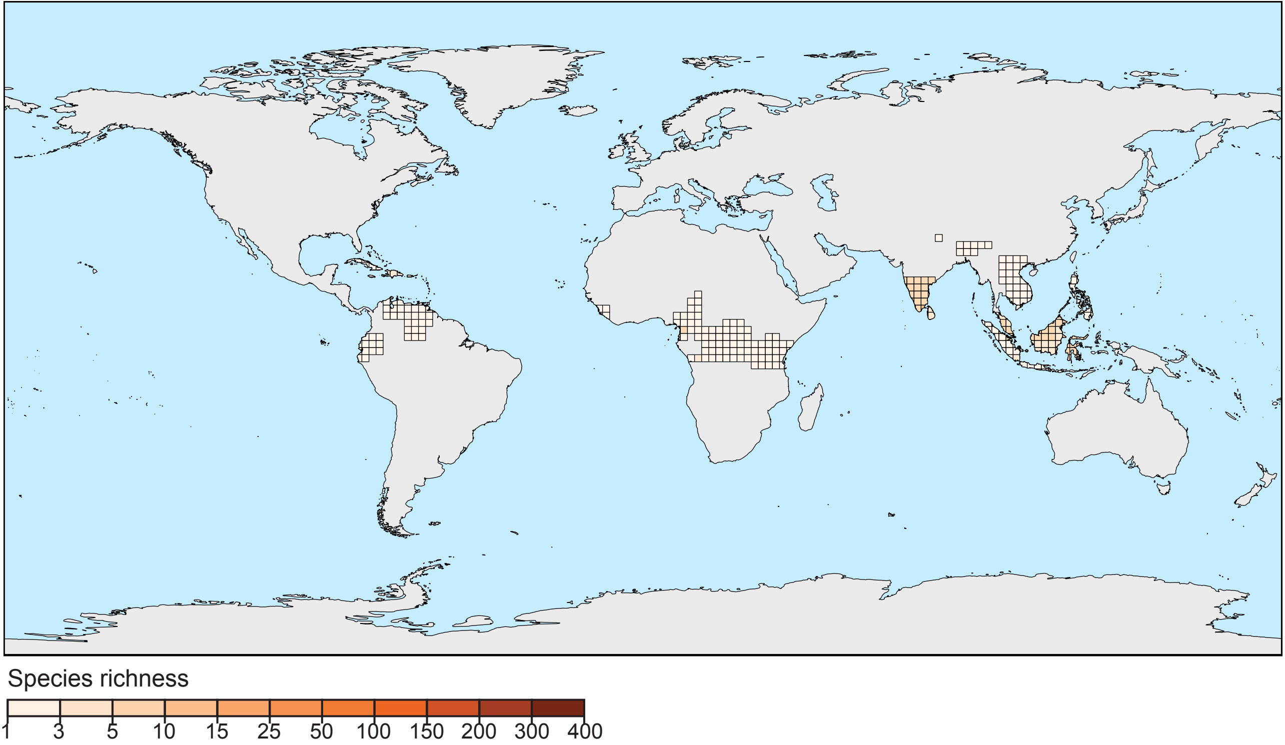

Fig. S10. Distribution and species diversity of the tribes Leptolycini, Dexorini and Lyropaeini

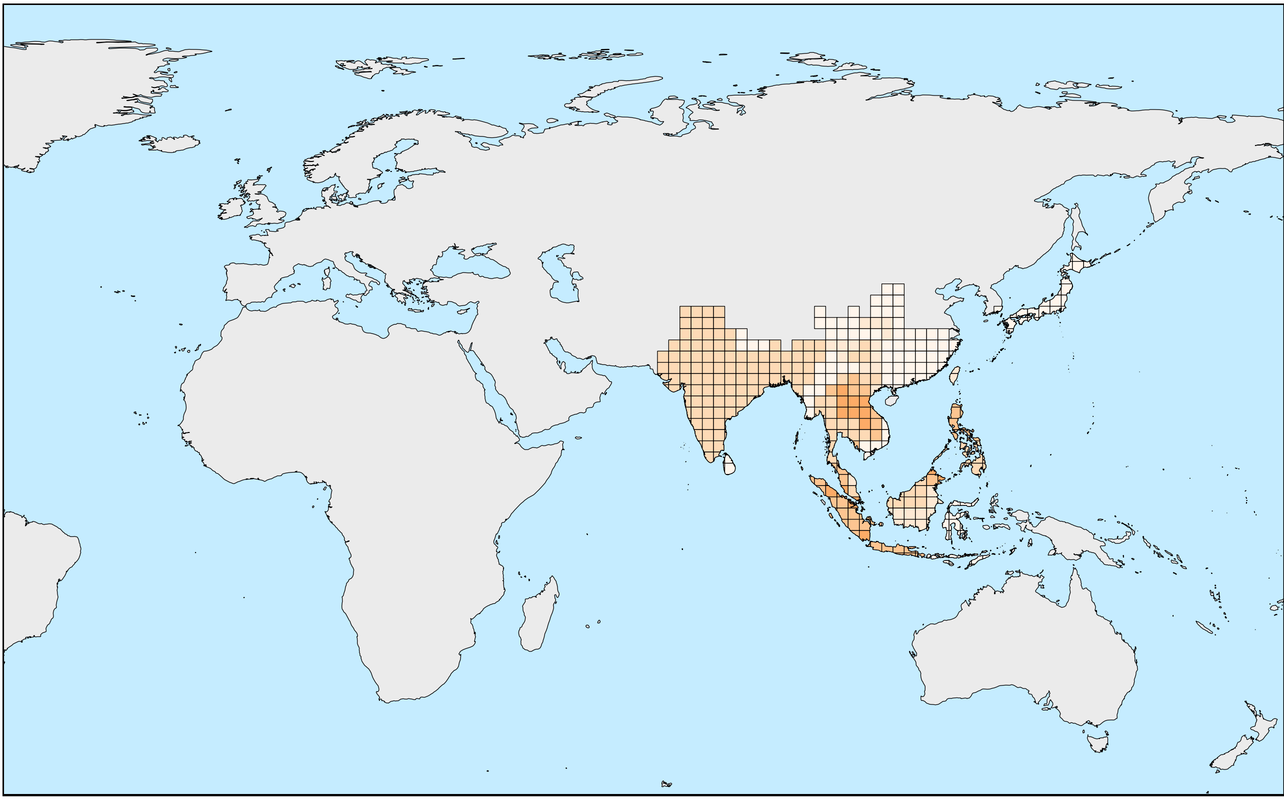

Fig. S11. Distribution and species diversity of the subfamily Libnetinae

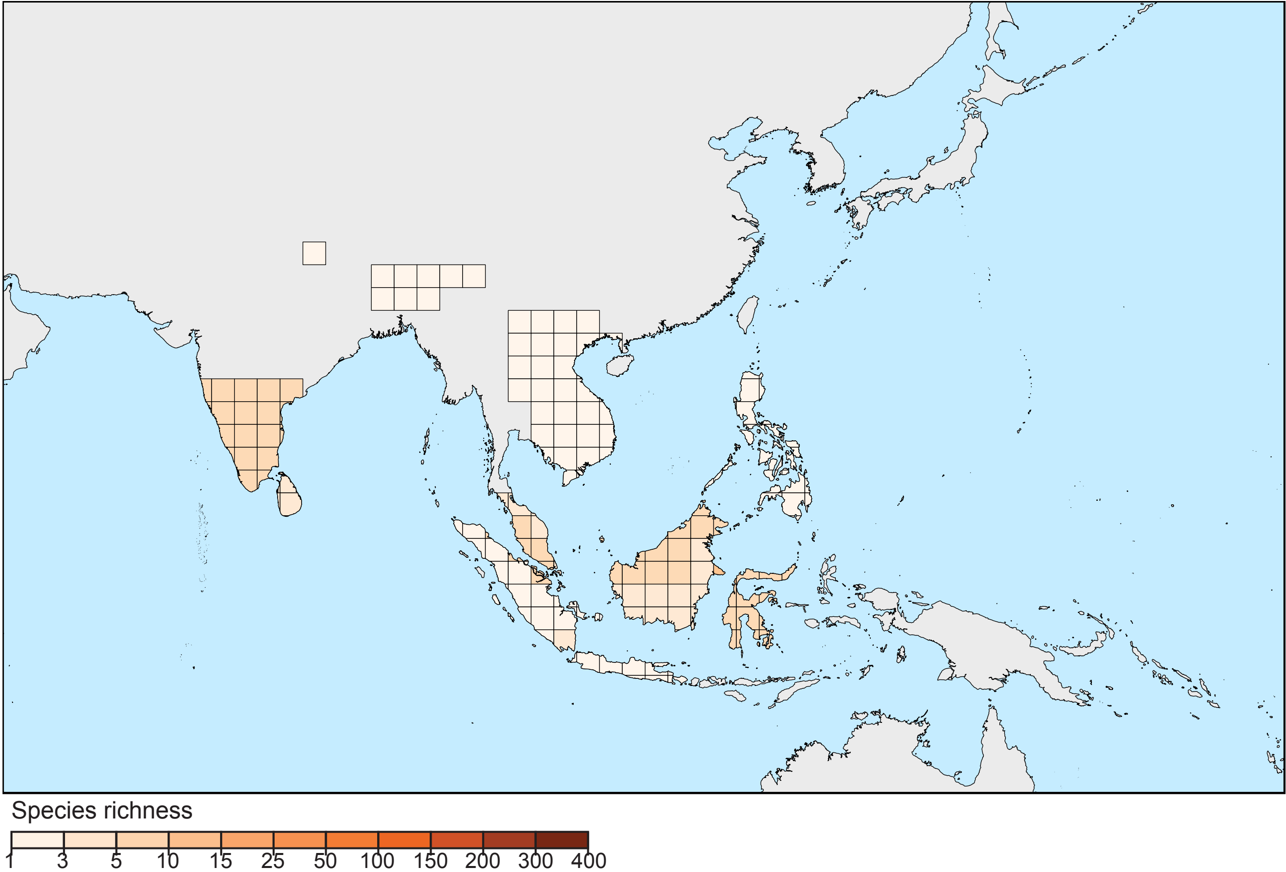

Fig. S12. Distribution and species diversity of the tribe Lyropaeini

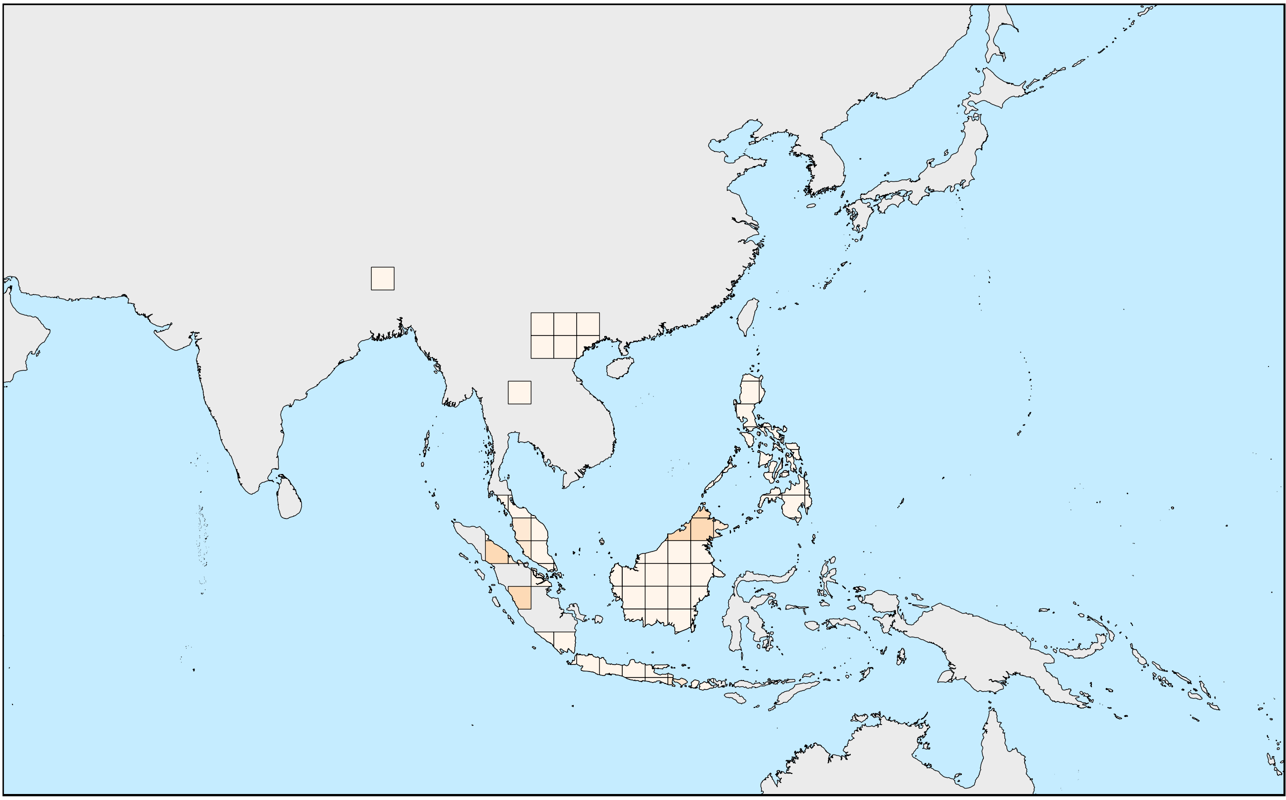

Fig. S13. Distribution and species diversity of the tribe Platerodrilini

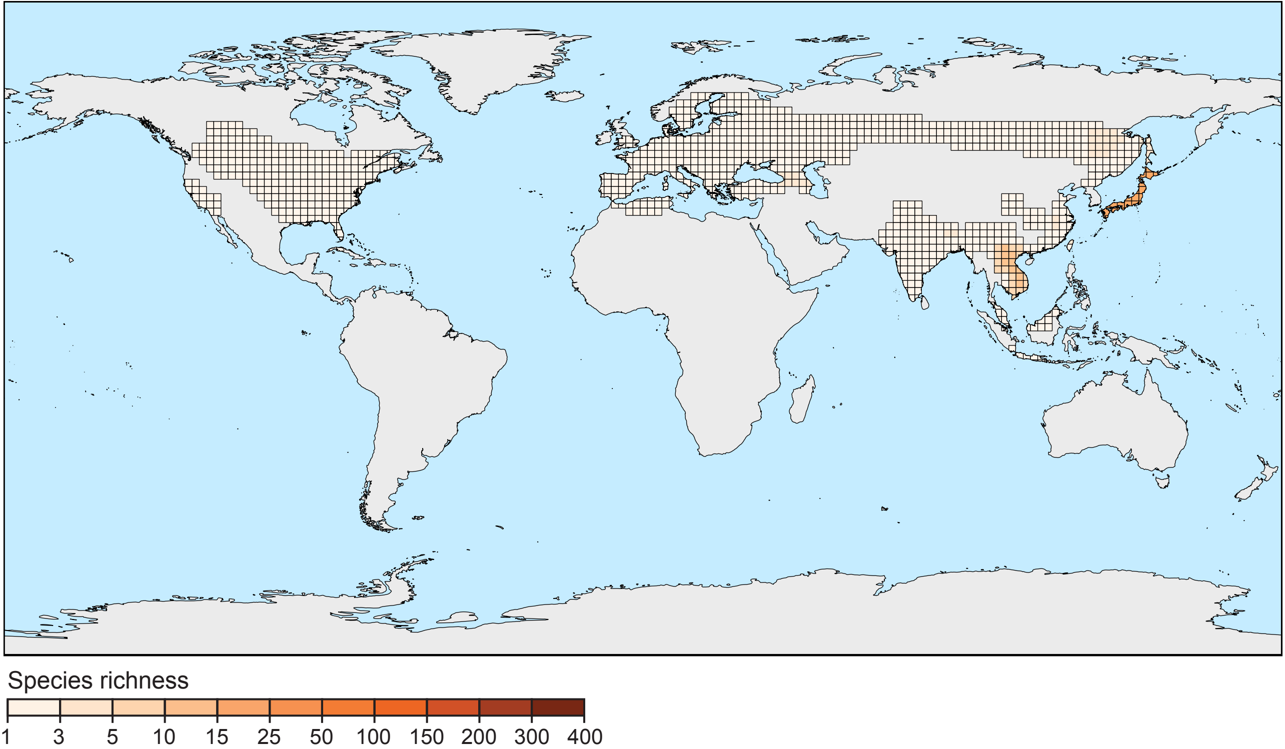

Fig. S14. Distribution and species diversity of the tribe Dictyopterini

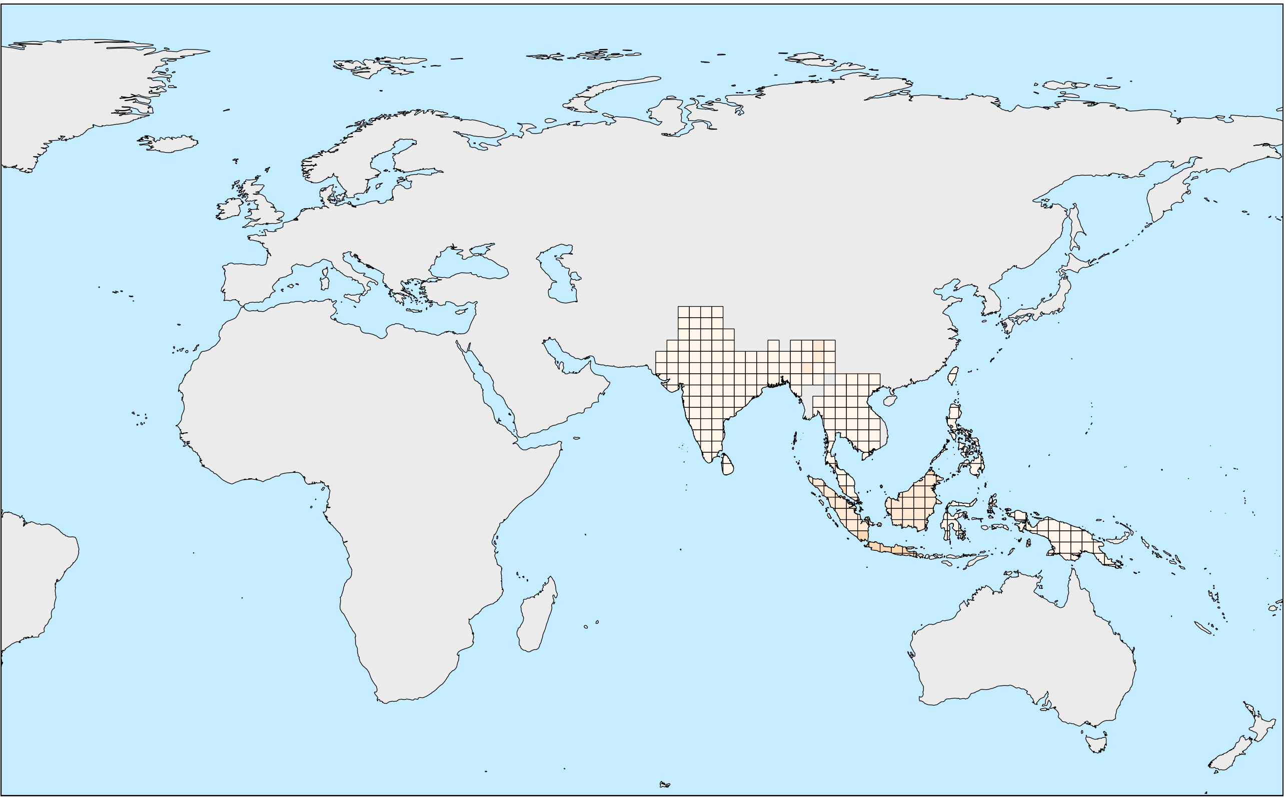

Fig. S15. Distribution and species diversity of the tribe Taphini

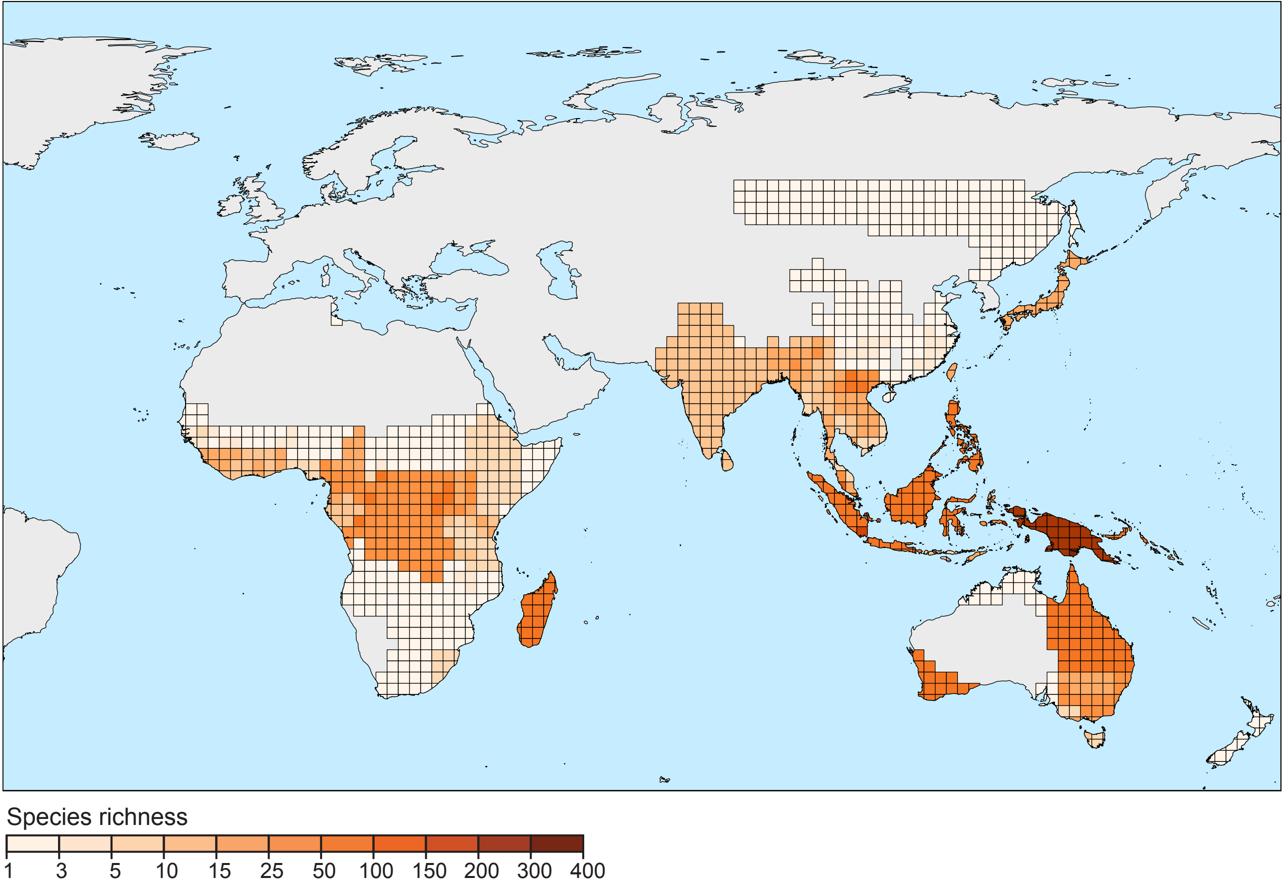

Fig. S16. Distribution and species diversity of the tribe Metriorrhynchini

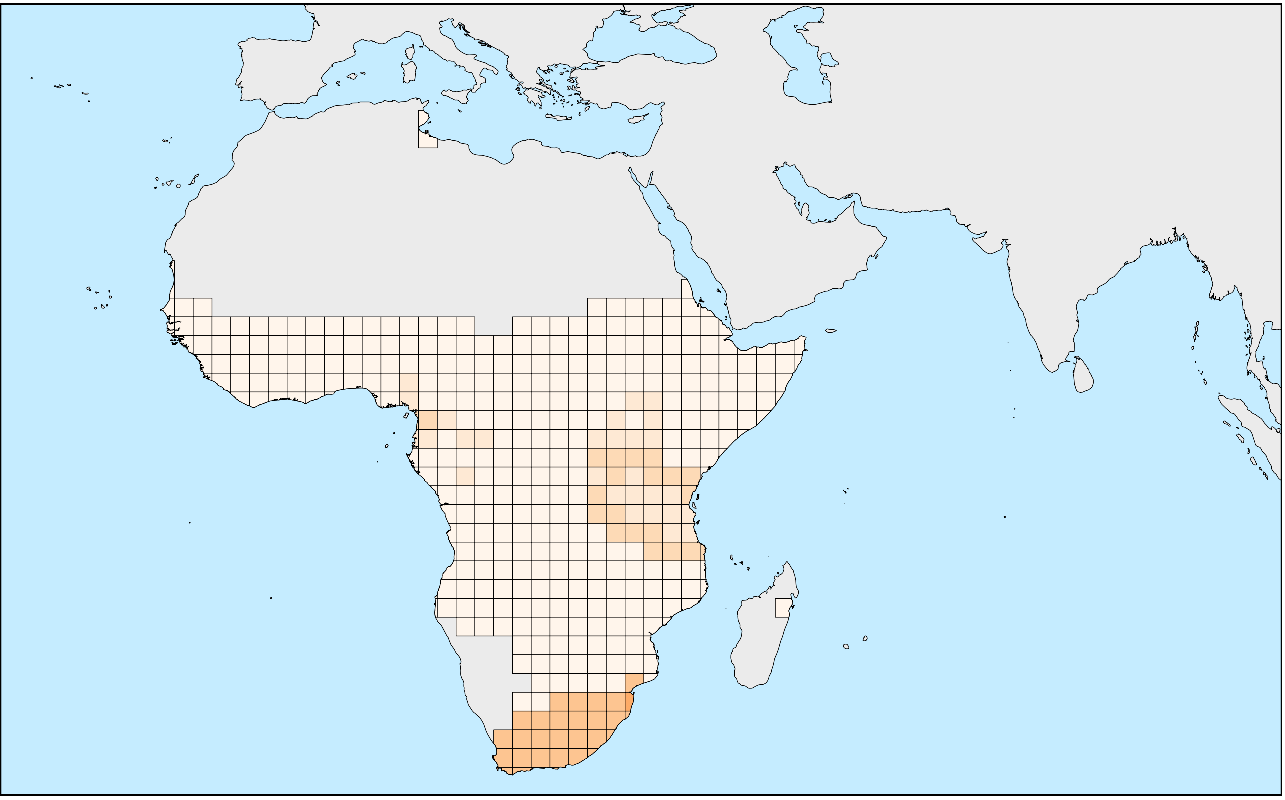

Fig. S17. Distribution and species diversity of the tribe Slipinskiini

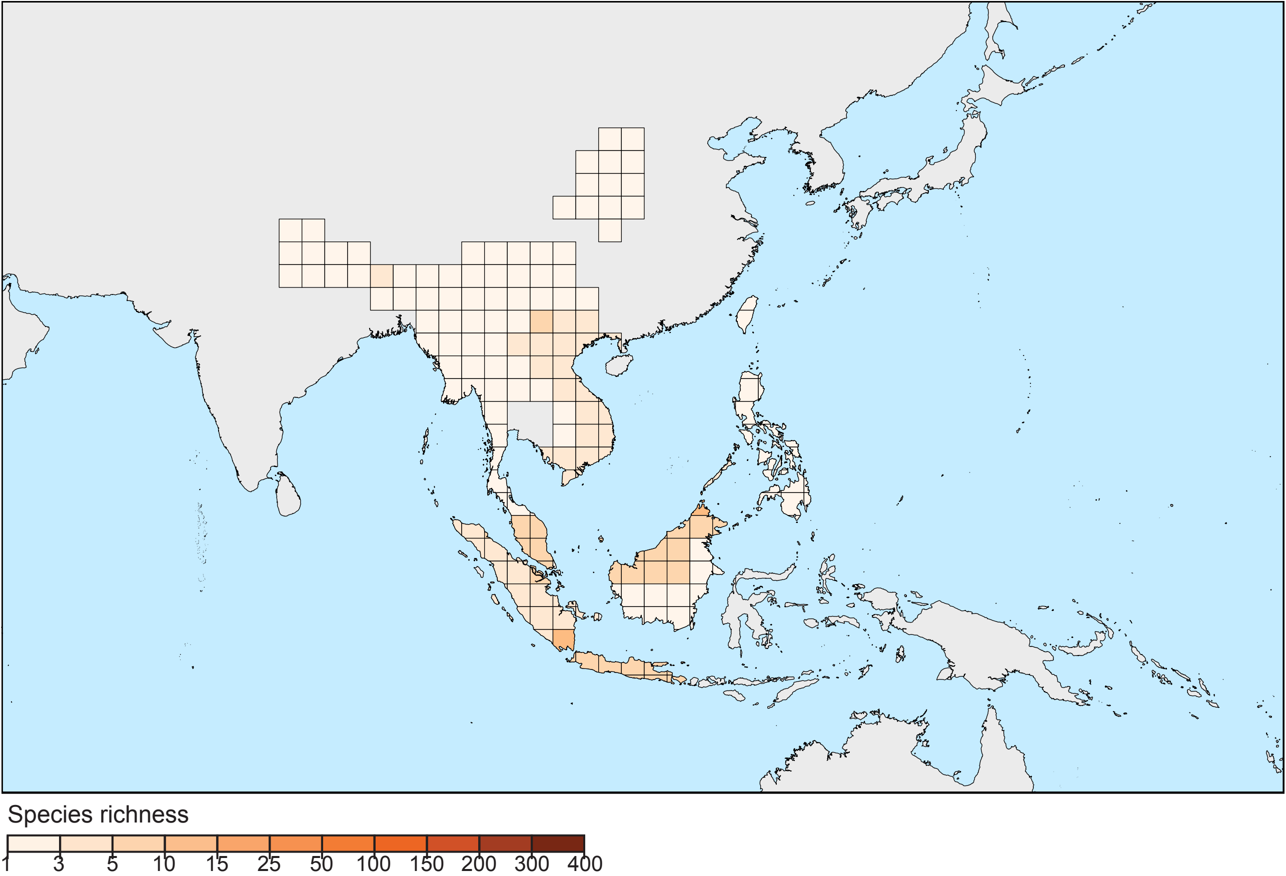

Fig. S18. Distribution and species diversity of the tribe Dihammatini

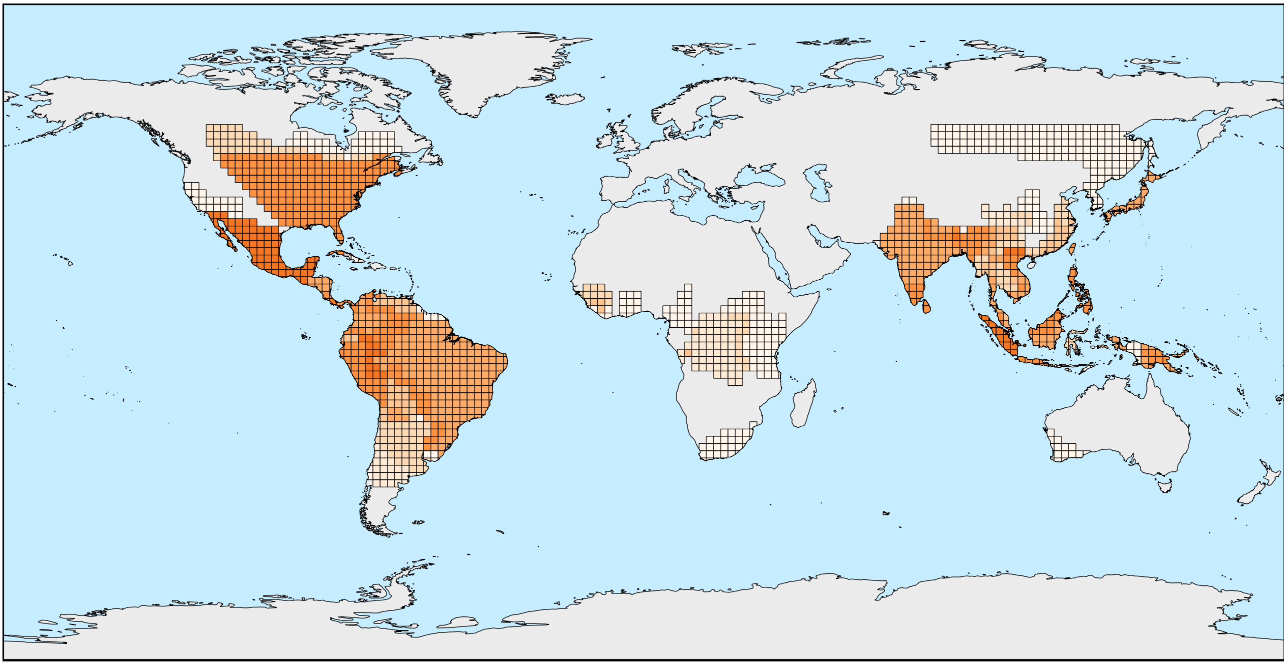

Fig. S19. Distribution and species diversity of the tribe Platerodini

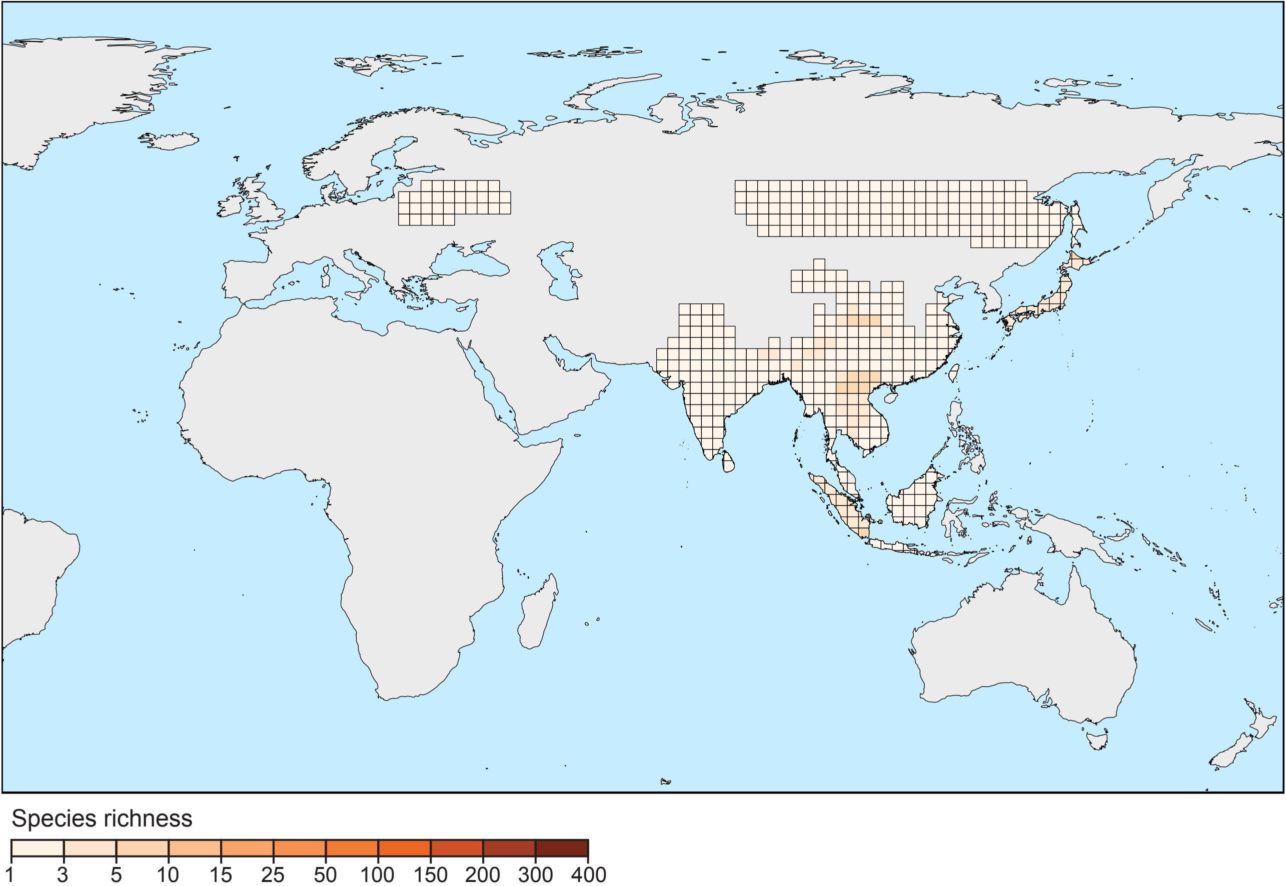

Fig. S20. Distribution and species diversity of the tribe Conderini

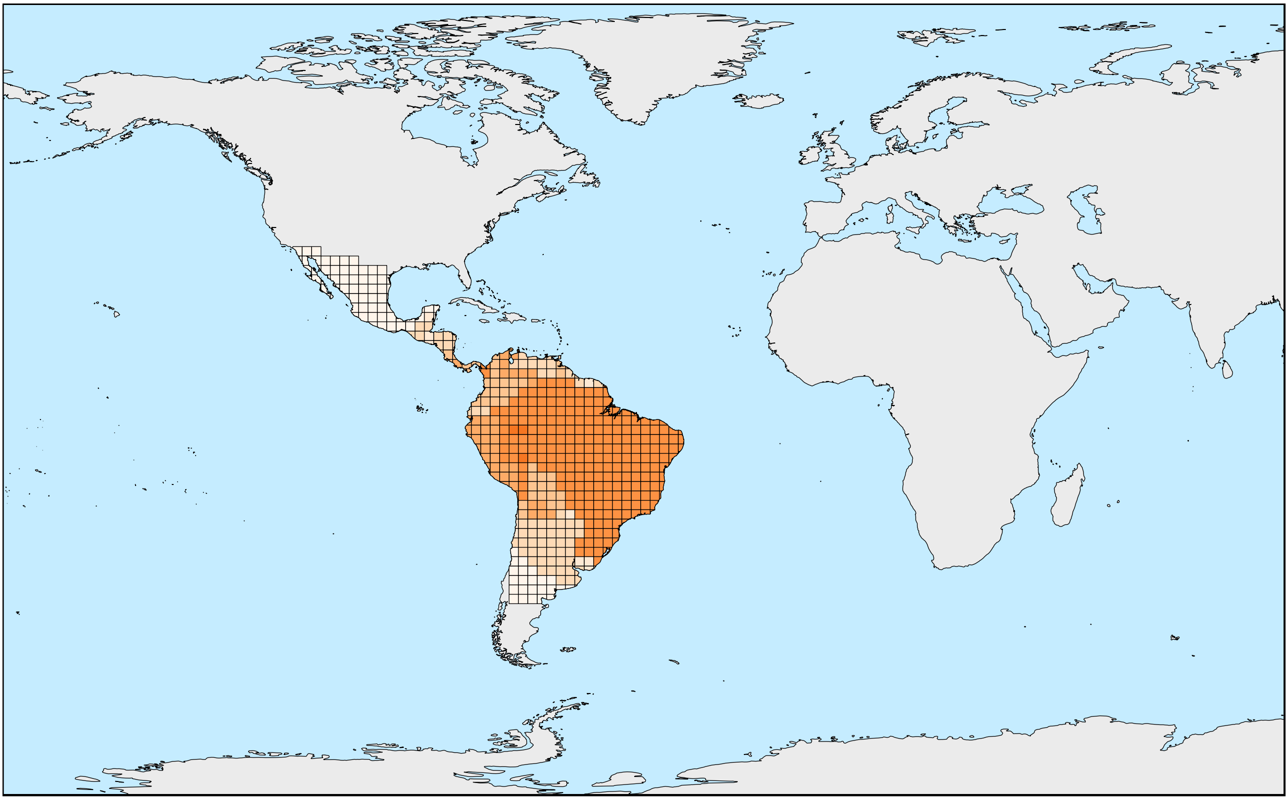

Fig. S21. Distribution and species diversity of the tribe Eurhacini

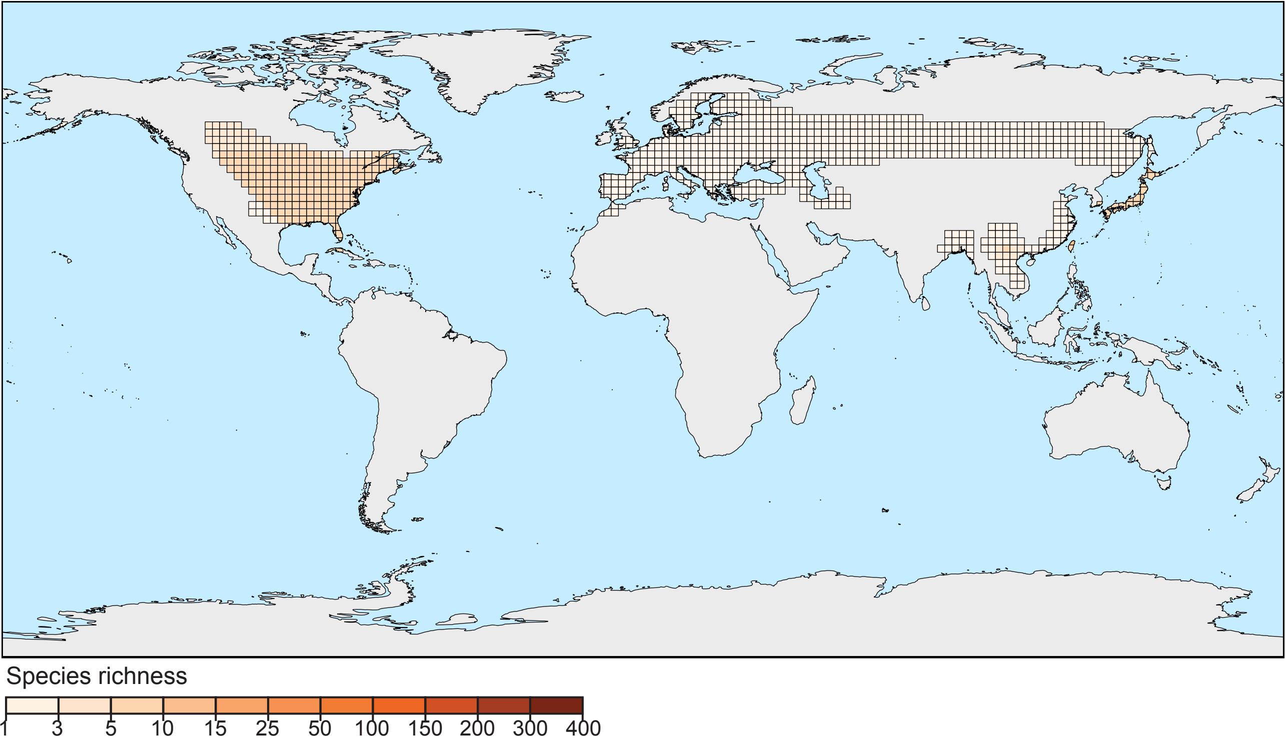

Fig. S22. Distribution and species diversity of the tribe Erotini

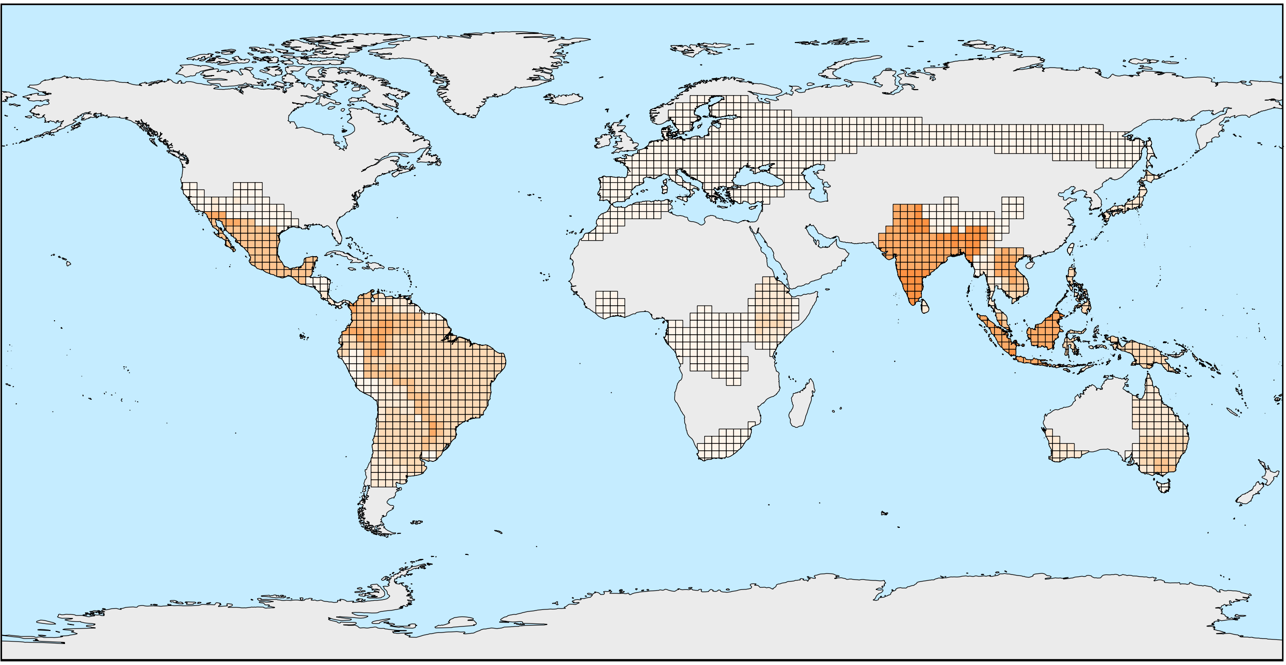

Fig. S23. Distribution and species diversity of the tribe Calochromini

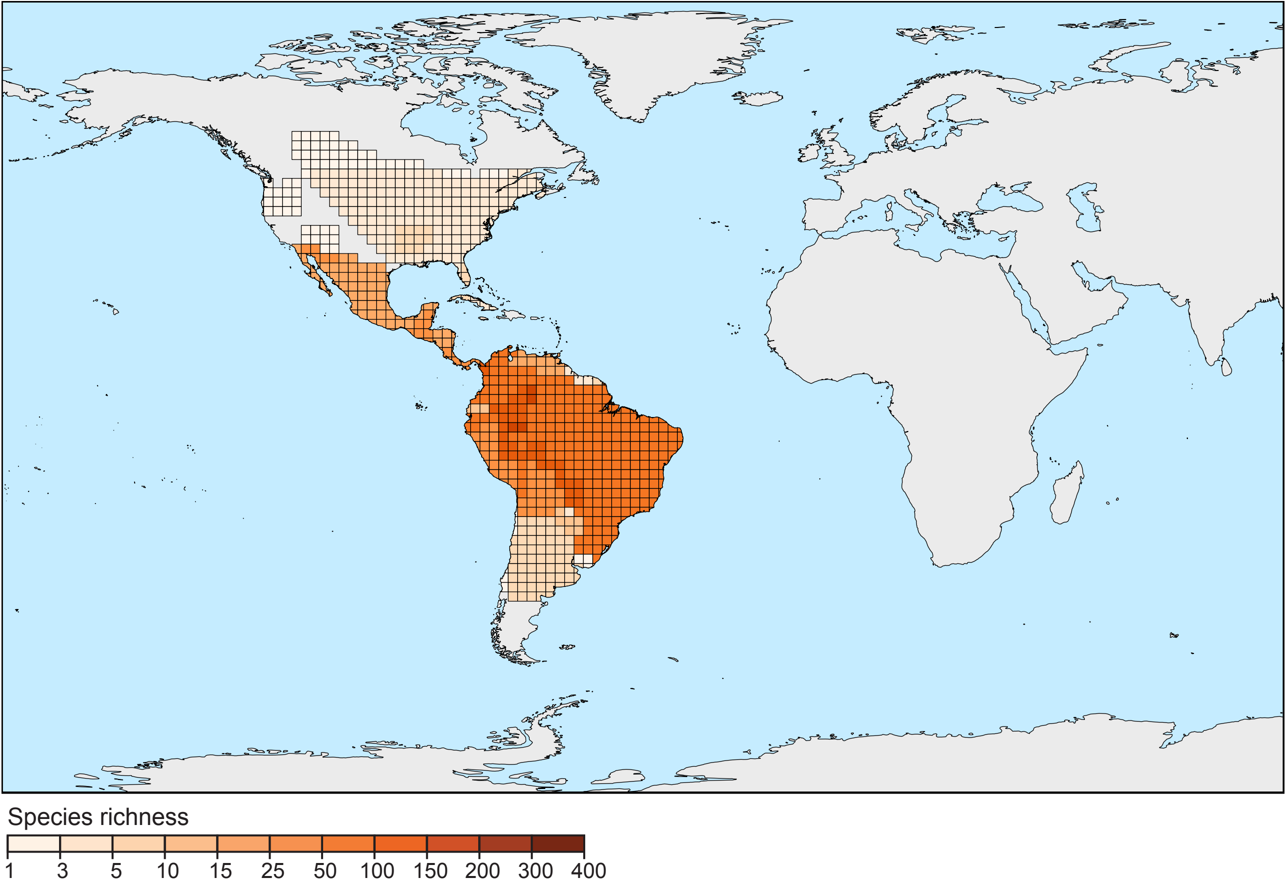

Fig. S24. Distribution and species diversity of the tribe Calopterini

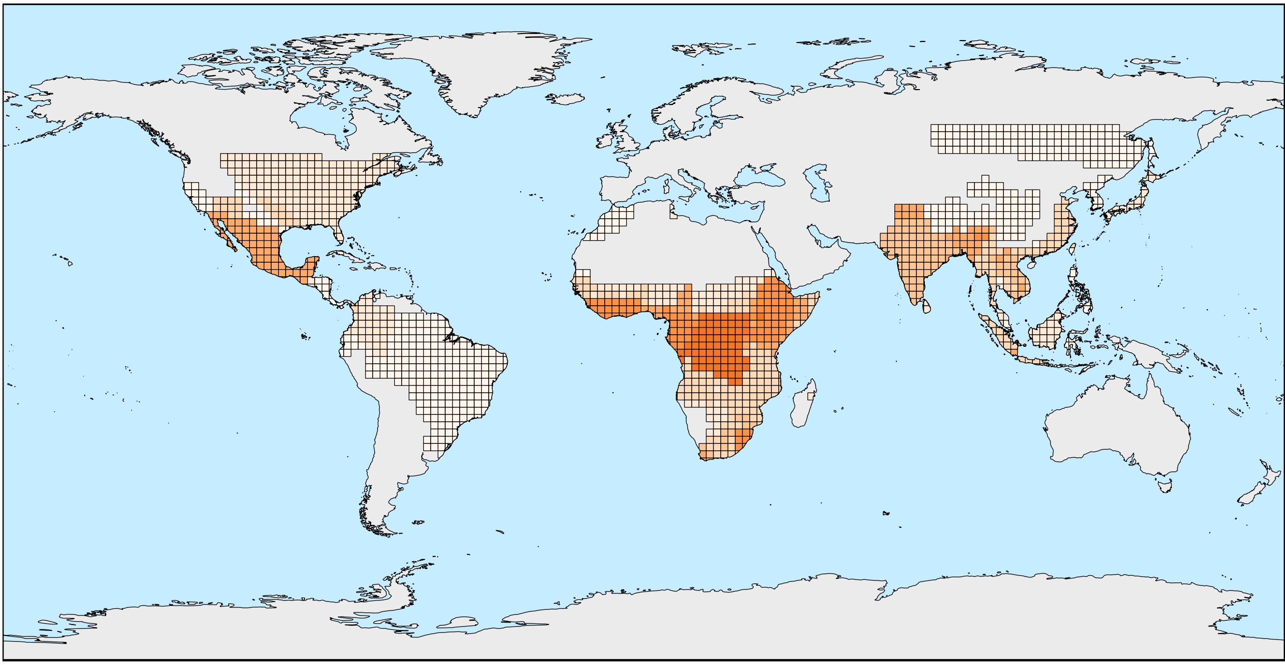

Fig. S25. Distribution and species diversity of the tribe Lycini

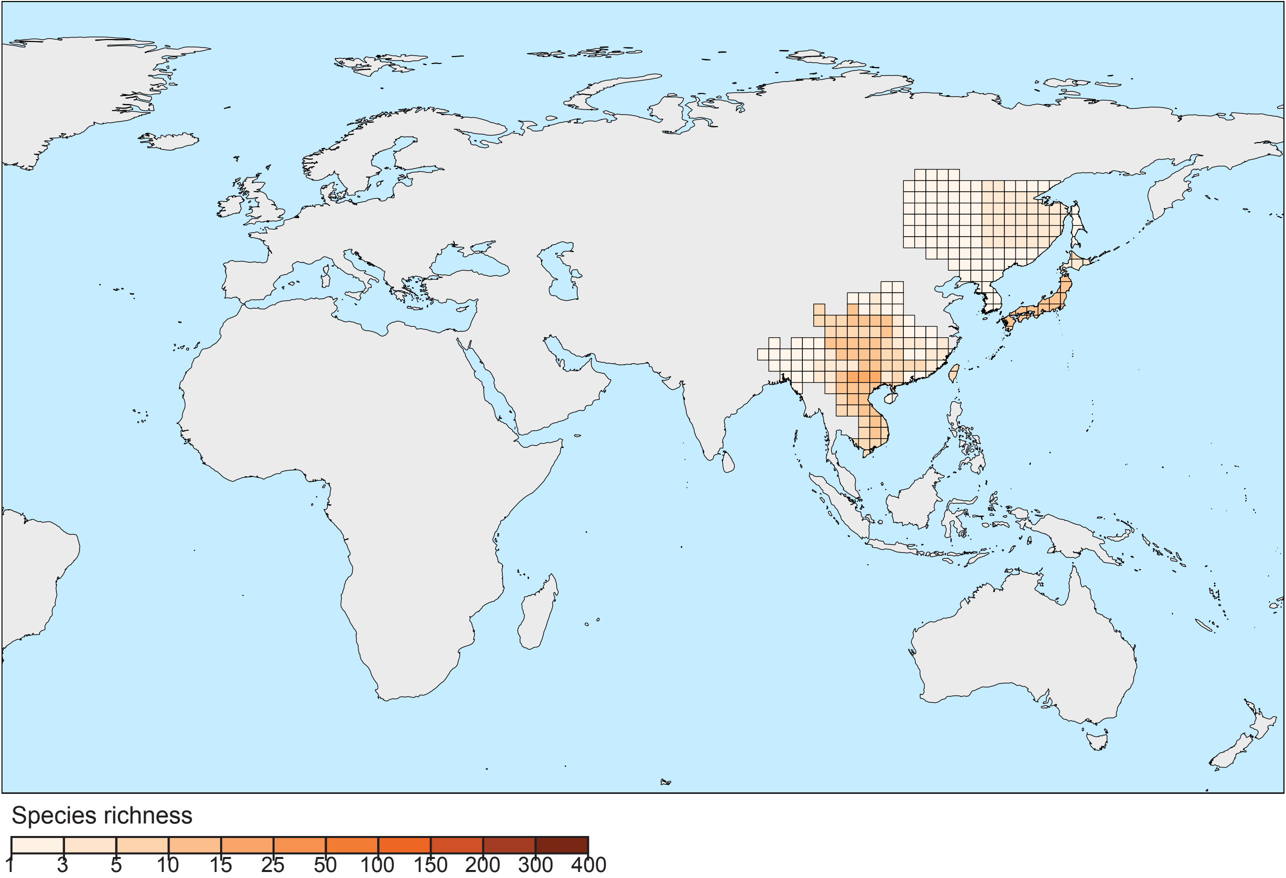

Fig. S26. Distribution and species diversity of the tribe Macrolycini

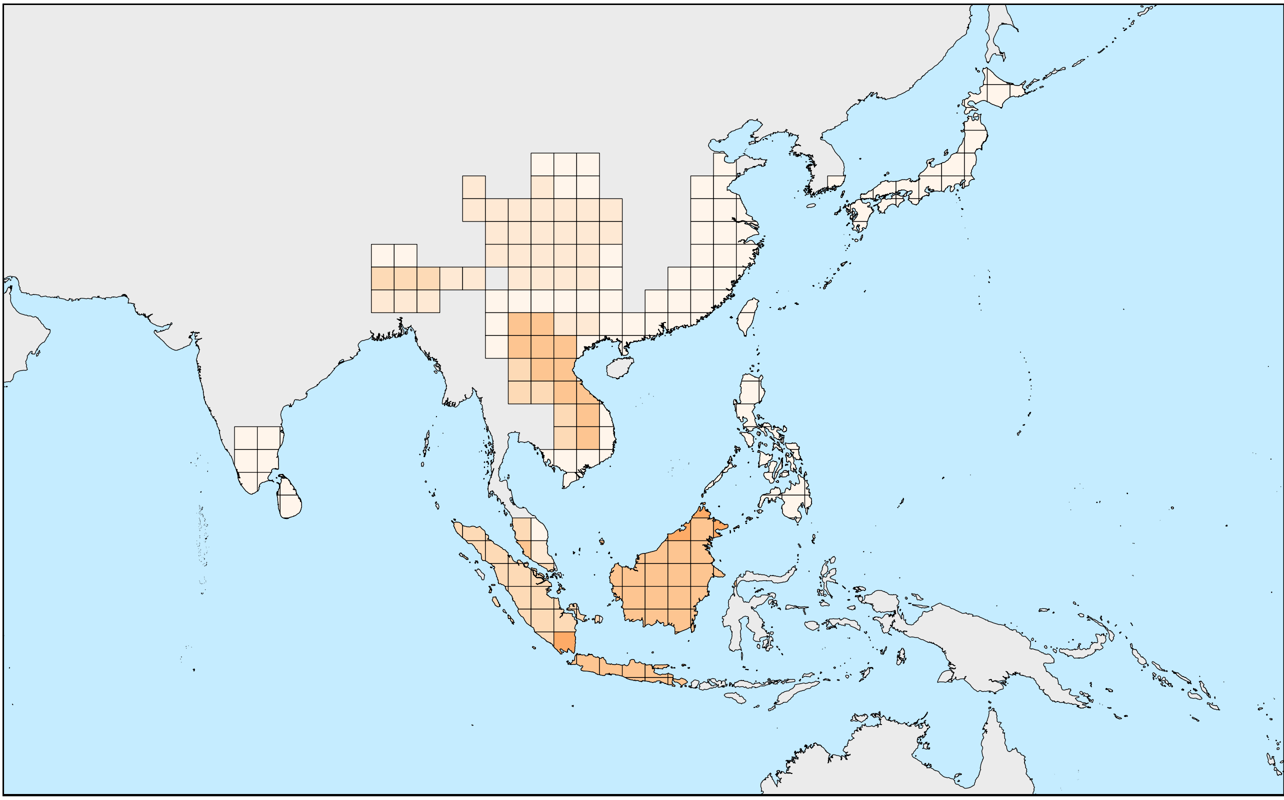

Fig. S27. Distribution and species diversity of the tribe Dilophotini

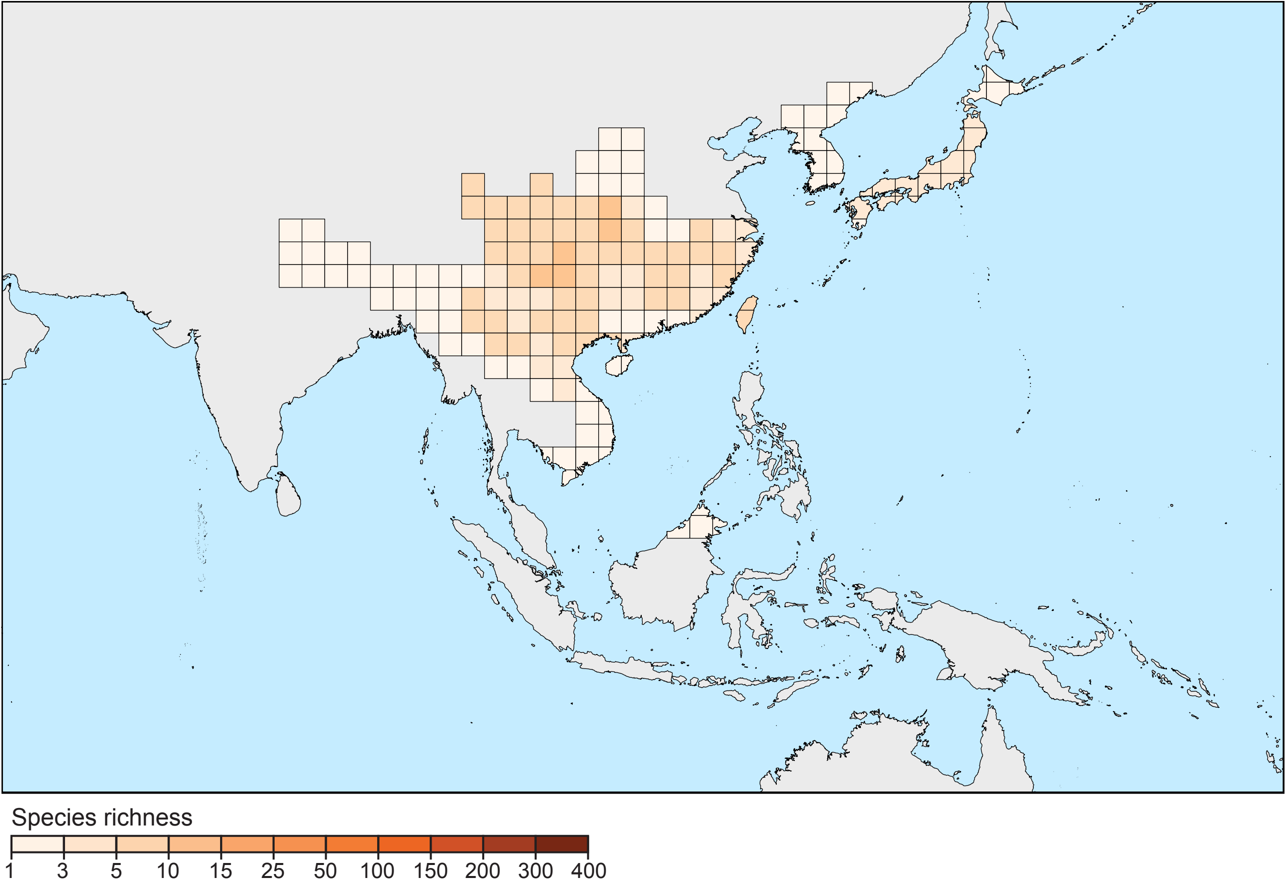

Fig. S28. Distribution and species diversity of the tribe Lyponiini

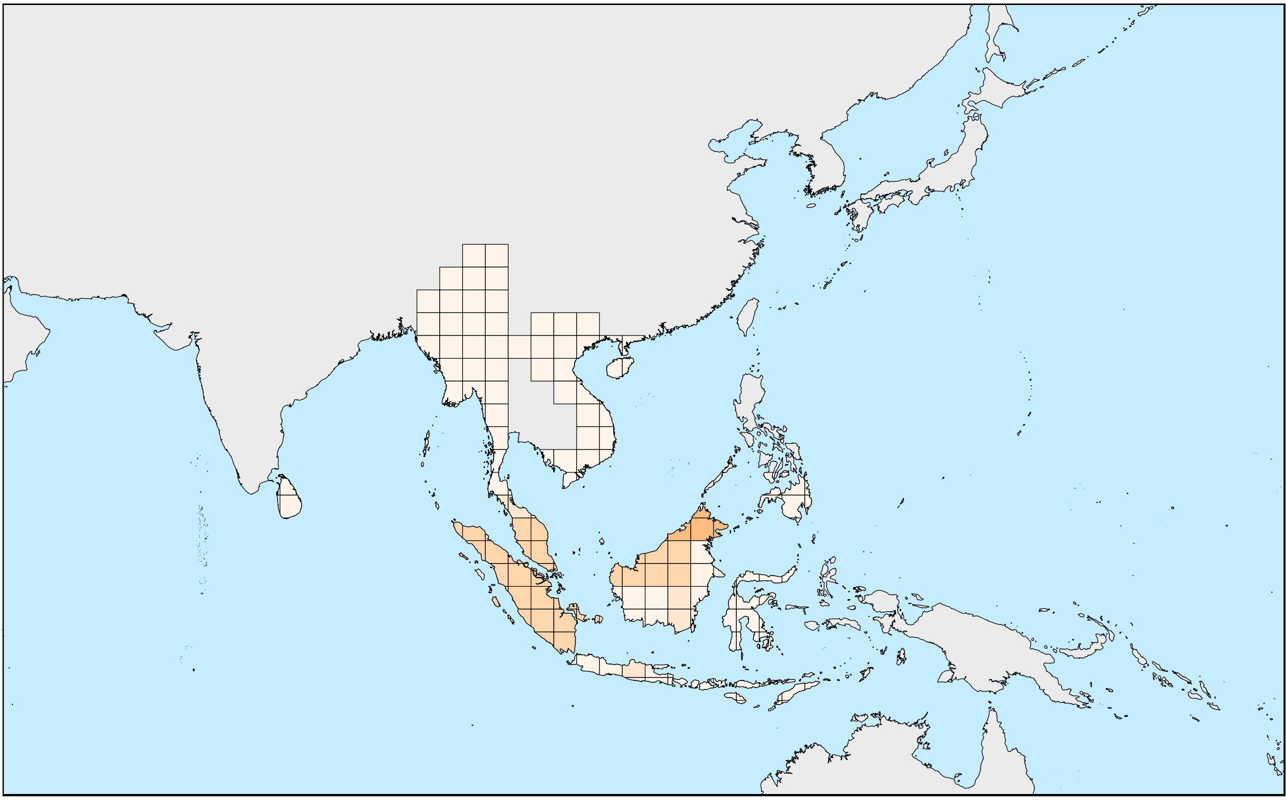

Fig. S29. Distribution and species diversity of the tribe Ateliini

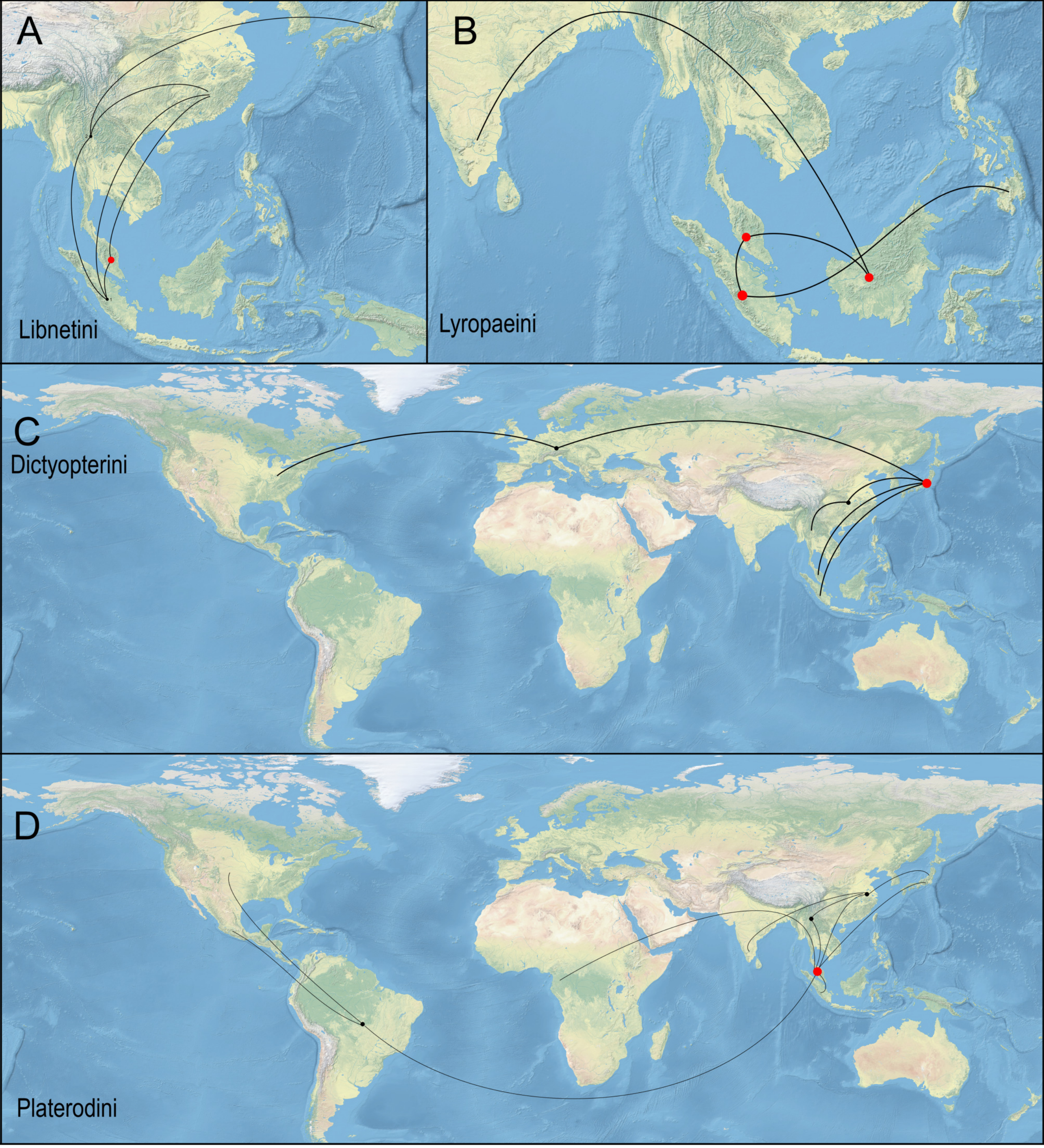

Figure S30. Identification of ancestral areas A – Libnetini, B – Lyropaeini, C – Dictyopterini, E – Platerodini.

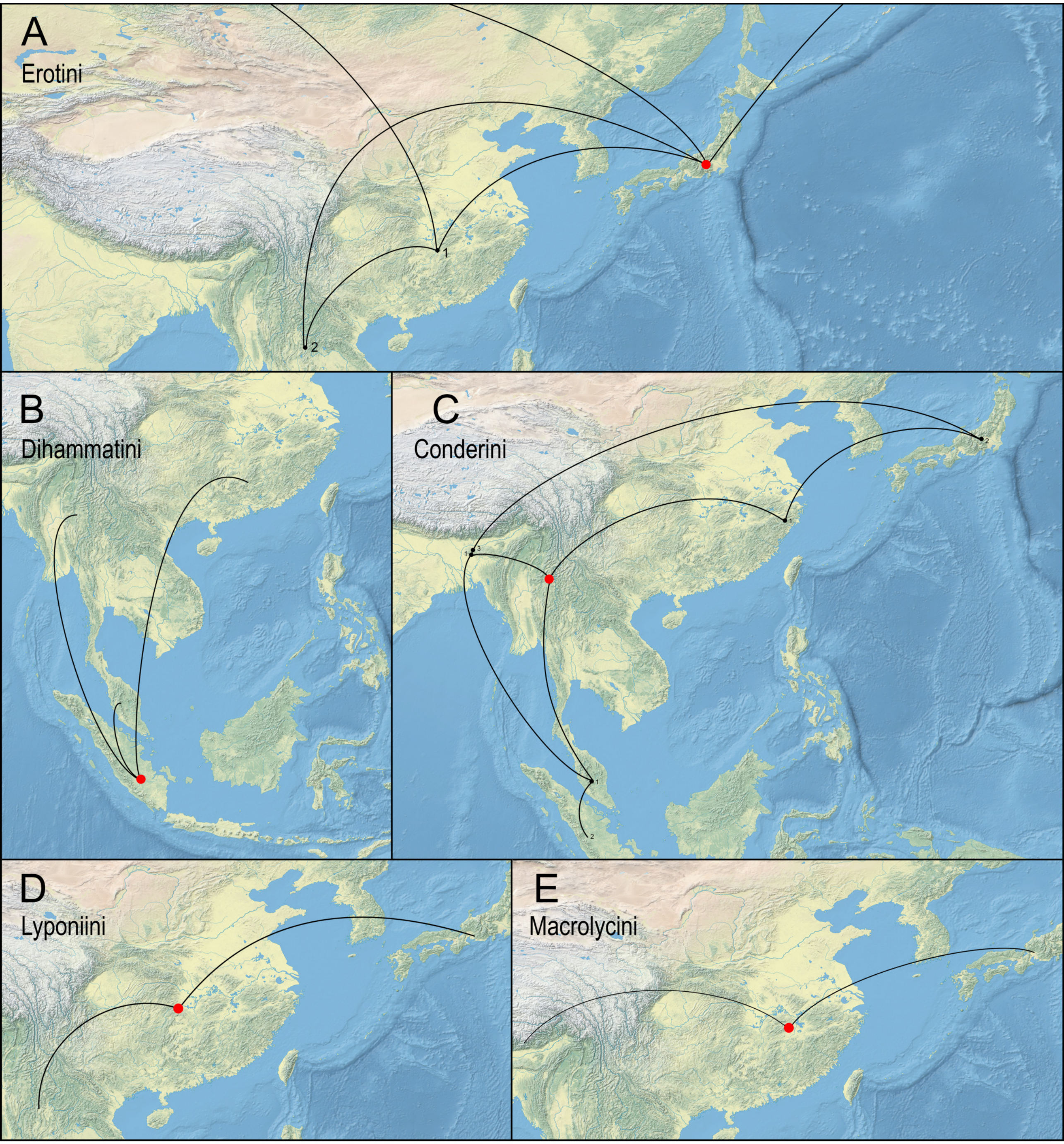

Figure S31. Identification of ancestral areas A – Erotini, B – Dihammatini, C– Conderini, D – Lyponiini, E – Macrolycini.
